# Laser processing in liquids: insights into nanocolloid generation and thin film integration for energy, photonic, and sensing applications

**DOI:** 10.3762/bjnano.16.104

**Published:** 2025-08-27

**Authors:** Akshana Parameswaran Sreekala, Pooja Raveendran Nair, Jithin Kundalam Kadavath, Bindu Krishnan, David Avellaneda Avellaneda, M R Anantharaman, Sadasivan Shaji

**Affiliations:** 1 Facultad de Ingeniería Mecánica y Eléctrica, Universidad Autónoma de Nuevo León. San Nicolás de los Garza, Nuevo León, 66455, México; 2 Current affiliation: Instituto de Ciencias Aplicadas y Tecnología, Universidad Nacional Autonoma de Mexico, Circuito Exterior S/n, Ciudad Universitaria, Ciudad de Mexico, C.P. 04510, Mexico; 3 Facultad de Ciencias Físico Matemáticas, Universidad Autónoma de Nuevo León, San Nicolás de los Garza, Nuevo León, 66455, México; 4 Centro de Innovación, Investigación y desarrollo en Ingeniería y Tecnología (CIIDIT), Universidad Autónoma de Nuevo León., PIIT Monterrey, Apodaca, Nuevo León, 66629, México; 5 School of Nanoscience and Nanotechnology, Mahatma Gandhi University, Kottayam 686560, Kerala, India

**Keywords:** HER/OER/water splitting, laser synthesis of nanomaterials, nanocolloids to thin films, photocatalysis, photovoltaics and photodetection, surface-enhanced Raman spectroscopy (SERS)

## Abstract

Nanoparticles in their pure colloidal form synthesized by laser-assisted processes such as laser ablation/fragmentation/irradiation/melting in liquids have attained much interest from the scientific community because of their specialties like facile synthesis, ultra-high purity, biocompatibility, colloidal stability in addition to other benefits like tunable size and morphology, crystalline phases, new compounds and alloys, and defect engineering. These nanocolloids are useful for fabricating different devices mainly with applications in optoelectronics, catalysis, sensors, photodetectors, surface-enhanced Raman spectroscopy (SERS) substrates, and solar cells. In this review article, we describe different methods of nanocolloidal synthesis using laser-assisted processes and corresponding thin film fabrication methods, particularly those utilized for device fabrication and characterization. The four sections start with an introduction to the common laser-assisted synthesis for nanocolloids and different methods of thin film fabrication using these nanocolloids followed by devices fabricated and characterized for applications including photovoltaics, photodetectors, catalysis, photocatalysis, electrochemical/photoelectrochemical sensors, hydrogen/oxygen evolution, SERS sensors and other types of devices reported so far. The last section explains the challenges and further scope of these devices from laser-generated nanocolloids.

## Review

### 1 Introduction

**Figure 1:**
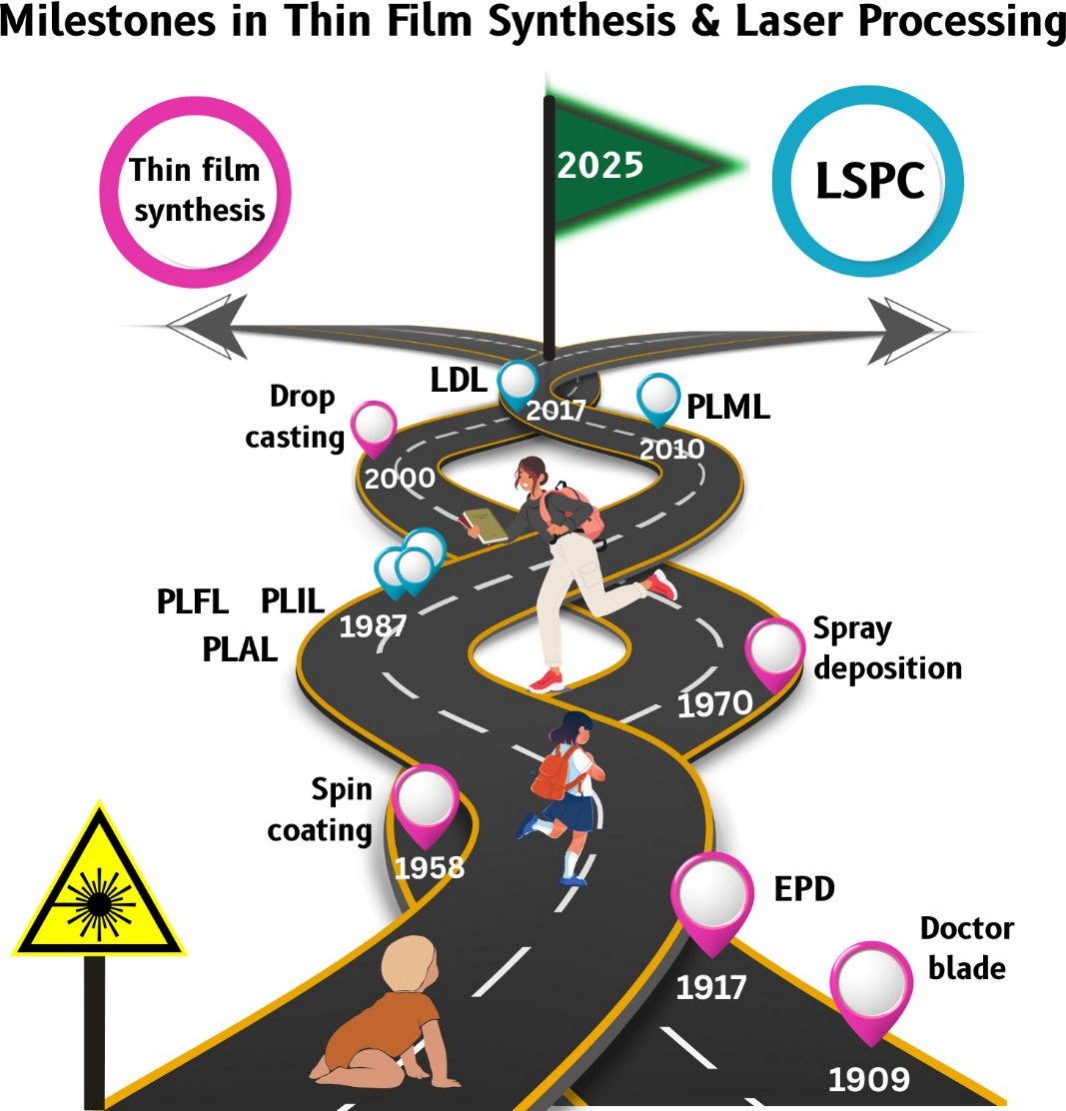
A visual roadmap tracing key advancements in thin film deposition methods and laser-based material processing [3]. All graphical elements including road, human beings, pinpoints and flag: ©Pooja Raveendran Nair and Akshana Parameswaran Sreekala via Canva.com.

This section provides a brief introduction to the fundamental laser processing techniques used in liquids, including ablation, fragmentation, melting, irradiation; it also touches upon the phenomenon of laser-induced defects in liquid environments. While numerous comprehensive reviews on these topics already exist, this article aims to offer a succinct overview of the fundamental principles and applications of these techniques [1]. The focus is on providing a foundational understanding, without investigating the extensive details that other reviews have already explored. The fundamental chemical and physical aspects of pulsed laser ablation (PLA) processes, with a focus on the evolution of material from the target to the deposited film, were explained by Ashfold et al. [2] in one of the first reviews on laser ablation published in 2004. Their discussion addresses the physicochemical insights into key stages of these processes, including the behavior of the laser target, the formation of the plume, and the initial laser–target interactions. It offers a comprehensive understanding of the process flow, from the laser-induced ejection of material into the gas phase, through its processing and movement in the plume, to its eventual deposition onto a substrate. Figure 1 shows key developments in thin film synthesis and laser-based processing from 1909 to 2025. These techniques have been important for progress in this field of study.

#### 1.1 Laser ablation in liquids

Laser ablation in liquids (LAL) is a well-established technique for synthesizing nanomaterials such as metals, semiconductors, ceramics, polymers, and alloys. Several review articles published since 2004 [2,4–9] offer insights into its history, novelties, mechanisms, and applications, catering to the interests and choices of readers [10]. A list of recent review articles highlighting advancements in laser ablation, processing, and structuring of different materials, along with a detailed discussion of their resulting applications is also available [11].

**Figure 2:**
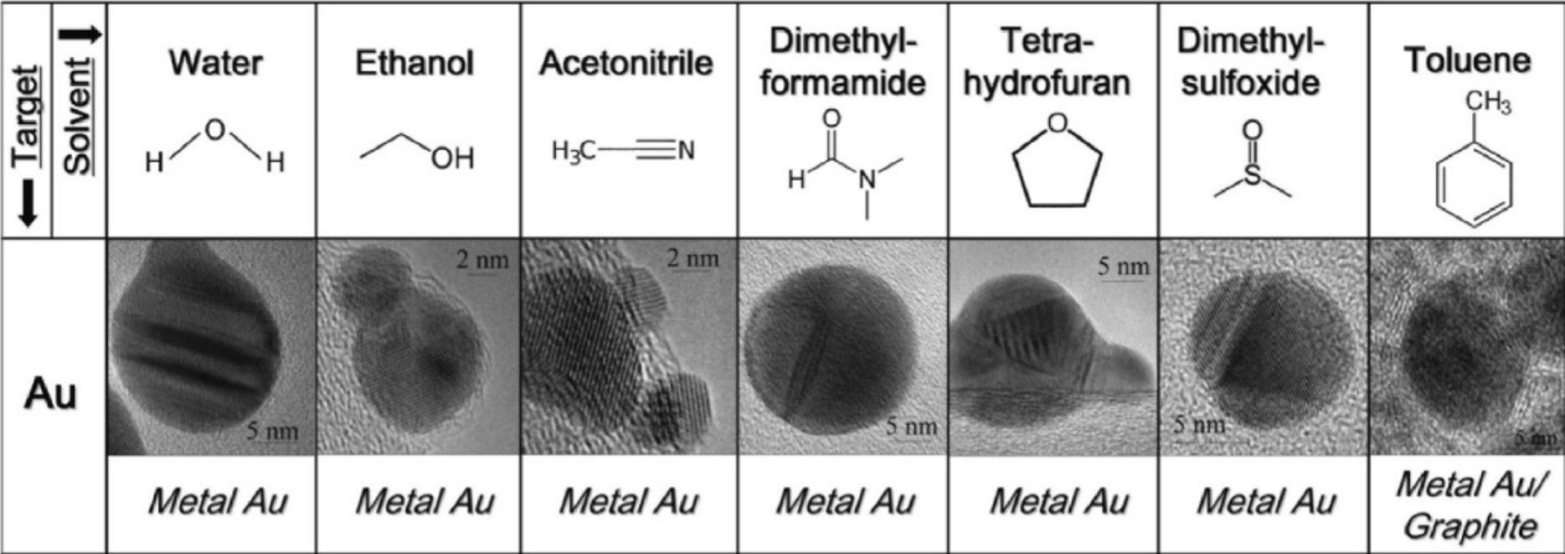
Morphologies of Au NPs prepared by PLA in various solvents. Figure 2 was used with permission of The Royal Society of Chemistry, from [16] (“What controls the composition and the structure of nanomaterials generated by laser ablation in liquid solution?” by V. Amendola and M. Meneghettia, *Phys. Chem. Chem. Phys*., vol. 15, issue 9, © 2013); permission conveyed through Copyright Clearance Center, Inc., This content is not subject to CC BY 4.0.

One of the earlier review articles (published in the year 2012) on laser ablation/irradiation explains how to attain nanostructures with various compositions (e.g., metals, alloys, oxides, carbides, and hydroxides) and morphologies (e.g., nanoparticles, nanocubes, nanorods, and nanocomposites) [5]. The developments in LAL have demonstrated its ability to synthesize functionalized nanostructures with special morphologies, microstructures, and phases, which hold promise for applications in optics, detection, and biological fields [12]. The size, shape, and composition of these nanomaterials can be controlled by adjusting experimental parameters such as laser settings, liquid medium, and target material. Additionally, the preparation of doped and multicomponent semiconductor nanostructures could expand LAL’s potential in catalysis, solar energy, and light emission. A critical review on the emerging topic of laser ablation, fragmentation, and melting in liquids, and key reports on both the fundamental principles and applications related to these processes are available in [6]. In another review, the formation mechanism and examples of nanoparticle synthesis by laser ablation were discussed [7]. They have clearly depicted how the properties of the nanoparticles (NPs) depend strongly on size, shape, and size uniformity. Pulsed laser ablation in liquids (PLAL) is a promising method for producing pristine and supported materials including mono- and bimetallic NPs with distinctive structural features such as unique catalytic and plasmonic properties that cannot be achieved by conventional methods [13–14]. The latest review on gold-based NPs by PLAL discusses how laser parameters (e.g., wavelength, pulse duration, and fluence) and medium characteristics (e.g., chemical composition and viscosity) influence the size, morphology, and surface properties of the Au NPs, which, in turn, determine their performance. The effect of solvents on morphology of generated NPs in laser ablation is shown in Figure 2. While PLAL offers a rich combinatorial library of constituents and interactions [15], understanding of these processes remains insufficient. Yan et al. outline a comprehensive mechanistic scenario of PLAL, highlighting the interactions between photons, liquid molecules, solid targets, and laser-induced particles [8].

The formation mechanism of NPs in PLAL has been investigated extensively, primarily through the use of laser-induced fluorescence analysis and shadowgraph analysis [17–21]. Use of small-angle X-ray scattering, wide-angle X-ray scattering, and X-ray imaging techniques has enabled a more comprehensive exploration of the nanoparticle formation mechanism. These advanced analytical methods offer higher temporal and spatial resolutions compared to conventional techniques [22–25]. A recent review by Byram et al. [11] highlights the significant advancements made in PLAL over the past decade using laser pulses of varying durations (nanoseconds, picoseconds, and femtoseconds), the influence of laser parameters (wavelength, fluence, and pulse duration) and surrounding environments on the morphology of nanostructures focusing on nanomaterial synthesis and their applications in sensing and photonics. The study also delves into the preparation of novel nanomaterials like HfO_2_ NPs, few-layer graphene, and Cu/CuO/Fe_3_O_4_ composites using LAL, opening new avenues in both sensing and photonics.

Looking ahead, studies focusing on the formation of functionalized nanostructures with tailored properties for specific applications will be crucial for realizing the full potential of laser-based techniques and addressing challenges in LAL such as low productivity, high energy consumption, and wide size distribution. Additionally, advancing in situ characterization methods, such as time-resolved shadowgraphy [26] and optical monitoring, could provide deeper insights into the ablation process and facilitate more precise control over the material properties. Overall, laser processing in liquids (LPL) remains a rapidly evolving and promising field [27], with significant opportunities for future innovation in nanomaterial synthesis and device fabrication [28].

#### 1.2 Laser fragmentation in liquids

Laser fragmentation in liquids (LFL) is an innovative and environmentally friendly technique that offers precise control over the size and distribution of NPs and atomic clusters. This method involves the use of short-pulse laser irradiation to fragment larger NPs suspended in a colloidal solution, resulting in smaller NPs with a narrow size distribution. LFL is particularly beneficial as a post-processing tool for NPs produced by PLAL, a technique which often results in broader or bimodal size distributions as discussed in the previous section. Unlike traditional post-processing methods such as centrifugation or salinity size quenching, LFL provides a cost-effective and efficient alternative. It excels at producing ultrasmall NPs with diameters below 5 nm and sub-nanometer atomic clusters, which have significant potential in applications like catalysis. This technique has become a valuable addition to the toolbox of nanoparticle synthesis, offering enhanced precision and scalability for advanced materials and applications [6,29–30].

**Figure 3:**
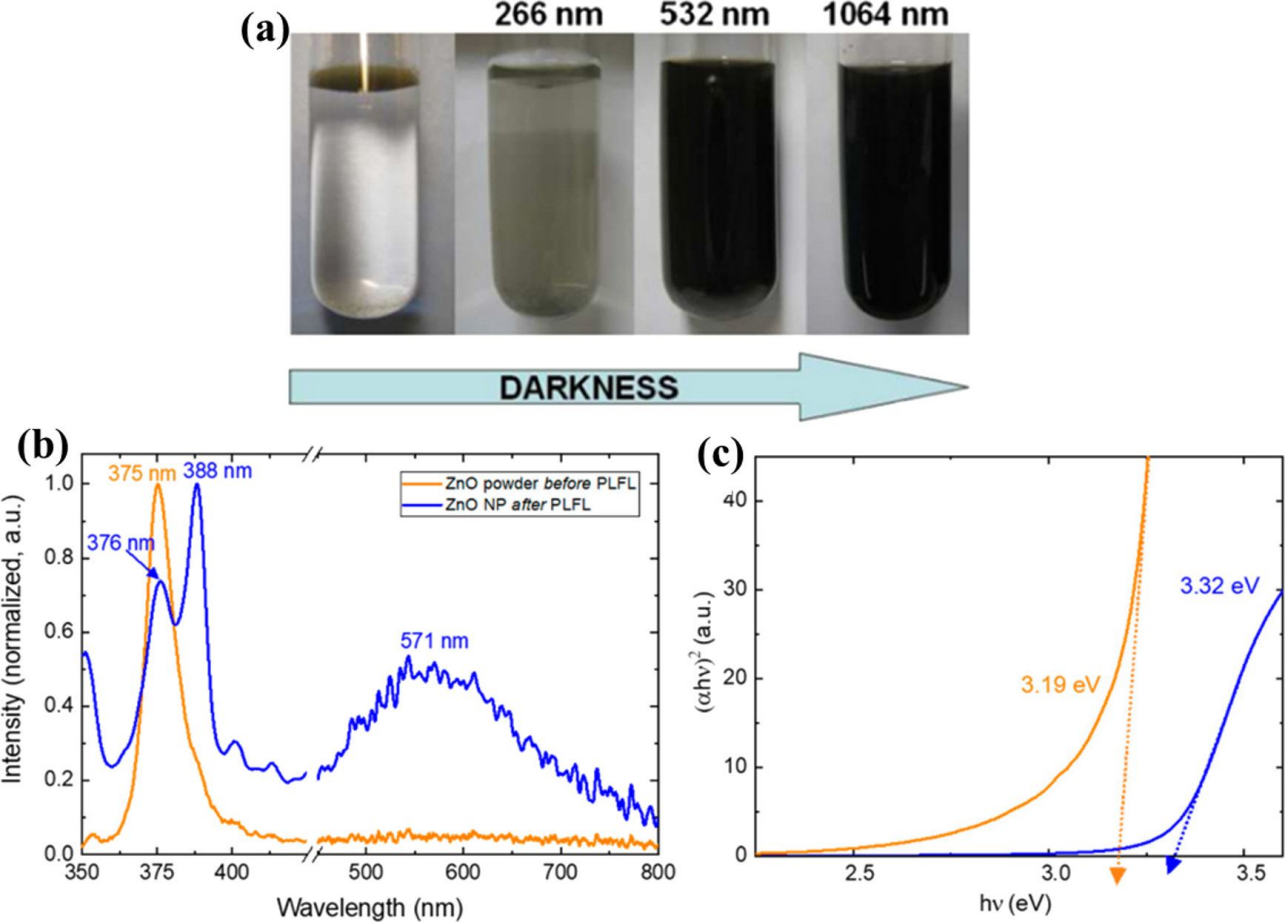
(a) Influence of the wavelength on the concentration of fragmented particles after 5 min of irradiation with UV (266 nm), visible (532 nm), and IR (1064 nm) at 120 mJ. (b) Photoluminescence spectra and (c) Tauc plots for the ZnO nanoparticles both before (orange lines) and after (blue lines) PLFL processing. Figure 3a was reprinted from [31], *Journal of Colloid and Interface Science*, vol. 357, by C. Chubilleau; B. Lenoir; S. Migot; A. Dauscher, “Laser fragmentation in liquid medium: A new way for the synthesis of PbTe nanoparticles”, pages 13–17, Copyright (2011), with permission from Elsevier. This content is not subject to CC BY 4.0. Figure 3b,c were reproduced from [33] (© 2020 K. Charipar et al., published by MDPI, distributed under the terms of the Creative Commons Attribution 4.0 International License, https://creativecommons.org/licenses/by/4.0).

The synthesis of lead telluride (PbTe) NPs was successfully performed through pulsed laser fragmentation of PbTe micrometer-sized powders in distilled water using a Nd:YAG laser [31]. The study examined the impact of various experimental parameters, including laser wavelength (Figure 3a), treatment duration, and output energy, on the fragmentation yield, nanoparticle size, and crystallographic structure. The findings suggest that smaller particle sizes are primarily achieved by increasing the number of laser pulses, though there is a limit to the number of pulses that should be applied. Furthermore, the energy output notably influenced the size distribution, with higher energy leading to a broader range of particle sizes compared to lower laser energy. Fujiwara et al. [32] also observed similar kind of features on thionicotinamide (TNA)-capped gold NPs irradiated at 532 nm (Q-switched Nd:YAG laser, 18 ps). TNA-capped gold NPs underwent fusion as well as fragmentation upon laser pulse excitation (532 nm). The morphological changes induced by thermal and photochemical effects were found to influence the optical properties of these particles. Another commonly reported semiconductor material by LFL is zinc oxide (ZnO) [33–34]. The optical properties, particularly the UV emission characteristics, are influenced by the NP size and crystalline quality (Figure 3b,c). LFL also plays a crucial role in tuning the optical properties of NPs. Laser processing affects the defect density and optical bandgap of the particles, as demonstrated by changes in the optical transmission spectra before and after LFL treatment. By adjusting processing parameters such as solvent choice, the defect emission, including green defect emissions, can be controlled, providing further opportunities for tailoring the optical properties of NPs for various applications.

**Figure 4:**
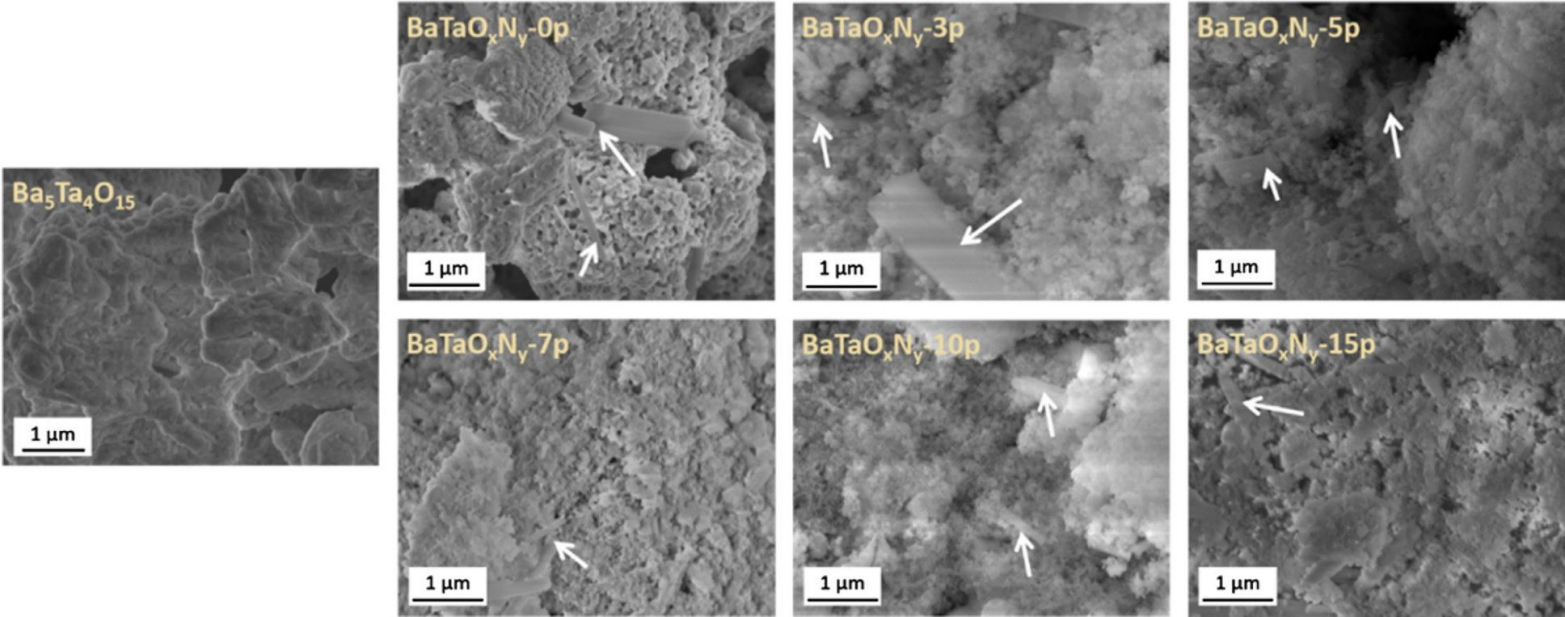
SEM images of Ba_5_Ta_4_O_15_ and BaTaO*_x_*N*_y_*-zp powders with increasing number of laser fragmentation passages. The arrows show the BaCO_3_ secondary phase. Figure 4 was reprinted from [40], *Applied Surface Science*, vol. 510, by F. Haydous; F. Waag; W. Si; F. Li; S. Barcikowski; B. Gökce; T. Lippert, “The effect of downstream laser fragmentation on the specific surface area and photoelectrochemical performance of barium tantalum oxynitride”, article no. 145429, Copyright (2020), with permission from Elsevier. This content is not subject to CC BY 4.0.

The mechanisms behind nanoparticle formation from powders remain unclear to date. Drawing a parallel with PLAL, Chubilleau et al. offer some insight, although this analogy may not be fully applicable as each fragment behaves like a small, moving target [31]. The mechanism of laser fragmentation of noble metal NPs has been attributed to photoelectron ejection induced by laser shots, which leaves positive charges on the surface [35–36]. These surface charges cause electrostatic repulsion between different parts of the material, leading to fragmentation and the formation of smaller particles. The study on Au NP fragmentation [37] highlights the significance of photon-assisted transitions in fragmentation processes, particularly when interacting with the second and third harmonics of a picosecond Nd:YAG laser. Although the temperature of the Au NPs exceeded the evaporation threshold, the results suggest that heating alone was not the primary cause of fragmentation. Additionally, the synthesis of heterostructured NPs composed of Au and Co using femtosecond laser techniques demonstrates the creation of metastable structures that can evolve into stable core–shell configurations [38]. These findings emphasize the role of non-equilibrium processes, rapid quenching, and the challenges of oxidation in nanoparticle synthesis. Cost-effective methods for modifying the surface characteristics of Si nanocrystals (Si-ncs) and BaTaO*_x_*N*_y_* perovskite particles using laser fragmentation are reported in [39] and [40], respectively. In particular, laser fragmentation in water successfully alters the surface properties of Si-ncs, making them hydrophilic and improving their dispersion in aqueous solutions, while also leading to the formation of self-organized networks. However, laser fragmentation of BaTaO*_x_*N*_y_* particles resulted not only in increased surface area but also caused oxidation, crystallinity loss, and reduced photoactivity due to enhanced grain boundaries. Figure 4 shows SEM images of Ba_5_Ta_4_O_15_ and BaTaO*_x_*N*_y_*-zp powders after successive laser fragmentation passages. Despite these challenges, prolonged fragmentation improved the photocurrent efficiency, suggesting that careful control of size reduction and surface properties is critical for optimizing photocatalyst performance, especially in water splitting applications. Further investigation is necessary to refine the process and mitigate the negative effects of fragmentation.

LFL is a highly versatile, effective method used for producing NPs with a narrow size distribution and tailored surface characteristics. While challenges such as oxidation and pulse limitations exist, its ability to create ultrasmall NPs and nanoclusters makes it particularly promising for applications in catalysis, material engineering, and energy storage. LFL can be applied to a wide range of materials, including metals such as gold, silver, or copper, semiconductors, and perovskites materials. The method is versatile enough to cater to the synthesis of NPs across different compositions, making it a valuable tool for researchers and industries seeking precise control over material properties. LFL can be utilized to produce larger volumes of nanocolloids even fulfilling the demands for industrial applications.

#### 1.3 Laser melting in liquids

**Figure 5:**
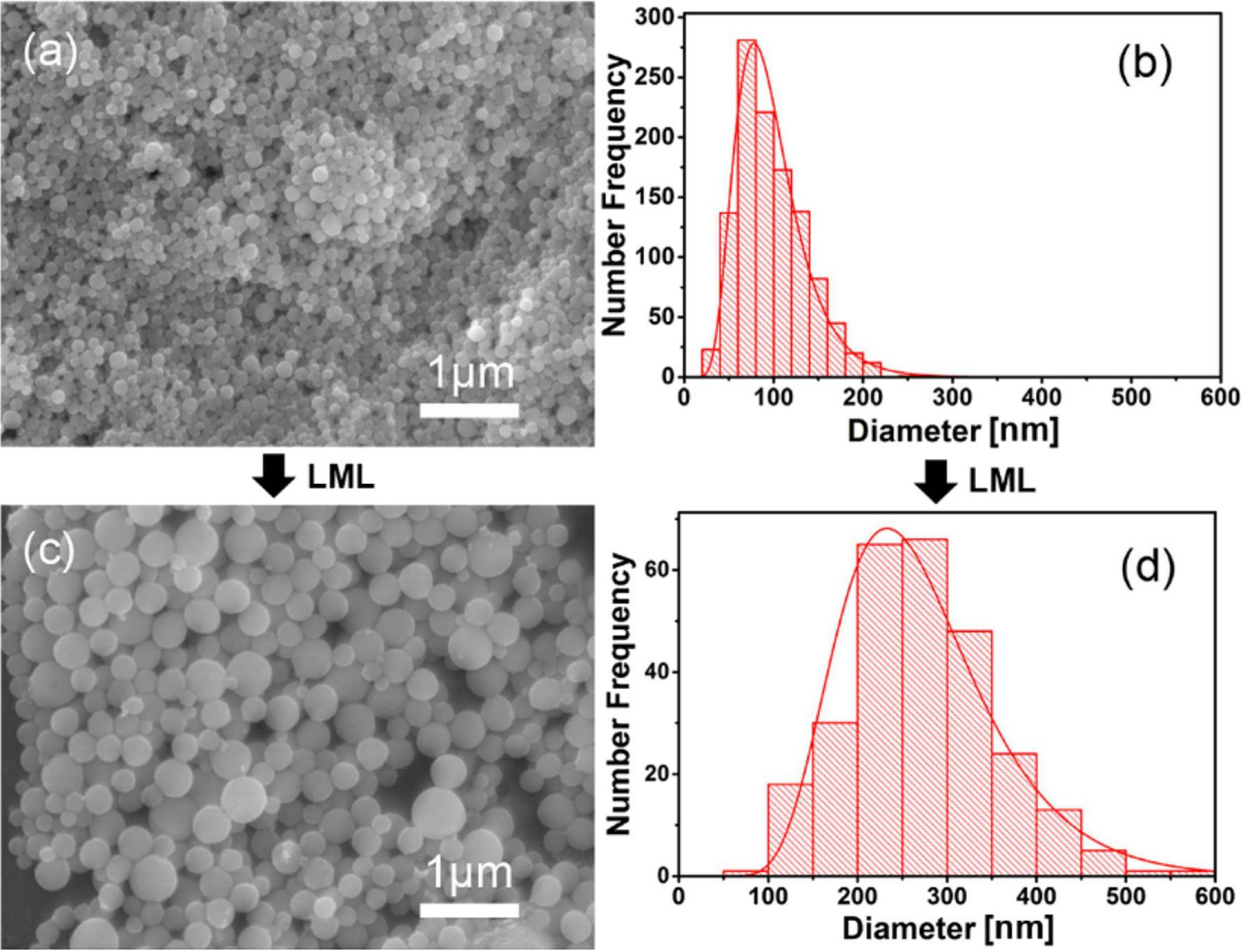
(a, b) SEM morphologies and size distribution of Ge nanoparticles obtained by laser ablation in water (c, d) SEM morphologies and size distribution of Ge submicrometer spheres synthesized by post laser irradiation of the LAL-synthesized particles. Figure 5 was reproduced from [53], (© 2017 D. Zhang et al., published by Springer Nature, distributed under the terms of the Creative Commons Attribution 4.0 International License, https://creativecommons.org/licenses/by/4.0).

A novel approach to synthesizing submicrometer spherical particles (SMSPs) involves exposing colloidal NPs dispersed in a liquid medium to pulsed laser irradiation. This technique is known as pulsed laser melting in liquids (PLML) [6,41–42]. PLML involves heating and melting of raw NPs with unfocused laser pulses, leading to their aggregation and the formation of submicrometer spherical particles as the resulting droplets are quenched by the surrounding liquid. This process is influenced by the size-dependent optical absorption efficiency and heat capacity of the NPs [43–44]. Utilizing this method, submicrometer spherical particles can be fabricated from a variety of materials, including metals [45–46], oxides [47], semiconductors [48], and even carbides [41,49]. When compared to other particle fabrication processes, material versatility of this technique stands out. Further, the SMSPs obtained by PLML are unique in a way that they are spherical, mechanically very strong [50], and usually observed to be single crystalline [51]. A review article by Ishikawa et al., published in the year 2023, provides a comprehensive overview of the historical background, different materials, mechanisms, parameters and applications of PLML [52]. In a study where small germanium (Ge) NPs (≈80 nm) synthesized by LAL were subjected to picosecond laser irradiation in the laser melting in liquids (LML) setup, the particles grew into significantly larger SMSPs (≈230 nm) after multiple laser passages [53]. Figure 5 presents SEM images and corresponding size distributions of Ge-based nanostructures. This demonstrates the capability of LML to transform smaller NPs into larger spherical structures, confirming the process’s potential for tuning particle size through laser irradiation.

**Figure 6:**
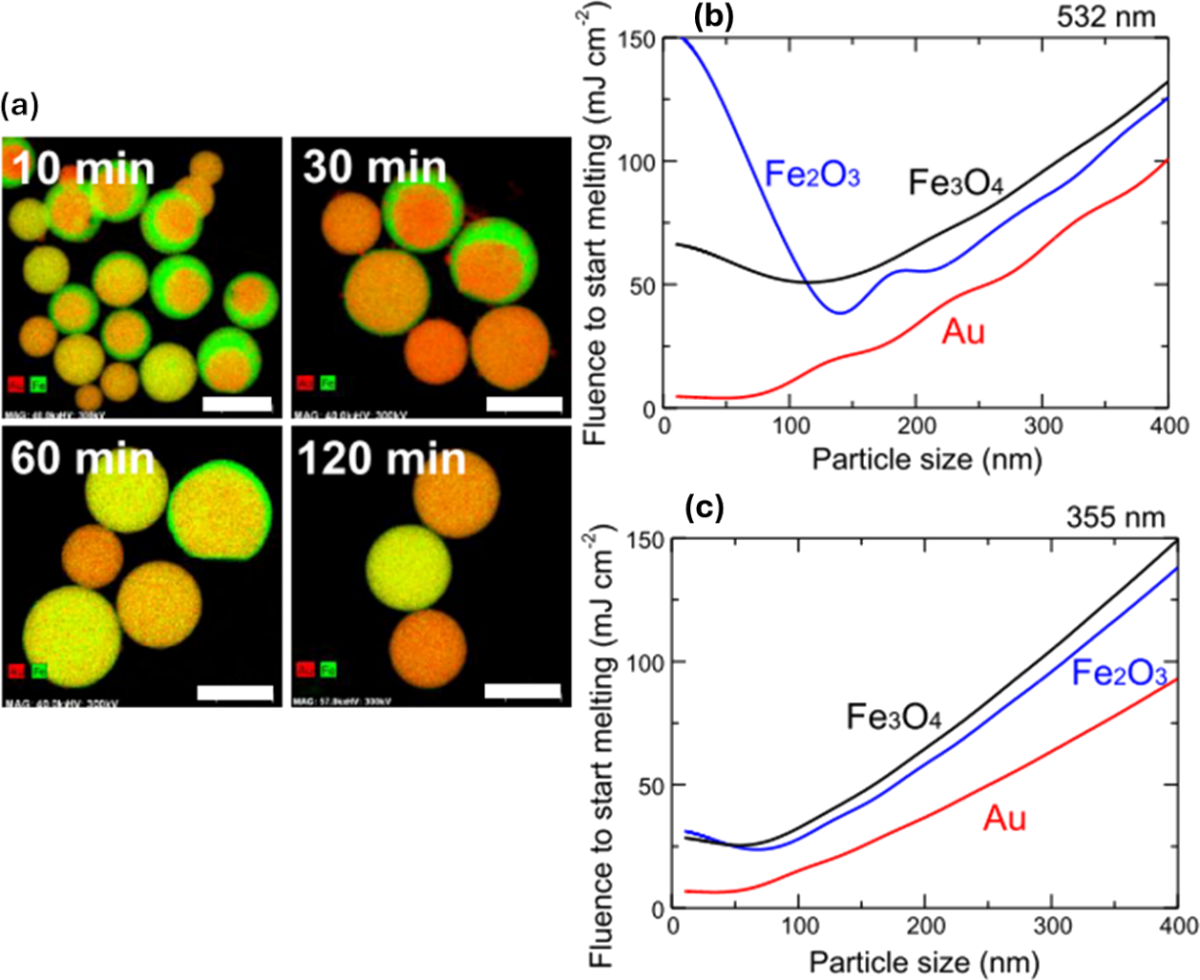
(a) EDS mapping images of particles obtained by different laser irradiation times from the Au raw particle solution. Particle size dependence of laser fluence required to melt a single particle of Au, Fe_3_O_4_, and Fe_2_O_3_ calculated based on Mie theory and adiabatic assumption (b) 532 nm. (c) 355 nm. Figure 6 was reproduced from [61] (© 2019 H. Fuse et al., published by MDPI, distributed under the terms of the Creative Commons Attribution 4.0 International License, https://creativecommons.org/licenses/by/4.0).

The productivity of SMSPs synthesized by PLML increases with high-power laser irradiation because the mass of melted particles is proportional to the total energy input by the laser [54]. Using picosecond laser irradiation at a low fluence is an energy-efficient method for synthesizing SMSPs by PLML compared to nanosecond laser irradiation [55]. If the thermal diffusion length in particles during ultrafast laser heating is smaller than the particle size, inhomogeneous heating occurs, leading to nanoparticle formation as a byproduct [55–56]. Therefore, a method called burst mode has been explained to control particle heating and suppress byproduct formation through partial evaporation as shown in Figure 6 [54,57–60]. The results showed that the collection rate of these particles increased as the number of burst pulses per train increased, with high burst pulses leading to minimal changes in particle shape. Ultimately, burst pulse irradiation is an effective method for reducing partial evaporation and controlling byproduct formation allowing better thermal management during nanoparticle synthesis.

In PLML, the time between consecutive laser pulses must allow for particle solidification and cooling on a microsecond timescale to form spherical and crystalline submicrometer particles [43,53,62]. Laser fluence is a key factor influencing the particle size [61]. Figure 5b,c depicts this dependency studies of laser fluence required to melt a single particle of Au, Fe_3_O_4_, and Fe_2_O_3_ using different wavelengths of laser (355 and 532 nm). Longer laser irradiation times lead to larger particle sizes, with initially inhomogeneous particles gradually becoming more homogeneous over time. The advantages of submicrometer particles produced by PLML over NPs synthesized by conventional PLAL, can be explained using Au–Fe systems [61], where the bulk thermodynamic contribution leads to an Au-enriched core and Fe-rich surface [46]. In contrast, conventional PLAL methods are dominated by surface energy, resulting in a Fe core–Au shell structure. The study identifies the limitations of lower fluence, inhomogeneous heating, and insufficient particle association during laser irradiation, and clarifies the mechanism behind PLML. For single-component materials, spherical particles are easily formed through laser melting, while two-component alloys, like Au–Fe, show distinct morphologies due to differences in heating behavior and optical absorption. Additionally, reactive fabrication of SMSPs with compositions differing from raw materials, such as B_4_C from B or Cu from CuO, is also achievable through laser irradiation.

#### 1.4 Laser irradiation in liquids

Laser irradiation in liquids (LIL) has become a multipurpose and effective technique for the synthesis and modification of NPs. This method involves the use of laser beams to irradiate colloidal solutions, enabling the generation of NPs with controlled sizes and shapes. One of the early significant studies in this field was conducted by Pyatenko et al. in 2007 [63], who demonstrated that spherical Ag NPs of various sizes could be synthesized through one-step or multistep processes, involving soft laser treatment of the final product or intermediate colloids. They defined “soft laser fluence” as the maximum laser intensity that could heat and melt the particles without causing evaporation, thus preserving their size. This approach was based on a particle heating–melting–evaporation model, which was experimentally validated. In 2010, as research on pulsed laser irradiation progressed, Koshizaki’s group explored the potential of non-focused laser beams with moderate fluence, lower than the ablation threshold, to irradiate colloidal NPs [42,48–49,64–67]. Their findings were groundbreaking as they observed the fusion of NPs into submicrometer-sized particles (SMPs), a method that had not previously been controllable or reported for increasing particle size. This research highlighted the ability of pulsed laser irradiation to induce nanoparticle fusion and expand particle size through a relatively simple process, further advancing the field of laser-based nanoparticle synthesis.

**Figure 7:**
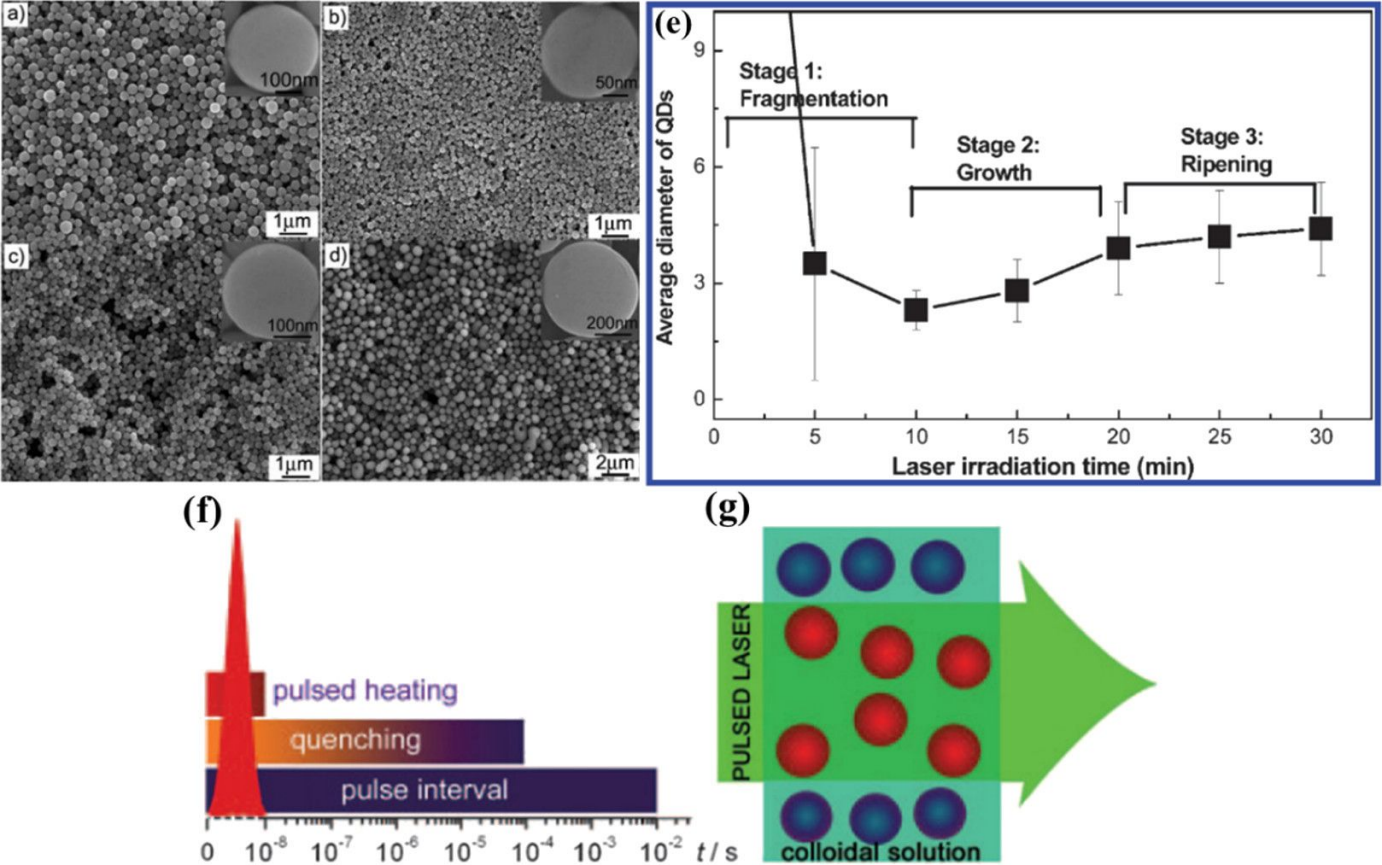
FESEM images of the submicrometer spheres obtained by the modest laser irradiation of commercial (a) ZnO, (b) WO_3_, (c) Cu, and (d) Fe NPs. Insets: magnified SEM images. (e) Evolution of average size of QDs with laser irradiation. The sharp decrease in the first stage is attributed to the rapid collapse and transformation into plasma, and the subsequent slight increase in the second stage corresponds to the growth of QDs. The selective pulsed heating involved in pulsed laser irradiation of colloidal nanoparticles. (f) Temporal discontinuity: pulsed heating and subsequent quenching, and (g) spatial discontinuity: only the particles are heated, not the solvent. Figure 7a–d,f,g was reproduced from [42], H. Wang et al., “Selective Pulsed Heating for the Synthesis of Semiconductor and Metal Submicrometer Spheres”, *Angewandte Chemie - International Edition* with permission from John Wiley and Sons. Copyright © 2010 WILEY‐VCH Verlag GmbH & Co. KGaA, Weinheim. This content is not subject to CC BY 4.0. Figure 7e was adapted with permission from [34], Copyright 2011 American Chemical Society. This content is not subject to CC BY 4.0.

The first report on the fabrication of ZnO quantum dots (QDs) through laser irradiation of ZnO hollow nanospheres in a liquid medium highlighted a rapid, simple process conducted at room temperature and normal atmospheric pressure [34]. A laser fragmentation mechanism was proposed, based on the interaction between the laser and the nanospheres, which generates plasma (Figure 7e). Most LIL processes can be classified as either fragmentation or melting, depending on the type of final product obtained. These mechanisms play a crucial role in determining the characteristics of the final products, whether they be spherical NPs, QDs, or other nanostructures, depending on the specific goals of the synthesis. NPs exhibit distinct mechanical and physicochemical properties that are influenced by factors such as size, surface-to-volume ratio, crystalline structure, composition, oxidation state, shape, and defects [68]. To synthesize NPs, colloids suspended in transparent liquids are irradiated with short or ultra-short laser pulses, which result in selective absorption by the NPs. This occurs through the interaction of the electromagnetic waves with the electrons of the NPs, causing collective motion in metallic particles or perturbations in the electronic orbitals of non-metallic NPs [42–43]. In the case of non-metals, nanosecond laser pulses transfer energy from electrons to phonons, leading to thermal equilibration between the electrons and the lattice. The absorbed laser energy then heats the NPs, causing melting, evaporation, reshaping, or alloying, particularly in mixed colloidal metal suspensions. Modest laser irradiation has proven effective for synthesizing submicrometer spheres and is suitable for a variety of metal and semiconductor NPs, including Ag, Au, Cu, Fe, Ni, Fe_2_O_3_, Fe_3_O_4_, Co_3_O_4_, NiO, TiO_2_, WO_3_, and ZnO, which all exhibit optical absorption at 355 or 532 nm (using a Nd:YAG laser).

Pulsed laser irradiation of colloidal NPs differs from LAL primarily in that PLIL uses an unfocused laser beam and source NPs, ensuring a more homogeneous and controlled reaction environment [69]. One significant drawback of LAL is its very low productivity, typically only several milligrams per hour [70–71]. However, research has shown that transforming NPs into submicrometer spheres can be accomplished in a shorter time frame, leading to a productivity increase of about two orders of magnitude compared to LAL. This improvement is due to the reduction in particle size from bulk material to NPs, which enhances the laser absorption efficiency. PLIL offers several advantages over traditional LAL, including milder reaction conditions, greater flexibility in size and phase control, and significantly higher productivity, making it more suitable for industrial-scale applications [42]. Additionally, selective pulsed heating in PLIL is distinct from conventional heating as it is both temporally and spatially discontinuous and can achieve ultrahigh temperatures even in solvents with low boiling points. The selective heating of NPs within a colloidal medium by a pulsed laser facilitates the melting or even vaporization of the particles. This occurs despite the low boiling point of the surrounding solvent, owing to the efficient light absorption capability of the NPs and their limited thermal transfer to the solvent. [35,63,72–74].

**Figure 8:**
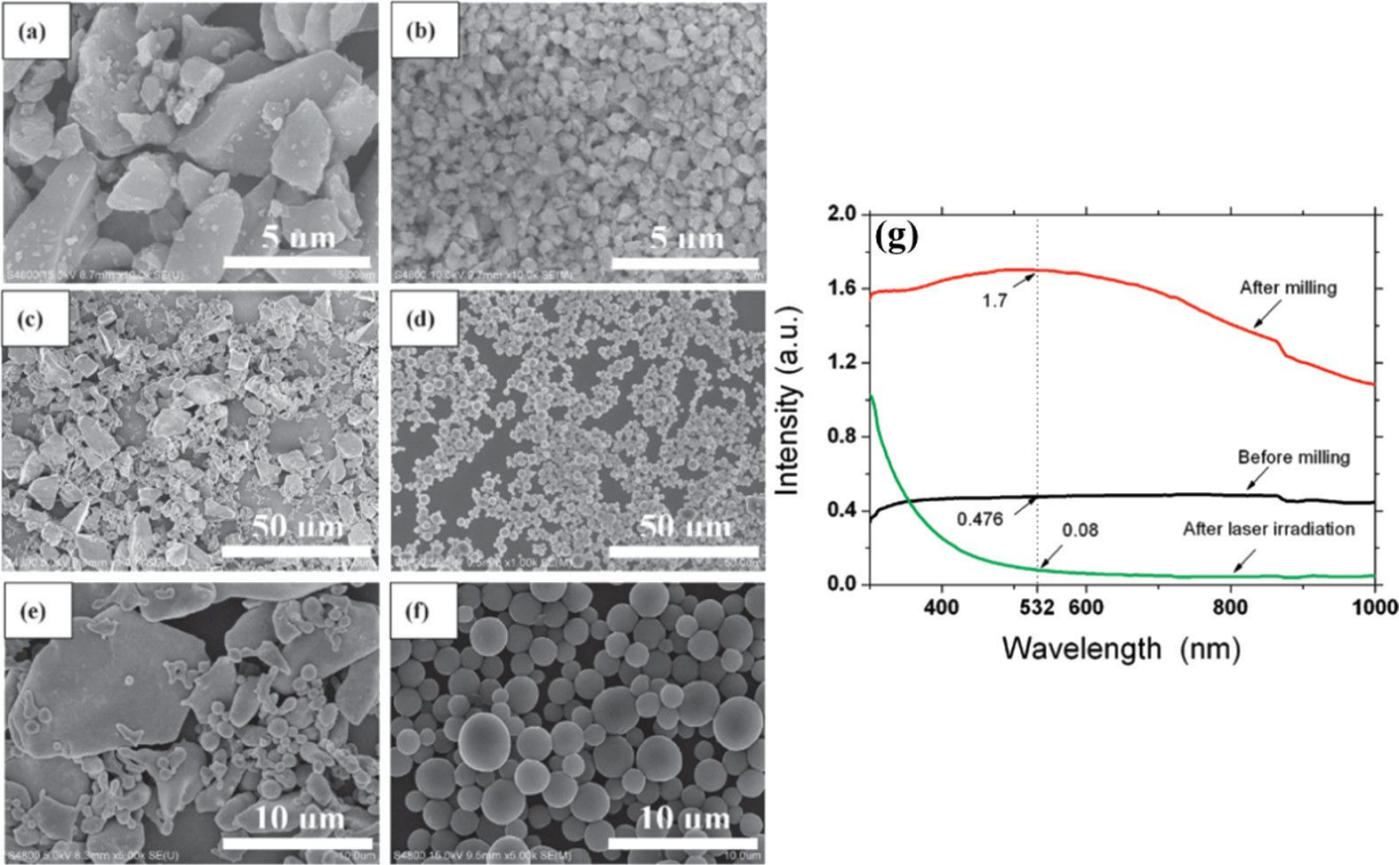
SEM of silicon particles (a, b) before and (c, f) after laser irradiation (460 mJ/pulse cm^2^, 30 min). Images (a), (c), and (e) are the morphologies without milling and (b), (d), and (f) are those with milling. Images (e) and (f) are magnified images of (c) and (d), respectively. (g) Absorption of silicon particles in ethanol before and after milling. Figure 8 was reprinted with permission from [48], Copyright 2011 American Chemical Society. This content is not subject to CC BY 4.0.

LIL also leads to phase transitions and morphological changes (Figure 7a–d) depending on the laser fluence and irradiation time [42]. Schematic illustrations of the pulsed laser heating mechanism are presented in Figure 7f,g. Factors such as laser fluence, irradiation time, and solvent properties, such as dielectric constant and thermal conductivity, must be carefully controlled to optimize nanoparticle formation [49,68–69,75]. Other factors like the milling effect have been noted to impact particle formation and morphology (Figure 8), as evidenced by studies such as the generation of Si particles in ethanol [48]. Milling large particles into smaller ones improves their absorption properties and facilitates the formation of spherical NPs by increasing surface area and inducing fracture planes. One of the few publications on the formation mechanism of gold SMPs [45] clearly depicts the laser irradiation mechanism and how the process of agglomeration affects the particle formation. Laser irradiation causes a redshift in the localized plasmon band, indicating an increase in particle size [76].

Laser irradiation offers a clean, efficient, and useful method for synthesizing various nanomaterials, including metals, semiconductors, metal oxides, and bimetallic NPs [46,77–82]. The process allows for the alloying of NPs, such as AuCo [77], through melting, diffusion, and rapid cooling, resulting in SMSPs. It also enables the creation of metastable compositions and defect-rich particles. Recent advancements in focused and collimated irradiation techniques have improved alloying efficiency and particle size control, further enhancing the potential of laser irradiation for nanomaterial synthesis.

Laser processing techniques such as ablation, fragmentation, and melting in liquids have shown tremendous potential in the fabrication and modification of nanomaterials for a wide range of applications. These methods offer distinct advantages, including the ability to produce nanomaterials with precise control over size, shape, and composition, all under environmentally friendly conditions. The ability to generate complex nanostructures, from NPs to hybrid nanomaterials, is particularly valuable for advancing fields like optics, bioelectronics, and catalysis. However, challenges remain, such as the need for improved productivity, narrower size distributions, and better understanding of the dynamic processes involved in laser interactions with liquid media.

#### 1.5 Laser-induced defect engineering in liquids

Laser-based techniques have unlocked new possibilities for the intentional introduction and tailoring of defects within liquid media [83]. These laser-driven defect engineering approaches enable precise control over the structural, and chemical properties of liquids, making them invaluable for a wide range of applications, from energy storage and conversion to chemical processing and material synthesis [84–85]. One of the key mechanisms underlying laser-induced defect engineering in liquids (LDL) is the rapid and localized energy deposition facilitated by laser irradiation. The high-intensity, coherent light can selectively and rapidly heat target liquid regions, triggering various physical and chemical processes that lead to the formation and stabilization of desired defect structures. These processes can include thermal decomposition, phase transformations, chemical reactions, and the introduction of impurities or dopants [86–87]. For example, laser irradiation has been utilized to precisely and controllably introduce a variety of structural defects, such as vacancies and interstitials, within electrode materials for electrochemical energy storage and conversion applications [85–86,88]. By carefully tuning the laser parameters, researchers have been able to selectively create and stabilize the desired type and distribution of defects within the electrode materials, leading to improvements in energy density, power density, and cycling stability of electrochemical energy storage devices [88–91]. Furthermore, LDL has emerged as a powerful approach to fabricate advanced heterostructures and integrated electrode architectures, which are challenging to achieve through conventional synthesis methods in the development of high-performance energy storage and conversion devices [85,88,92–96].

The experimental setup for LDL typically involves a tightly focused laser beam interacting with a liquid precursor or solution. This precise control over the laser parameters, including wavelength, pulse duration, and energy density, allows researchers to carefully tailor the nature and distribution of the introduced defects within the liquid medium [86,91,93,95]. For instance, studies have reported that by meticulously adjusting these laser characteristics, they were able to selectively create and stabilize specific types of defects, such as vacancies and interstitials, within electrode materials [97]. Unlike methods that focus on bulk transformations, this technique enables the targeted introduction and fine-tuning of specific defects, unlocking unprecedented opportunities for advanced material design and performance optimization [86]. By carefully adjusting the laser parameters, the spatial distribution, and concentration, characteristics of defects can be tailored with unparalleled precision [95,97–98]. The strategic placement and tuning of defects within these materials can significantly enhance their electrochemical performance, improving charge transport kinetics, increasing active surface area for redox reactions, and facilitating the intercalation and deintercalation of ions during energy storage and conversion processes [90]. Thus, LDL represents a transformative approach that far surpasses traditional laser-based techniques in its ability to precisely manipulate the structural, chemical, and functional attributes of materials at the nano/microscale. Table 1 compares various laser-based techniques for synthesizing nanocolloids, highlighting their mechanisms, suitable material types, particle characteristics, and relevance to applications such as photovoltaics, sensors, and catalysis. This table is a summary based on the discussion and cited literature in Section 1.

**Table 1:** Comparison of laser-based nanocolloid synthesis techniques for various applications.

Technique	Typical particle size (nm)	Size distribution	Shape control	Surface purity	Scalability
	Mechanism				
	Material types				
	Film formability				
	Device relevance				

laser ablation in liquids	5–100+	broad to bimodal	moderate	very high	moderate
	ablation of solid target submerged in liquid → plasma plume condensation				
	metals, semiconductors, oxides, carbides, alloys				
	requires post-processing, e.g., centrifugation, LFL				
	photodetectors, photovoltaics, SERS, photocatalysis				

laser fragmentation in liquids	<5–20	very narrow	high	high	moderate to high
	fragmentation of larger NPs by short-pulse laser → smaller, monodisperse particles				
	metals, oxides, semiconductors, perovskites				
	excellent; produces uniform colloids ideal for films				
	SERS, UV photodetectors, photocatalysis				

laser melting in liquids	100–1000 (SMSPs)	moderate	high	high	high
	melting/aggregation of raw colloids under laser → submicrometer spheres				
	metals, oxides, semiconductors, carbides				
	excellent; forms dense, durable films				
	PV, photodetectors, load-bearing sensors				

laser irradiation in liquids	20–200	moderate	moderate	high	high
	modification or alloying of colloidal particles under laser irradiation				
	hybrid structures, metal alloys, bimetallics				
	excellent; post-reshaping or tuning				
	LEDs, hybrid PV, magneto-plasmonics				

laser defect engineering in liquid	varies; not size-specific	not size-focused	not shape-focused	high	moderate
	introduction of defects/doping under controlled laser irradiation				
	electrode, energy storage, semiconductor materials				
	indirect; alters electronic properties				
	electrochemical devices, sensors, HER/OER				

### 2 Nanocolloids by laser processing in liquids to thin film fabrication

**Figure 9:**
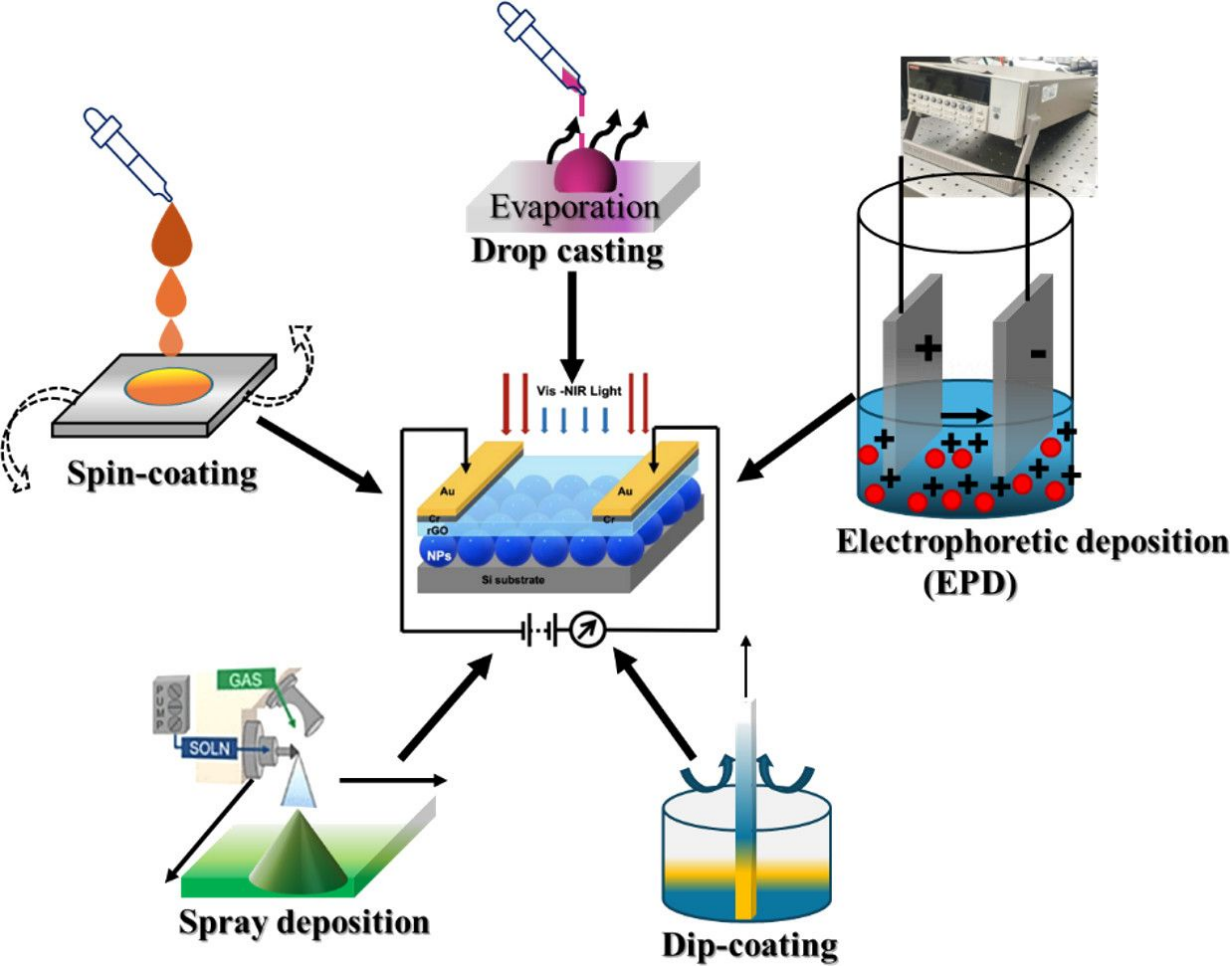
General schematics of the different techniques of thin film fabrication using nanocolloids synthesized by LPL. The photodetector device figure used at the center was reproduced with permission from [99] (© 2021 N. S. Rohizat et al., published by Springer Nature, distributed under the terms of the Creative Commons Attribution 4.0 International License, https://creativecommons.org/licenses/by/4.0).

For device applications, nanocolloids for thin film deposition play a crucial role in fabrication of devices and enhancing their performance. Pulsed laser processing in liquids offers a unique advantage by producing surfactant-free nanocolloids, which can be directly used for the fabrication of thin film devices such as photodiodes, photovoltaics (PV), photocatalysts, surface-enhanced Raman spectroscopy (SERS) sensors, electrochemical sensors, and HER/OER devices. These films exhibit unique morphologies and properties due to the controlled nanoparticle size and dispersion. Additionally, these nanocolloids enable facile, low-temperature, single-step film fabrication under standard laboratory conditions, making it an efficient and versatile method for developing advanced functional materials. This section of the discussion focuses on the fabrication of thin films using laser-processed nanocolloids by various techniques such as electrophoretic deposition (EPD), spin-dip coating, spin coating, drop-casting, and spray deposition. The choice of thin film fabrication method significantly influences the morphology and properties of the resulting films. A brief overview is provided here, as comprehensive reports and tutorial reviews on each of these techniques have already been published. A general schematic of different techniques of thin film fabrication using nanocolloids synthesized by LPL is given in Figure 9.

#### 2.1 Spin coating

**Figure 10:**
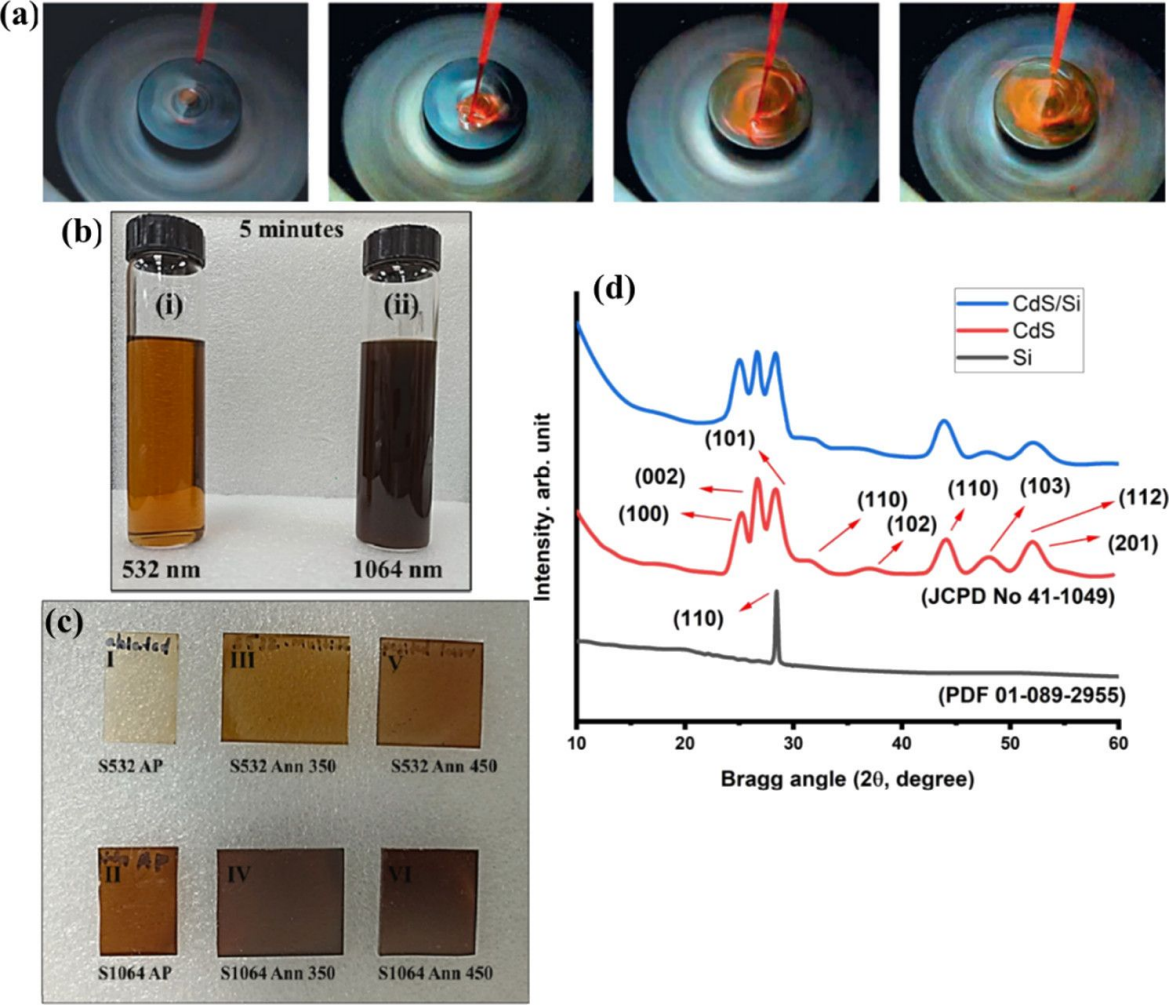
(a) Photograph of a typical spin-coating operation and high-speed images showing application of a solution of MEHPPV to a rotating substrate and film formation. The timing of the images (from left to right) after impact of the first drop is: *t* = 17, 100, 137, and 180 ms. (b) Photos of the SnS nanocolloids prepared by PLAL using laser of wavelength (i) 532 nm, (ii) 1064 nm and (c) their thin films by spin coating (I, II) SnS as-prepared (AP), (III, IV) annealed at 350 °C, (V, VI) annealed at 450 °C, (d) XRD diffractogram of CdS nanoropes, Si wafer p-type, and CdS film on Si (111) substrate prepared by spin-coating. Figure 10a was reprinted from [112], *Solar Energy Materials and Solar Cells*, vol. 93, by F. C. Krebs, “Fabrication and processing of polymer solar cells: A review of printing and coating techniques”, pages 394–412, Copyright (2009), with permission from Elsevier. This content is not subject to CC BY 4.0. Figure 10b,c was reprinted from [104], *Colloids and Surfaces A: Physicochemical and Engineering Aspects*, vol. 639, by A. P. Sreekala; B. Krishnan; R. F. C. Pelaes; D. A. Avellaneda; M. I. M. Palma; S. Shaji, “Tin sulfide thin films by spin coating of laser ablated nanocolloids for UV–Vis–NIR photodetection”, article no. 128382, Copyright (2022), with permission from Elsevier. This content is not subject to CC BY 4.0. Figure 10d was reproduced from [109] (© 2023 F. H. Alkallas et al., published by MDPI, distributed under the terms of the Creative Commons Attribution 4.0 International License, https://creativecommons.org/licenses/by/4.0).

The pioneering analysis of spin coating dates back over fifty years to Emslie et al., who first studied the spreading of a thin axisymmetric film of Newtonian fluid on a rotating substrate [100]. In 1989, the method was used to deposit photoresist films for microelectronic manufacturing, where understanding the interaction between liquid flow and mass transfer during the process became crucial as device sizes decreased. A one-dimensional model of spin coating was developed to describe film thinning due to both convective outflow and solvent evaporation, highlighting the formation of a solid “skin” at the surface, which could lead to defects if convective flow ceases too late [101]. By 2009, spin coating had become the dominant technique for producing uniform thin films of photosensitive organic materials, with thicknesses ranging from micrometers to nanometers. A mathematical model was derived to explain the dominant mechanisms in film formation, considering factors such as evaporation and shear stress, and extending the analysis to non-Newtonian fluids [102]. Despite its widespread use, the process faces challenges such as thin film defects, including comet marks, striation, chuck marks, environmental sensitivity, and edge effects. Remedies for these issues have been explained in a comprehensive review of the spin coating technique that emphasized parameters controlling the process, including spin speed, time, and acceleration [103]. The spin coating method is a straightforward, simple, and room-temperature technique for producing thin films. Uniform coatings are produced by evenly casting a solution of the required substance in a solvent (“ink”) across the substrate’s surface while it is spinning [104]. Figure 10a shows a photograph of a typical spin-coating setup, along with high-speed images capturing the dynamics of and film formation on a rotating substrate.

Mono and bi-metallic Pt, Pd, and Pt_80_Pd_20_ NPs were synthesized using a Nd:YAG laser operating at λ = 1064 nm, a fluence of 5 J/cm^2^, and a repetition rate of 10 Hz. In order to synthesize Pt and Pd NPs, pure platinum and palladium metal plates were used as ablation targets. Metallic NPs synthesized through PLAL can be utilized not only for forming thin films of their own but also in combination with organic semiconductors via spin coating. Thin films of copper phthalocyanine (CuPc) doped with laser generated Au and Ag NPs were prepared using spin coating on glass substrates. Transmission electron microscopy analysis indicated spherical NPs having sizes in the range of 7–43 nm for Au NPs, and 12–50 nm for Ag NPs. The deposited films had good homogeneity, granular nature, and adhered well to the substrate. With just 50 μL of the solution of each sample spin-coated onto the substrate and dried afterwards, a uniform coating was obtained and energy-dispersive X-ray spectroscopy (EDX) also revealed compositional uniformity of Au and Ag throughout the CuPc films [105]. In order to fabricate a NP/graphene nanocomposite, drops of colloidal solutions from each samples were cast and spin-coated onto graphene substrates to obtain a “starry-sky” morphology, which can be used in fuel cells, sensors, catalysis, and electronic and optical devices [106].

Nanostructured films of SnS and its hybrids with Si and graphene prepared by employing laser fragmentation and ablation in different solvents were reported for photodetector applications. While SnS and SnS–Si NPs had spherical morphology [104,107], in SnS–graphene, the layered nature of graphene was visible and it helped in the optoelectronic performance as well [108]. The photographs of SnS nanocolloids synthesized via PLAL and the corresponding spin-coated thin films are shown in Figure 10b,c. CdS NPs generated by PLAL were deposited as a thin film photodetector by spin coating the NPs on p-type Si with an electrical resistance of 3–5 Ω·cm. A thickness of 300 nm was recorded using a laser interferometer. The CdS/Si photodetector demonstrated that the junction exhibited proper rectification behavior and suggested that the CdS NPs may be a good nanomaterial for optoelectronic applications [109]. Figure 10d shows the X-ray diffraction (XRD) measurements of CdS nanoropes. A comparison of spin coating and drop casting for film fabrication on graphene paper was made using Ni/NiO NPs synthesized by LAL. Rutherford backscattering spectrometry measurements revealed that the homogeneity of the NPs was better after spin coating than drop casting [110].

A functionalization study on SiC nanocrystals was made. SiC–polyvinyl alcohol (PVA) polymeric nanocomposites were manufactured by adding silicon carbide NPs prepared by PLAL to PVA and thin films of the composites were prepared by spin coating on glass substrates using 750 rpm for 10 s. FTIR analysis revealed that the functional groups and atomic bonding were involved in physical, rather than chemical, interactions, as no new bonds were formed or broken [111]. These results support the notion that NPs can be combined with various materials, including polymers, and are suitable for film fabrication using spin coating under optimal conditions.

#### 2.2 Drop casting

**Figure 11:**
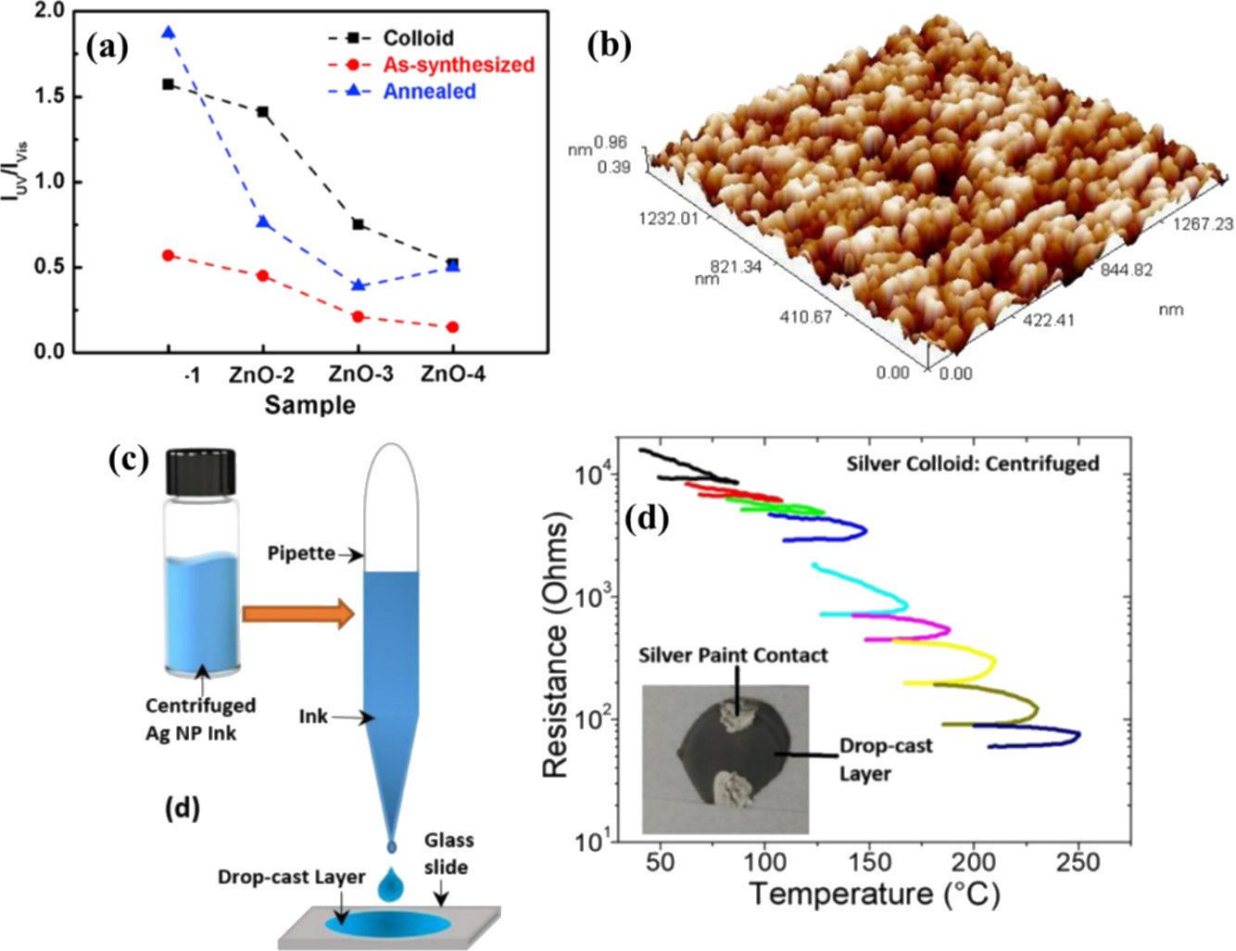
(a) Peak intensity ratio *I*_UV_/*I*_vis_ of UV near band edge emission to visible deep-level emission of ZnO colloidal solutions drop-cast onto Si wafers. (b) 3D AFM image of ZnO NPs drop-cast on glass substrate. (c) Schematic of centrifuged Ag NP ink drop-cast on a glass slide. (d) In situ resistance vs temperature during heat-treatment of the centrifuged Ag ink drop-cast layer. Figure 11a was reprinted from [115], *Materials Science in Semiconductor Processing*, vol. 109, by W. Chen; C. Yao; J. Gan; K. Jiang; Z. Hu; J. Lin; J. Sun; J. Wu, “ZnO colloids and ZnO nanoparticles synthesized by pulsed laser ablation of zinc powders in water”, article no. 104918, Copyright (2020), with permission from Elsevier. This content is not subject to CC BY 4.0. Figure 11b was reproduced from [119] (© 2019 B. Ali and Al-Mustansiriyah Journal of Science, published by Al-Mustansiriyah Journal of Science, distributed under the terms of the Creative Commons Attribution-NonCommercial 4.0 International License, https://creativecommons.org/licenses/by-nc/4.0/). This content is not subject to CC BY 4.0. Figure 11c,d was reprinted from [120], *Nano-Structures & Nano-Objects*, vol. 29, by É. McCarthy; S. P. Sreenilayam; O. Ronan; H. Ayub; R. McCann; L. McKeon; K. Fleischer; V. Nicolosi, “Silver nanocolloid generation using dynamic Laser Ablation Synthesis in Solution system and drop-casting”, article no. 100841, Copyright (2022), with permission from Elsevier. This content is not subject to CC BY 4.0.

Colloidal indium oxide (In_2_O_3_) NPs were synthesized using PLA of indium in water at room temperature. The thin film of In_2_O_3_ NPs was deposited on a n-type silicon by drop casting for heterojunction photodetectors, and the film surface coverage was improved by multiple layer depositions and condensation of colloidal suspension. Irregular particle shapes and sizes were visible in 3D atomic force microscopy (AFM) images with sizes ranging between 60 and 80 nm. The NPs agglomerated to form submicroparticles, which were clusters of many individual In_2_O_3_ NPs. A discrepancy was observed between the grain size measurements from XRD and AFM. This discrepancy arises because AFM directly visualizes the grains but does not account for structural defects, while XRD measurement determines the size of the defect-free volume [113]. A pellet of copper metal was utilized as a target for the production of CuO NPs in deionized water and the CuO NPs were then dropped on 3–5 Ω·cm p-type silicon substrate employing drop casting at low temperature to study its photodiode properties with a layer thickness of 400 nm. Packed flake-like aggregated structures with an average particle size of 77 nm were observed [114]. ZnO NPs were prepared by laser ablation of metallic zinc powders suspended in water, and thin films were fabricated by drop casting [115]. Figure 11a represents the UV–vis emission intensity ratio of ZnO colloidal solutions drop-cast onto Si wafers, indicating the optical quality and defect-related emission of the resulting films. ZnO NPs had irregular shapes in the 10–50 nm range. Also, ZnO and Au NPs with particle sizes of 31 and 20 nm, respectively, and Au:ZnO core–shell nanoparticles (CSNPs) with an average size of 53 nm were reported [116]. The Au:ZnO CSNPs were synthesized by laser ablation at various laser pulse energies of 400, 600, and 800 mJ. CSNPs obtained were drop-cast onto silicon wafers to form Au:ZnO CSNP thin films. Different sizes and aspect ratios of ZnO nanostructures generated in different liquid media by changing the laser parameters were reported. The colloids were drop-cast onto Si wafers for various studies [117]. NiO and ZnO nanostructured films were obtained on silicon and quartz substrates to examine their optical properties as well as their structural, topographical, and morphological features [118–119]. A 3D AFM image of ZnO NPs drop-cast on a glass substrate is shown in Figure 11b, revealing the surface morphology. Their photodetection and photovoltaic properties are discussed in the later sections of this review.

Research articles provide limited information on the thickness and control parameters related to this technique. However, Ag nanocolloids produced through LAL in the recirculation production mode were centrifuged and ultrasonicated to concentrate and re-suspend the settled Ag NPs. The resulting inks were drop-cast onto glass slides (Figure 11c), allowing the liquid to spread naturally and form elliptical or circular layers with diameters of 15–20 mm. The estimated liquid layer thickness was 1.6–2.8 mm. After the drop-casting process, about 5.4 mg of Ag NPs were deposited, resulting in a dried layer thickness of approximately 1.6–2.9 µm [120]. The corresponding in situ resistance measurement as a function of temperature during heat treatment of the Ag layer is presented in Figure 11d. In conclusion, drop-casting, due to its simplicity, involves merely dropping a colloidal solution onto a substrate, offering limited control over the deposition process. This method is widely utilized for forming thin films primarily for characterization purposes such as XRD, AFM, and photoluminescence (PL). However, apart from factors like strain of the substrate or temperature, if any applied to the substrate, no significant control over the film formation is achievable for conventional drop casting, making it less suitable for precision coating or device fabrication [121–122]. However, Eslamian et al. have reported substrate vibration-assisted drop casting, where drop casting combined with ultrasonic substrate vibration offers enhanced controllability. A small volume of precursor solution, characterized by low surface tension, is released from a capillary tube either by dripping with negligible initial velocity or by injection with an initial velocity. The droplet is then directed toward the substrate, where the ultrasonic vibration aids in controlling the deposition process [123]. However, this method has not yet been established for laser-processed colloids, making it an area open for further exploration.

#### 2.3 Doctor blade technique

**Figure 12:**
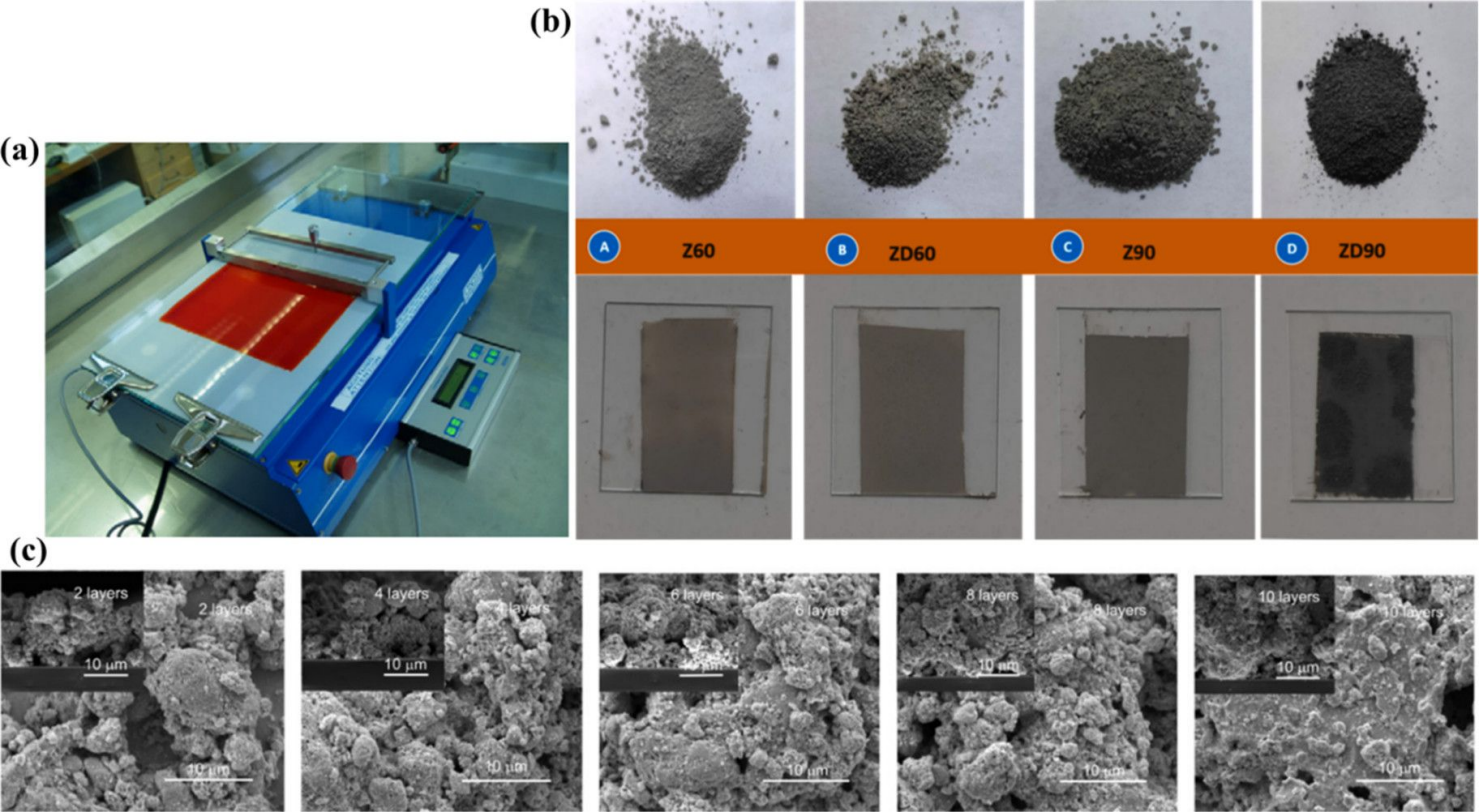
(a) Photograph showing the doctor blade technique. (b) Images of ZnO powders after 60 and 90 min of laser irradiation and their films prepared by doctor blade technique. (c) SEM images of the ZnO films deposited by doctor blade method and annealed at 400 °C. Figure 12a was reprinted from [112], *Solar Energy Materials and Solar Cells*, vol. 93, by F. C. Krebs, “Fabrication and processing of polymer solar cells: A review of printing and coating techniques”, pages 394–412, Copyright (2009) with permission from Elsevier. This content is not subject to CC BY 4.0. Figure 12b was reprinted from [125], *Applied Surface Science*, vol. 567, by S. S. Kanakkillam; B. Krishnan; S. S. Guzman; J. A. A. Martinez; D. A. Avellaneda; S. Shaji, “Defects rich nanostructured black zinc oxide formed by nanosecond pulsed laser irradiation in liquid”, article no. 150858, Copyright (2021), with permission from Elsevier. This content is not subject to CC BY 4.0. Figure 12c was reprinted from [126], Thin Solid Films, vol. 597, by C. Sima; C. Grigoriu; O. Toma; S. Antohe, “Study of dye sensitized solar cells based on ZnO photoelectrodes deposited by laser ablation and doctor blade methods”, pages 206–211, Copyright (2015), with permission from Elsevier. This content is not subject to CC BY 4.0.

The doctor blade technique is a method used to prepare layers with a precise and controlled thickness. One of its key advantages over other fabrication methods, such as spin coating, is the ability to minimize material loss, which can be as low as 5% [112]. The process involves positioning a sharp blade at a fixed distance from the substrate (Figure 12a). The final dry thickness of the coated film, *d* can be predicted using an empirical relationship based on the gap distance between the blade and the substrate, *g*, and *c*, the concentration of solid material in the ink in grams per cubic centimeter, and the density of the material, ρ in grams per cubic centimeter [112]:


[1] $$d=\frac{1}{2}\left(g\frac{c}{\rho }\right).$$

Thin films of ZnO and Ag/Au–ZnO hybrid nanomaterials were fabricated using the doctor blade method (Figure 12b), and the effects of annealing on their morphology, compositional homogeneity, and optical properties were investigated for photocatalytic activities [124–125]. The properties of these films are further discussed in the latter section of this article. A comparison of ZnO photoelectrodes obtained by laser ablation and doctor blade for dye-sensitized solar cell application was made. Regarding the crystalline structure, a noticeable difference between the films is the broadening of the diffraction peaks. Specifically, the films deposited using the doctor blade technique exhibit broader peaks compared to those deposited by laser ablation, where the peaks are narrower and sharper. SEM images of the surface and cross section of the films deposited by the doctor blade method, with varying ZnO layer thicknesses, reveal a noticeable difference in morphology. The films deposited by doctor blade exhibit higher porosity and distinct clustering compared to those made by laser ablation. This increased porosity in the doctor blade films is likely responsible for the better dye absorption observed. However, a disadvantage of the doctor blade method is the poor adhesion of the film to the substrate, especially as the film thickness exceeds a few micrometers, leading to the formation of cracks after solvent evaporation. Additionally, the doctor blade method does not offer precise control over film thickness [126]. The SEM images of the ZnO films deposited by doctor blade method are given in Figure 12c. Also, in Table 2, a comparison of thin film fabrication methods from selected literature using laser-generated nanocolloids, focusing on materials used, post treatments, film properties, and performance in applications, is given.

**Table 2:** Summary of selected reported studies on thin film fabrication from laser-synthesized nanocolloids and their key processing parameters.

Fabrication technique							

Material/ nanocolloid	Post treatment	Thickness	Homogeneity	Colloid details	Optical/ electronic properties	Device/ application	Ref.
	Performance highlights						

Spin coating							

SnS	annealing (350 °C, 450 °C)	not specified	high	PLAL in IPA and DMF	enhanced optoelectronic properties	photodetector	[104,108]
	graphene hybrid improves performance						
CdS on p-type Si	none	300 nm	uniform	PLAL synthesized	proper rectification behavior	photodetector	[109]
	high-purity CdS NPs show good rectifying junction						
CuPc:Au/Ag NPs	drying	thin; uniform	high	50 µL of colloid used	granular stacking morphology	optoelectronic devices	[105]
	stable and homogeneous coatings						
SiC-PVA composite	drying	not specified	uniform	PLAL SiC in PVA	FTIR confirms physical interaction	composite film for sensors	[111]
	spin speed: 750 rpm for 10 s						
Ni/NiO NPs	none	not specified	superior to drop-casting	PLAL synthesized	improved homogeneity	general thin films	[110]
	spin coating provides improved uniformity						

Drop casting							

In_2_O_3_	multiple layering	not specified	moderate	PLAL in water	agglomerated submicrometer clusters	photodetector	[113]
	layering enhances surface coverage						
CuO on p-Si	none	400 nm	moderate	PLAL in water	packed flake morphology	photodiode	[114]
	room temperature fabrication						
Ag NPs	heat-treated	1.6–2.9 µm	moderate	centrifuged and drop-cast	good electrical conductivity	conductive layer	[120]
	5.4 mg Ag NPs used						
Ni/NiO NPs	none	not specified	inferior to spin-coating	PLAL synthesized	granular, less uniform	general coating	[110]
	used for comparison with spin coating						

Spray deposition							

SnS	annealed	≈1 mm (from 1 L)	moderate	PLAL in IPA and DMF	porous surface; compact in DMF	photodetector	[127]
	annealing promotes compactness						
SnS:GO/ rGO	annealed	similar to SnS	uniform	SnS with GO/rGO	defect emissions via PL	photodetectors	[128]
	tunable by GO content						
MoO_3_	substrate heated (80–120 °C)	varies with time	uniform	PLAL synthesized	spherical grain distribution	photovoltaics	[129]
	controllable thickness and morphology						
CuSbS_2_	annealing	not specified	good coverage	laser-ablated CAS nanocolloids	1.5 eV bandgap	photovoltaics	[130]
	superstrate structure demonstrates diode characteristics						

Doctor blade							

ZnO	annealed (400°C)	micrometer-level	porous	PLAL synthesized	broader XRD peaks	dye-sensitized solar cell	[126]
	High dye absorption due to porosity						
Ag/Au-ZnO hybrids	annealed	not specified	moderate	PLAL ZnO and hybridized	improved photocatalysis	photocatalysis	[124–125]
	enhanced light absorption						

EPD							

Au NPs	none	20–41 µm	high	PLAL in DI water	plasmon resonance visible	SERS/ optoelectronics	[131–133]
	anodic and cathodic methods affect morphology						
CIGS	none	not specified	high	femtosecond PLAL in solvents	CIGS film formation	solar cell	[134–135]
	7.37% efficiency on Mo sheet						
quinacridone	none	70–200 nm	high	PLAL in water	NP size and voltage affect morphology	electrodes	[136–137]
	uniform organic thin films						

Dip coating							

Si NPs	drying	not specified	moderate	PLAL Si colloid	optical and structural variation	general electronics	[138]
	evaporation and drainage critical to uniformity						
CH_3_NH_3_PbI_3_ + PbS NPs	dipped post spin-coating	not specified	high	PbS PLAL + hybrid perovskite	improved absorber thickness	perovskite solar cell	[139]
	higher current density due to PbS						

#### 2.4 Spray deposition

**Figure 13:**
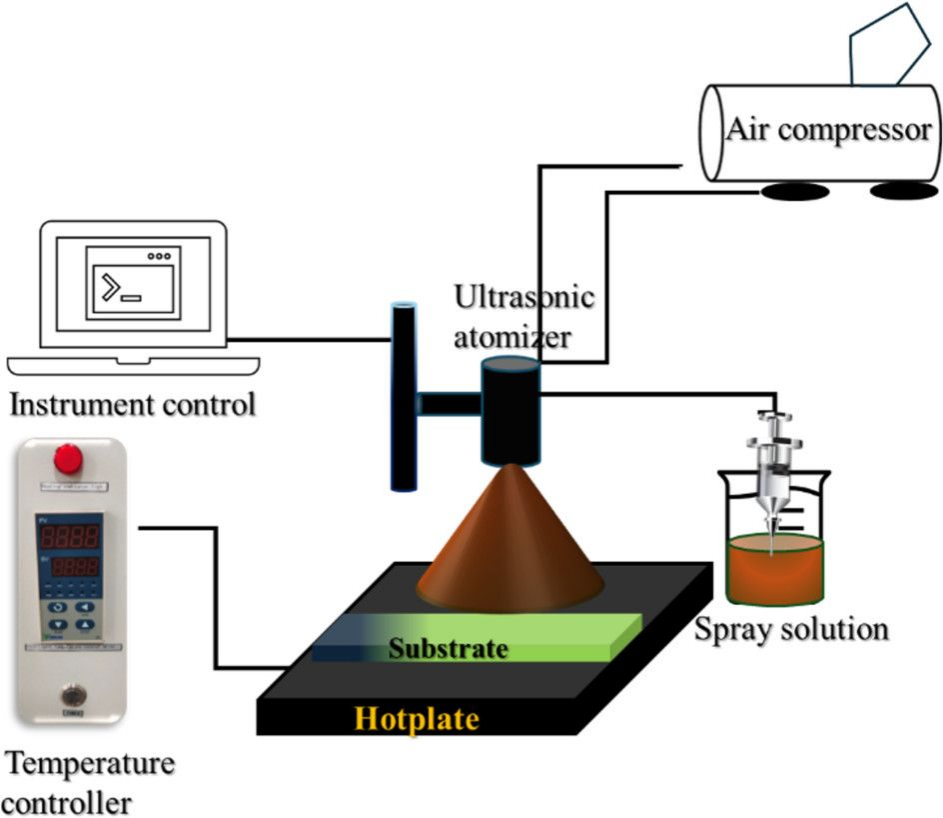
Schematic representation of an ultrasonic spray deposition setup.

The spray deposition technique creates uniform coatings on a substrate by directing an atomized stream of molten material droplets onto it [140]. A schematic representation of an ultrasonic spray deposition setup is provided in Figure 13. The thickness of the film is mainly determined by factors such as the distance between the substrate and the spray nozzle, substrate temperature, and precursor solution concentration. Additionally, the properties of the films depend on variables like the spray rate, ambient atmosphere, droplet size, carrier gas, and post-deposition cooling. One of the key advantages of this method is its ability to produce high-quality films without significant thickness limitations [141–142]. The synthesis of SnS NPs by pulsed laser ablation of SnS in isopropyl alcohol (IPA) and dimethyl formamide (DMF) using 532 nm output wavelengths was reported [127]. These nanocolloids were spray-deposited on different substrates kept on a temperature-controlled hotplate. The resulting films were stable and uniform with good adhesion to the substrates. The average thickness of films by spraying one liter of nanocolloid was around 1 mm as evaluated by SEM and profilometry. The morphology of as-deposited and annealed SnS thin films was analyzed using field-emission scanning electron microscopy (FESEM). Both sets of films exhibited a porous surface with layered structures. However, thin films made from SnS nanocolloids in DMF showed more compact NPs compared to those in IPA. This difference was attributed to the higher generation of NPs during the PLAL process when DMF was used and the spray conditions used to fabricate the nanocolloids into films, including substrate temperature and liquid medium properties (density and boiling point). Additionally, the annealing process contributed to the formation of more compact films.

A modification in this work was made by incorporating graphene oxide (GO) and reduced graphene oxide (rGO) into SnS to form nanocomposites. The only difference was the laser ablation wavelength (1064 nm) [128]. The deposition of SnS:GO nanocomposite thin film was achieved by adding different concentrations of GO to the SnS in IPA and DMF nanocolloids. The solution mixture was then sprayed onto heated substrates under the same spray conditions used for pristine SnS film depositions. The PL emission peak indicated the existence of defect centers in SnS and SnS:rGO thin films. The fabrication of SnS_2_ thin films via spray-deposition of laser-ablated SnS_2_ nanocolloids has been reported [143]. Copper antimony sulfide (CuSbS_2_, CAS) thin films have been deposited on glass, FTO, and Si substrates by spraying the laser-ablated CAS nanocolloid onto heated substrates [130]. Crystalline molybdenum oxide nanoparticles (MoO_3_ NPs) prepared by PLAL were employed to process uniform thin films of MoO_3_ in large areas with controllable thickness using ultrasonic spray deposition method for photovoltaic applications. Depositing thin films led to changes in morphology and particle size due to nanoparticle coalescence, which in turn altered the optical properties of the films. This resulted in a decrease in the optical bandgap (1.5 eV) compared to the NPs in colloidal form [129]. AFM images acquired from MoO_3_ NPs film confirmed the uniform distribution of the spherical particles onto the substrate. A variable grain size along with thickness and higher roughness is seen when longer times of ultrasonic deposition are used. This was verified using SEM, which showed the comparison between MoO_3_ NPs films produced at times of 40 and 60 s, as well as the thickness versus time of deposition with the substrate heated at 80 and 120 °C. A stable nanocolloid of cobalt oxide (Co_2_O_3_) was prepared in water by PLFL, and nanostructured cobalt oxide thin films were fabricated by spray deposition nanocolloid onto heated glass substrates [144].

#### 2.5 Electrophoretic deposition

**Figure 14:**
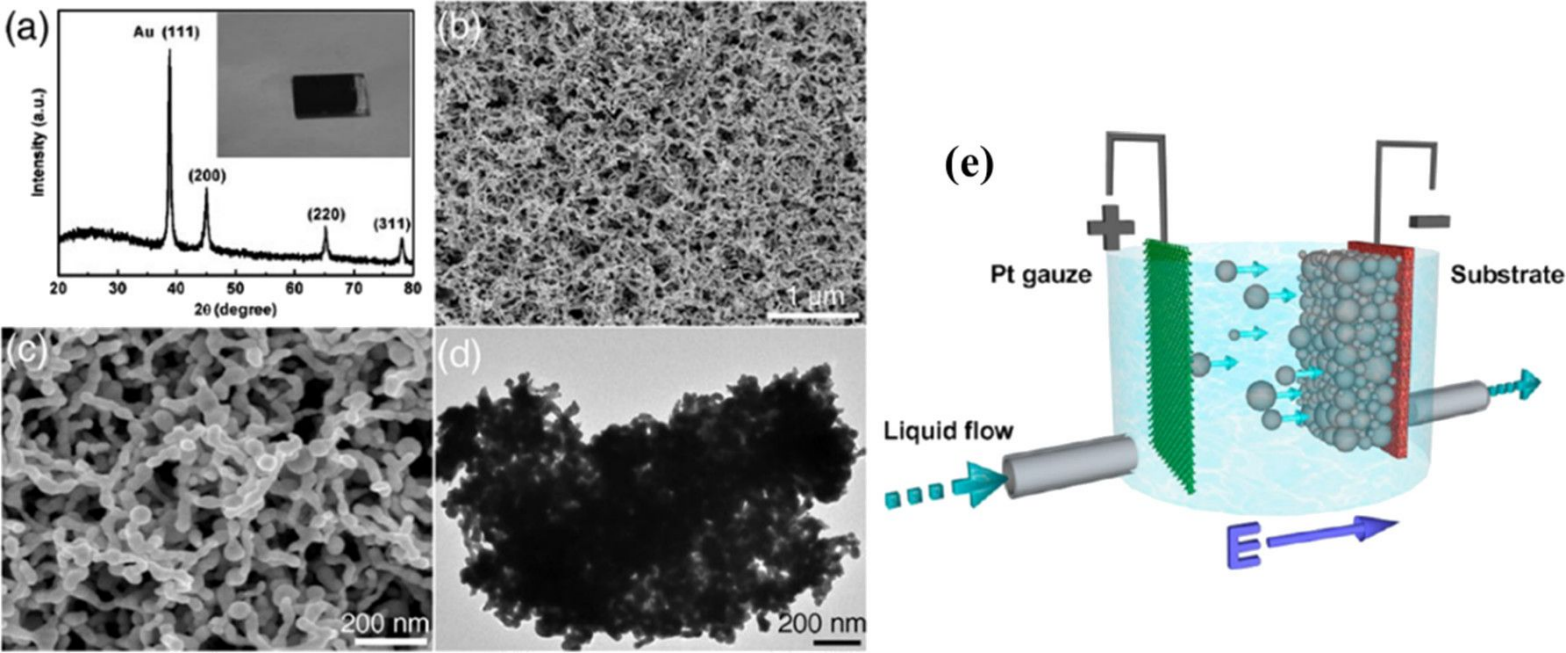
Structure and morphology of the Au sample prepared by EPD. (a) XRD result (b, c) FESEM images with different magnifications. (d) TEM image of the product scraped from the sample. (e) Schematic illustration of cathodic EPD of the nanoparticles in an electrical field. Figure 14a–d was used with permission of The Royal Society of Chemistry, from [131] (“Au nanochain-built 3D netlike porous films based on laser ablation in water and electrophoretic deposition” by H. He et al., *Chem. Commun.*, vol. 46, issue 38, © 2010); permission conveyed through Copyright Clearance Center, Inc. This content is not subject to CC BY 4.0. Figure 14e was adapted with permission from [134], Copyright 2012 American Chemical Society. This content is not subject to CC BY 4.0.

EPD is a process involving the electrokinetic mobility of charged particles in a suspension under an electric field to deposit them onto a substrate (Figure 14e). The key advantages of EPD include precise control over film thickness, uniform deposition, scalability for large-area coatings, and compatibility with a variety of materials achieved through careful control of several factors, such as particle surface charge, the electrical and fluid properties of the suspension, electrode material selection, and the current–voltage parameters. Additionally, it is a cost-effective, environmentally friendly method due to its low energy requirements and minimal use of solvents. EPD provides the notable benefit of film densification through the strong electric field near the substrate surface, eliminating the need for adhesives and binders [134,145–147]. Guo et al. reported a ligand-free method to synthesize colloidal metallic NPs using liquid-phase pulsed laser ablation, and used the NPs to fabricate Cu–In composite NPs and Cu(In,Ga)Se_2_ (CIGS) thin film solar cells through EPD [134–135]. They used cathodic EPD, where the substrate serves as the cathode, to prevent potential oxidation of the metallic NPs and substrate that could occur on the anode side. This was achieved by adding a low concentration of trivalent cations such as In or Ga into the colloid. The authors then synthesized colloidal metallic NPs of Cu−In and Cu−Ga alloys using an ultrashort femtosecond laser (1040 nm wavelength, 600 fs pulse duration, 10 μJ pulse energy in the megahertz range). The NPs were further analyzed regarding phase, composition, electrical surface charge, and charge modulation mechanisms. EPD of a Au colloidal solution prepared by laser ablation in different solvents has been reported [131]. Figure 14a–d presents the structural and morphological characterization of the Au sample fabricated by EPD. However, an example of anodic EPD was demonstrated by depositing Au NPs generated by LAL in deionized water onto graphene on a quartz surface at 30 V for 20 min and 1 h. A fairly uniform distribution of NPs was achieved, with surface densities ranging from approximately 15 to 40 nanoparticles per μm^2^. The hybrid materials exhibited plasmon resonance absorption of the Au NPs. Shorter EPD times maintained the properties of graphene, while longer deposition times resulted in the conversion of graphene to graphene oxide due to its electrochemical oxidation [132]. Another study found that the crystalline structure of electrophoretically deposited Au NPs thin films was not affected by deposition time or voltage. However, increasing the deposition time improved the film coverage. The thickness of the films increased with longer deposition times, but was more influenced by the EPD voltage. Increasing the deposition voltage in 10 min raised the roughness from 72 to 138 nm, while increasing the deposition time raised the roughness to 173 nm. So to achieve the desired thickness, roughness, and morphology, it is essential to optimize the EPD parameters accordingly [133].

The fundamental mechanisms and increasing applications of EPD of NPs have been widely studied [148]. Titanium diboride (TiB_2_) NPs were synthesized using five different laser fluences during laser ablation using a 1064 nm Nd:YAG laser in deionized water. The colloids were deposited on aluminium substrates with constant deposition time and voltage. XRD studies of these films confirmed that EPD is a reliable method for the deposition of suspended NPs in liquid media on solid substrates [149]. ZnO films obtained by EPD of ZnO NPs after PLA in deionized water and in aqueous cetyltrimethylammonium bromide (CTAB) solution on Pt substrates confirmed that the CTAB-involved assembly process led to the formation of elongated aggregates. It was also proposed that spindle-like aggregates were formed by surrounding the ZnO NPs with double layers of CTAB molecules [150]. A morphological modification using EPD was reported where a ZnO nanorod array prepared by chemical vapor deposition on Si substrate was used as the electrode and immersed in 20 mL Au colloidal solution, which was generated by laser ablation. Electrophoretic potential, Au nanoparticle size, and inter-rod spacing influence the decoration morphology, with EPD at low or high potentials hindering homogeneous decoration of the ZnO nanorods [151]. Too small or big inter-rod spacings relative to the size of Au colloids deposit Au NPs unevenly and heterogeneously. The authors suggest that the inter-rod spacing between the ZnO rods should be at least five times larger than the size of the Au NPs. Additionally, the strategy presented in this study [151] offered a new approach for the controllable modification of 1D semiconductor nanostructures and enhanced the understanding of the physical mechanisms underlying EPD. Si nanocrystals synthesized by laser fragmentation of Si powder in water and ethanol followed by film fabrication using EPD for photovoltaic application were reported, and the optimization of both laser-based surface engineering and EPD affects the performance of the inorganic-sensitized solar cells [39].

A pioneering work in the EPD of organic material synthesized by PLAL was reported. Colloids of Quinacridone (QA) NPs ranging from 25 to 120 nm were synthesized through laser ablation of a microcrystalline powder dispersed in water and subsequently deposited onto an indium tin oxide (ITO) electrode via EPD. The morphology of the films was influenced by the applied voltage. By varying the EPD time and colloid concentration, a thickness of 70–200 nm was achieved, and the resulting films exhibited uniformity across the entire substrate surface [136–137]. In conclusion, regardless of the material type, organic, inorganic, or semiconductor materials, EPD can effectively be utilized to obtain uniform films by employing the respective nanocolloids synthesized through laser techniques in liquids.

#### 2.6 Dip coating

Another less explored method of film fabrication from nanocolloids is dip coating. It remains a simple and traditional method for depositing uniform thin films onto substrates, particularly for small slabs and cylinders. The process relies on a delicate balance between viscous forces, capillary forces, and gravity to determine film thickness [152]. While the speed of substrate withdrawal can influence the thickness, achieving uniformity requires careful control overflow conditions, including liquid bath and gas overhead. As the process becomes more complex, especially with volatile solutes and rapid drying, it becomes more challenging to predict and control. Consequently, outside of product research and development labs, dip coating is less commonly used in precision coating manufacturing. A combination of spin coating and dip-coating was tried by Leal and colleagues [139,153]. CH_3_NH_3_PbI_3_ was first synthesized by spin-dip coating of the corresponding precursors, and a modification to improve the performance of the solar cell was done by dipping the films in PbS NPs synthesized by laser ablation. The SEM of CH_3_NH_3_PbI_3_:PbS films showed compact surfaces with elongated, well-defined cuboid particles formed after long dip-coating times [154]. The large crystals formed due to Ostwald ripening, where smaller grains with high surface energy grow at the expense of others, reducing the overall grain boundary energy [155]. Taheri et al. reported the optical and structural characteristics of Si NPs thin films prepared by PLAL and dip coating. The cleaned glass substrates were dipped in the colloidal solution, and pulled out with a speed of 40 mm/min vertically and dried afterwards [138]. Table 3 evaluates common film deposition techniques based on their advantages, limitations, film characteristics, and efficiency, providing a brief comparison for selecting appropriate methods in nanomaterial film fabrication. This table is a summary based on the discussion and cited literature in Section 2.

**Table 3:** A comparative study of deposition strategies for laser processed nanocolloids.

Technique				
Advantages			Disadvantages	
Film thickness	Homogeneity	Post-treatment	Colloid volume/conc. requirement	Particle wastage

spin coating				
- uniform films - room temperature - simple setup - compatible with polymers and composites			- sensitive to edge effects - defects like striations - material loss	
≈300 nm – a few µm	high	usually drying/annealing	≈50 µL per sample for uniform coating	moderate (due to overspray)

drop casting				
- very simple - no special equipment - suitable for characterization			- poor control over thickness and uniformity - prone to agglomeration	
400 nm to ≈2.9 µm (dried film)	low	drying/annealing	high; typically multiple drops or layers	high (significant material loss)

doctor blade				
- precise thickness control - minimal material loss (≈5%)			- poor adhesion at large thickness - cracking issues - hard to control homogeneity	
tens of micrometers, depends on gap	moderate	drying, annealing	requires concentrated inks (known *g*, *c*, ρ)	low

spray deposition				
- scalable - uniform coatings on large area - thickness control via time			- equipment complexity - depends on spray parameters and substrate temp	
~1 mm (1 L spray); also <1 µm possible	moderate–high	annealing improves compaction	variable (solution sprayed over time)	moderate–high (atomization loss)

electrophoretic deposition				
- high thickness control - uniform coatings - compatible with metals, semiconductors, organics			- requires charged particles - electrochemical issues at electrodes	
20–41 µm reported; (tunable)	high	drying/annealing	depends on field, time, voltage, colloid zeta potential	low (very efficient deposition)

dip coating				
- simple - suitable for small samples or complex shapes			- less uniform than spin-coating - hard to predict thickness - less used in industry	
not specified; tunable via withdrawal speed	moderate	drying	moderate volumes (immersion bath)	moderate (overflow/waste)

In conclusion, the various thin film fabrication techniques from nanocolloids are explained focusing on film growth, adhesion onto substrates, and advantages and disadvantages of each method. Post-processing of these thin films plays a critical role in enhancing the optical and electrical properties of the films. Techniques like annealing in air or vacuum are often required to improve film crystallinity, structural integrity, morphology, and functional performance.

The use of laser-processed nanocolloids to synthesize thin films offers significant potential for advancing various technological applications, including electronics, coatings, and sensors. While commonly used techniques which are explained in this section are effective, exploring alternative or hybrid methods could further enhance film quality and functionality. Techniques such as inkjet printing, roll-to-roll processing, laser-assisted chemical vapor deposition or integrating laser processing with atomic layer deposition, or molecular beam epitaxy could provide superior control over film structure and may offer scalable, cost-effective solutions, paving the way for innovative thin film technologies. These hybrid approaches may offer synergistic benefits, enabling the production of thin films with improved uniformity, mechanical strength, and functionality.

### 3 Devices and applications

**Figure 15:**
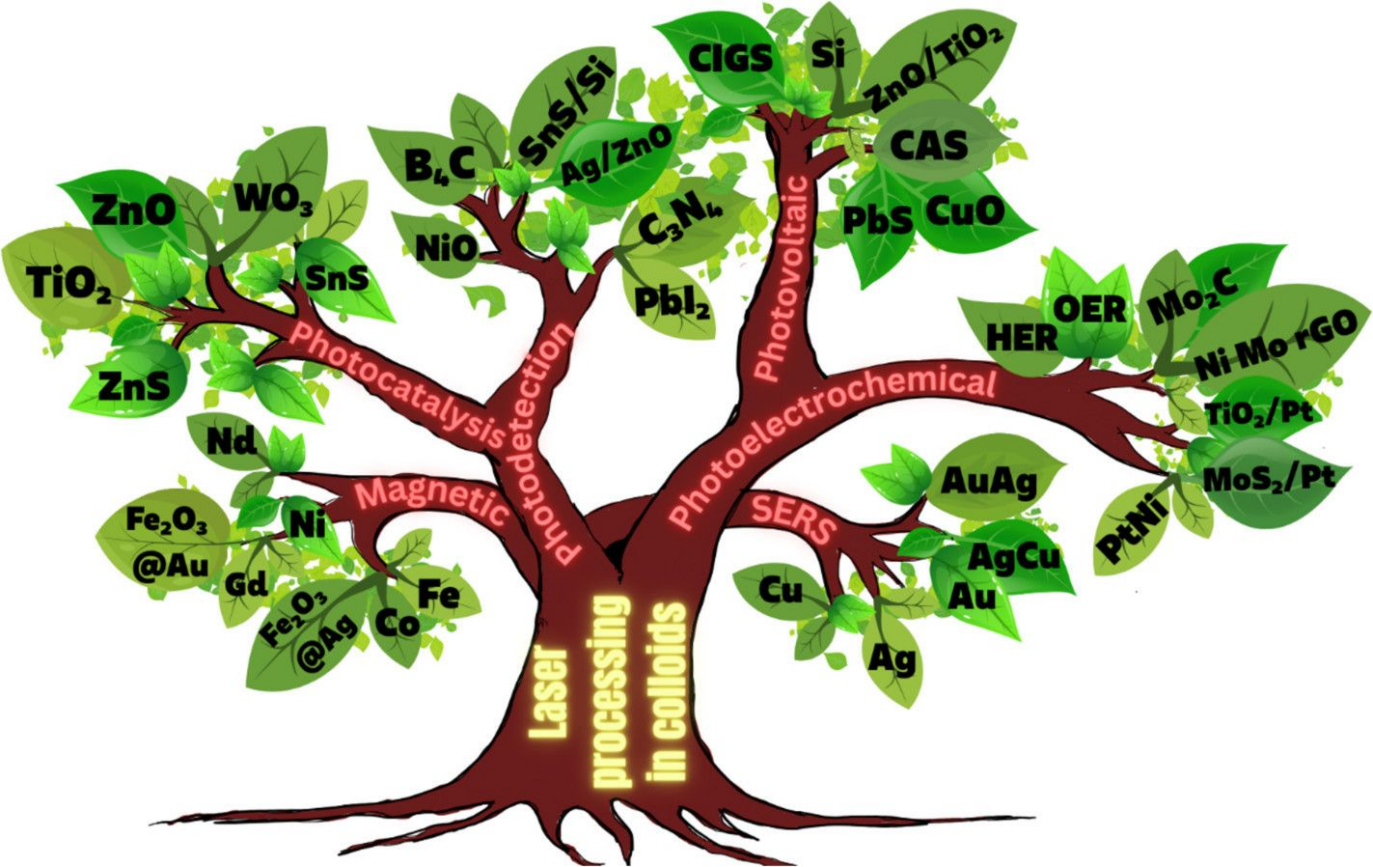
Illustration showing nano materials used for the fabrication of different devices using LPL. All graphical elements including tree and the leaves: ©Jithin Kundalam Kadavath via Canva.com.

A detailed illustration depicting different nanomaterials processed by laser synthesis in liquids and various devices fabricated using them is given in Figure 15.

#### 3.1 Photovoltaics

**Figure 16:**
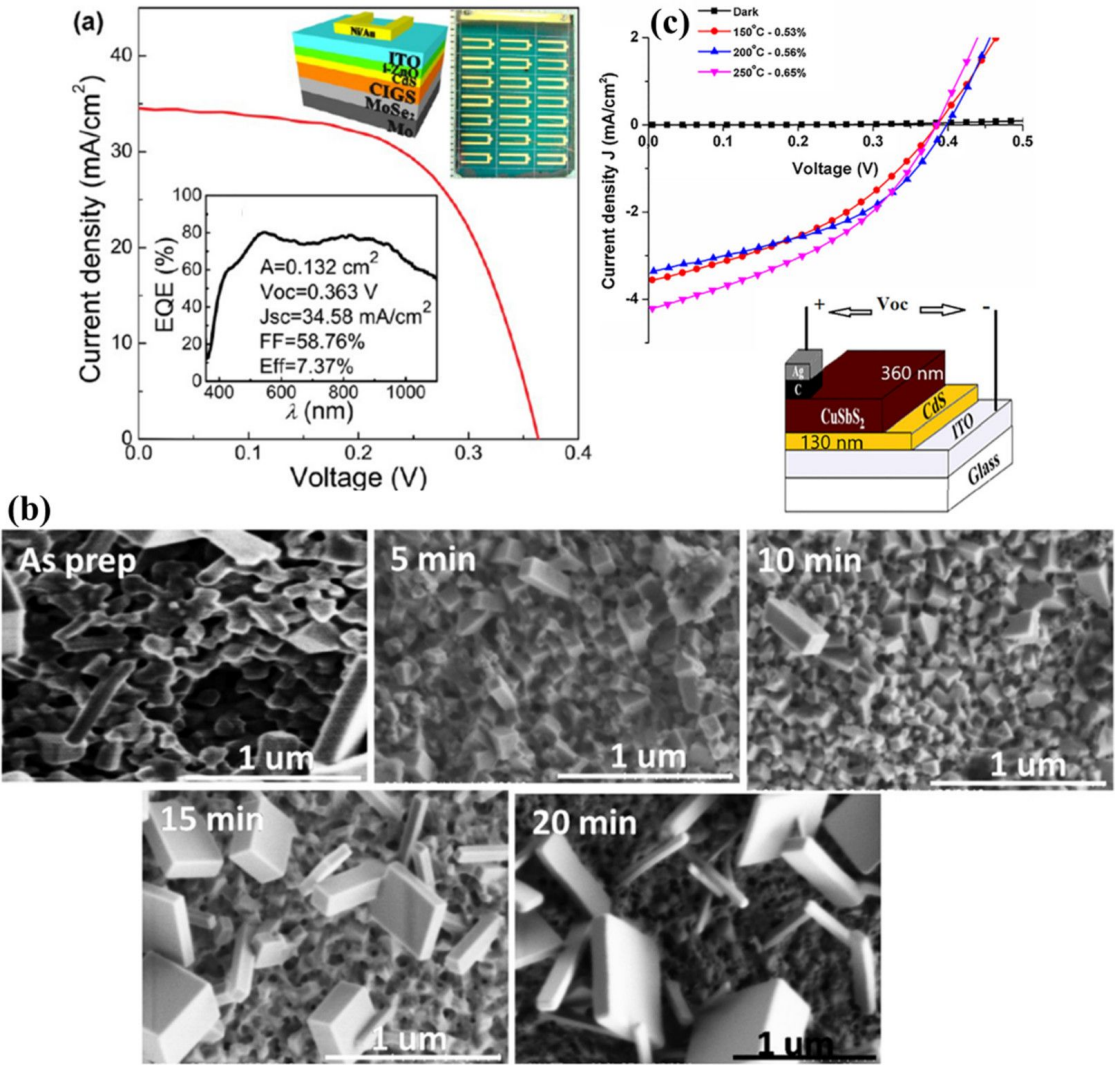
(a) *I*−*V* EQE characteristics measured for a CIGS solar cell on a Mo metal sheet. The inset photo and illustration show the solar cell and its device structure, respectively. (b) SEM images of the CH_3_NH_3_PbI_3_-PbS films prepared using the PbS colloids synthesized for different ablation times as marked. Also, pristine sample CH_3_NH_3_PbI_3_ (as prepared) is given. (c) Solar cell *J*–*V* curves under dark and light of the structure glass/FTO/CdS/Sb_2_S_3_/CAS where the CAS layer is deposited by spraying laser ablated CAS nanocolloids. The schematic of the film stack structure with a circuit diagram is also included. Figure 16a was adapted with permission from [134], Copyright (2012) American Chemical Society. This content is not subject to CC BY 4.0. Figure 16b was reprinted from [130], *Applied Surface Science*, vol. 476, by S. Shaji; V. Vinayakumar; B. Krishnan; J. Johny; S. S. Kanakkillam; J. M. F. Herrera; S. S. Guzman; D. A. Avellaneda; G. A. C. Rodriguez; J. A. A. Martinez, “Copper antimony sulfide nanoparticles by pulsed laser ablation in liquid and their thin film for photovoltaic application”, pages 94–106, Copyright (2019) with permission from Elsevier. This content is not subject to CC BY 4.0. Figure 16c was reprinted from [139], *Applied Surface Science*, vol. 508, by D. A. A. Leal; S. Shaji; D. A. Avellaneda; J. A. A. Martínez; B. Krishnan, “In situ incorporation of laser ablated PbS nanoparticles in CH_3_NH_3_PbI_3_ films by spin-dip coating and the subsequent effects on the planar junction CdS/CH_3_NH_3_PbI_3_ solar cells”, article no. 144899, Copyright (2020) with permission from Elsevier. This content is not subject to CC BY 4.0.

Harnessing solar energy through PV conversion stands out as a leading technology for sustainable and long-term renewable energy generation. Laser-based processes offer unmatched precision and flexibility, enabling innovative, efficient, and scalable material synthesis with minimal environmental impact. Sygletou et al. have reviewed the advanced light-driven methods for fabricating materials for PV and energy storage systems, recent advancements, existing challenges, and future prospects [156]. They indicate the importance of laser-assisted synthesis methods over conventional methods due to their simplicity, rapid execution (often single-step), site-specific applicability, non-contact nature, scalability, minimal environmental footprint, and reduced substrate damage. Additionally, they enable the production of high-purity materials. Numerous reviews explore the use of laser-generated nanocolloids in photovoltaic applications. The key factors influencing the synthesis of colloidal NPs via PLAL were discussed [157]. It also described methods to control the parameters for producing metal and metal oxide NPs suitable for applications like solar cells. Laser ablation and irradiation in acetone and water were used to synthesize of copper oxide and zinc oxide NPs for photovoltaic applications [158]. While the fundamental wavelength of a Nd:YAG laser was used for LAL, a 532 nm wavelength was used for laser-assisted modification of as-prepared NPs in the second stage. The work demonstrates that additional laser irradiation allows for controlled modification of properties of the NPs, enabling tailored material characteristics. A n-ZnO/p-CuO heterojunction was created by spin-coating p-CuO and n-ZnO thin films onto an ITO substrate, followed by an evaluation of the electrical properties of the resulting device. NPs were synthesized using both metallic plates and compressed oxide powder targets with a nanosecond pulsed laser. Experimental conditions, such as laser wavelength and liquid medium, were varied to produce nanomaterials with distinct compositions, structures, and optical properties. The photovoltaic device fabricated, exhibited diode-like behavior and good photoresponsivity, underscoring the potential of laser-prepared metal oxide nanomaterials for low-cost photovoltaic device development. CuO:Cu_2_O NPs were synthesized through laser ablation of copper powder in distilled water [159]. The positively charged particles were selectively deposited onto negatively charged silicon substrates, enabling the development of photodetectors and solar cells. The hybrid junction demonstrates a strong optical response, particularly in the near-infrared range, with satisfactory optical current and conversion efficiency. These results highlight the material’s potential for use in silicon-based solar cells. Zhang et al. published a review focusing on the synthesis of colloidal metal NPs via laser ablation, highlighting their applications in solar cell fabrication [160]. Colloidal metallic NPs of Cu−In and Cu−Ga alloys were produced using a high-energy femtosecond pulsed 1040 nm laser with pulse duration of 600 fs, pulse energy of 10 μJ, and a pulse repetition rate of 500 kHz. High-quality copper indium gallium selenide (CIGS) films were obtained by EPD and 7.37% efficient (active area) CIGS solar cells were fabricated directly on Mo metal sheets (Figure 16a). This approach for fabricating thin film solar cells helps in minimizing chemical impurities. It offers fast deposition rate and high raw material utilization and can also be applied to other materials such as copper zinc tin sulfide (CZTS) [134]. Metallic copper NPs synthesized via laser ablation in acetone and methanol using a 1064 nm nanosecond pulsed laser were integrated between transparent electrodes, with aluminium-doped zinc oxide (AZO) and zirconium-doped indium oxide (IZrO) serving as top and bottom layers, respectively, forming glass/IZrO/Cu NPs/AZO structures optimized for photovoltaic applications. Optical properties are influenced by thermal annealing at 200 °C and the type of embedded NPs. The optimal structure achieved a transmittance of ≈79% in the vis–NIR range, an bandgap of 3.43 eV, diffused transmittance of 2.5–6%, and a sheet resistance of 79 Ω/sq. It demonstrated high performance with an internal quantum efficiency of up to 89% in tandem solar cells [161]. Petridis et al. introduced a plasmonic photon management strategy that incorporates NPs synthesized via LAL into various photovoltaic devices, including inorganic, organic, dye-sensitized, and hybrid solar cells [162]. However, for plasmonic applications, ligand coatings on NPs can cause exciton quenching through non-radiative energy transfer with the active layer. The integration of NPs into different layers, such as active layer and hole transport layer, is discussed in detail. This light-trapping method has demonstrated improvements in power conversion efficiencies and short-circuit currents by up to 22% and 20%, respectively, primarily due to near-field enhancements and scattering effects influenced by NP size. Additionally, the incorporation of NPs enhances device stability under operational conditions by reducing polymer photodegradation in organic photovoltaic systems. A hole-transport-material-free p–n junction perovskite solar cell with the configuration glass/FTO/CdS/CH_3_NH_3_PbI_3_:PbS/C/Ag was fabricated [139]. From the *J*–*V* characteristics of the device, the photovoltaic parameters were derived. The PbS NPs prepared by PLAL added into the solar cells helped in improving the photovoltaic performance. The SEM images of CH_3_NH_3_PbI_3_–PbS films fabricated using PbS colloids are shown in Figure 16b. An increase in current density was observed for the PbS-incorporating devices and was attributed to the higher thickness of the absorber layer. In summary, LAL-synthesized metallic NPs show significant promise for improving light trapping and stability in photovoltaic applications [163–164].

Metallic NPs are not the only materials used to enhance solar cell efficiencies; inorganic or hybrid organic–inorganic materials like perovskites synthesized via LAL have also been extensively studied [165–166]. Studies highlight significant research and engineering advancements in the laser processing of MXenes, metal-organic frameworks (MOFs), and perovskites. Among these, perovskites stand out as prime candidates for optoelectronic applications, especially in photovoltaics due to their high absorption coefficients, long carrier diffusion lengths, and tunable bandgap. While MXenes and MOFs are primarily explored for supercapacitor applications, laser processing of perovskites has been tailored for technologies like LEDs, photodetectors, and solar cells. Mie-resonant NPs are effective tools for nanoscale light control, with applications in coloration, metaoptics, and enhancing perovskite solar cells. However, the influence of nanoparticle size on photovoltaic performance remains underexplored. Furasova et al. [167] used monodisperse Si NPs to examine their optical effects on perovskite solar cells with a bandgap of 1.53 eV. Experimental results revealed that NPs sized 140–160 nm optimize absorption in the 500–800 nm range, where Mie resonances enhance light trapping and scattering. This size-specific optimization improved the perovskite solar cell performance, boosting the short-circuit current density, *J*_sc_, by ≈1.2 mA/cm^2^, and the open-circuit voltage, *V*_oc_, by ≈0.02 V. The findings underline the importance of precise nanoparticle size selection for integrating optical resonances into perovskite devices.

Titania (TiO_2_) NPs have numerous applications in photocatalysis (discussed later in Section 3.3). However, their production through LAL has also been explored for functionalizing titania with polymers in the active layer of hybrid solar cells. By combining the titania NPs with water-soluble poly[3-(potassium-6-hexanoate)thiophene-2,5-diyl], hybrid solar cells have been successfully developed [168]. Two methods for producing titania NPs via laser ablation have been tested, that is, using TiO_2_ powder as the target and employing a solid titanium target. Laser ablation of rutile titania powder yielded NPs with rutile, anatase, and brookite structures, while ablation of a solid titanium target primarily produced rutile. The resulting material demonstrated high fill factors and open-circuit voltages, highlighting its potential for innovative photovoltaic applications. A successful synthesis of a ZnO/TiO_2_ nanocomposite catalyst with improved photovoltaic and photocatalytic performance using PLAL was reported [169]. A nanosecond 532 nm Nd:YAG laser at 350 mJ/pulse and a frequency of 10 Hz was used for the synthesis process in distilled water. The PLAL method creates a stable bond between TiO_2_ and ZnO NPs. The ZnO/TiO_2_ nanocomposite achieved a power conversion efficiency of 6.7%, with *J*_sc_ = 2.02 mA, *V*_oc_ = 0.5 V, and a fill-factor of 0.53. The performance improvement is attributed to oxygen vacancy defects that trap charge carriers and reduce electron–hole recombination.

The efficiency of photovoltaic cells can be enhanced using Si NPs [170]. The ultrasmall Si NPs exhibited PL when excited by UV light, enable them to act as secondary light sources that convert high-energy photons into visible-range photons. This downshift offers a promising strategy to boost PV performance across different solar cell types [171]. Polycrystalline solar cells with an initial efficiency of ≈10% were coated with luminescent Si NPs. The coated cells demonstrated a 1.64% improvement in external quantum efficiency compared to uncoated reference cells, attributed to the downshift effect of Si NPs. A QD-sensitized solar cell was developed using Si NPs (6 nm) synthesized via LAL [172]. The NPs served as the photosensitizer in a solar cell with a polysulfide electrolyte. The solar cell with Si NPs exhibited a 5.3-fold higher conversion efficiency compared to one with only TiO_2_ particles. Electrical impedance spectroscopy showed that the reduced resistance in the Si NP-based cell led to a higher current density. A review by Makhdoom et al. highlights advancements in the synthesis, modification, and processing of free-standing Si NPs (≤50 nm) for photovoltaic applications, including progress in solution-processed Si NP solar cells for next-generation photovoltaics [173].

The individual contributions of metallic NPs and Si NPs to improve the photovoltaic performance have been discussed separately. In one study, both properties are combined and utilized in Ag@SiO_2_-based PV devices [174]. Ag@SiO_2_ core–shell NPs, synthesized via laser ablation, were integrated into dye-sensitized solar cells (DSSCs) to enhance their performance through plasmonic effects. Current–voltage characteristics were measured under standard conditions and compared to bare DSSCs without NPs. The incorporation of Ag@SiO_2_ NPs significantly improved solar cell efficiency, showing a 27% increase compared to conventional DSSCs. These NPs act as selective scattering agents, making them a promising component for efficient plasmonic-sensitized solar cells.

Solar cells were fabricated to demonstrate the photovoltaic application of the spray deposited copper antimony sulfide (CAS) thin films using laser-ablated nanocolloids of CAS. A superstrate configuration of glass/FTO/CdS/CAS was used. The fabricated solar cells were heated at different temperatures in vacuum to improve the top electrode contact and performance. Although the obtained efficiencies were low, the *J*–*V* curves (Figure 16c) showed good p–n junction characteristics between the n-type CdS and p-type CAS layers, indicating that depositing CAS thin films from laser-ablated nanocolloids is an effective method for solar cell fabrication. To further improve efficiency, an intrinsic Sb_2_S_3_ layer was introduced between CdS and CAS [130]. Selenium is a critical element for photovoltaics and energy storage, classified as “energy-critical” by the American Physical Society and Materials Research Society. Various methods, such as wet chemistry, vapor-phase growth, and pulsed laser ablation, are used to synthesize selenium nanostructures. For the first time, nanoneedles were synthesized via pulsed laser ablation of a bulk selenium target in organic solvents, using a nanosecond Nd:YAG laser [175]. The laser’s repetition rate allows for tuning aspect ratio, sharpness, and diameter of the nanoneedles.

Nanoparticles offer a promising bottom-up approach for improving thin film photovoltaics [176]. They can be used as components like CIGS, perovskite, or QDs, with traditional materials like Si and GaAs awaiting new techniques to be used as QDs in solar cells. Nanoparticles also play a role in light management, with plasmonic particles enhancing light absorption and dielectric scatterers functioning as antennas to direct light into the active layer. Additionally, photon up- and down-conversion materials can be made from NPs. While wet chemical methods can produce NPs in large quantities, challenges include unwanted chemicals and low crystallinity. Some materials, like Si and Ge, are difficult to produce as NPs. LPL thus helps in advancements in nanoparticle-based thin film solar cells. Combining these research areas opens up exciting possibilities for the future of solar technology [176]. The laser-synthesized nanomaterials utilized so far for solar devices are CuO, ZnO, Cu-In, Cu-Ga, CIGS, CZTS, TiO_2_, Si NPs, Ag@SiO_2_, CAS, and selenium nanoneedles. Out of these, metal oxide NPs are widely used for UV absorption, and metal sulfides are ideal for the visible and NIR ranges. Laser-generated NPs in heterojunctions, light trapping, plasmonic enhancements, and hybrid organic–inorganic devices can be applied for further improvement in efficiency. Integration techniques like spin coating, EPD, and spray deposition are commonly used for fabrication of devices. Performance improvements through enhanced light absorption, optimized particle sizes, and improved charge carrier dynamics can be achieved.

#### 3.2 Photodetectors

In the ever-evolving world of optoelectronic devices, photodetectors play a vital role in a wide range of applications, from optical communication systems and imaging technologies to environmental sensing and biomedical diagnostics [177–178]. These devices, which convert light into an electrical signal, are central to many modern technologies, and as such, their performance and efficiency are constantly being improved. The efficiency of a photodetector depends on various factors, including the material’s light absorption capability, carrier mobility, and the mechanism of charge separation [177]. Conventional photodetectors rely on bulk materials such as semiconductors, metals, as well as organic compounds and the performance of these materials can be limited by issues such as slow response times, poor sensitivity, and low charge carrier mobility. Modifications with NPs and nanostructures are particularly advantageous for photodetector applications because of their unique optical properties, such as strong light absorption, high surface-to-volume ratio, and the ability to undergo surface plasmon resonance (SPR) [179–180]. Recently, there has been significant interest in employing laser-synthesized nanocolloids to enhance the capabilities of photodetectors. The liquid nature of nanocolloids allows for flexible integration into various device architectures, providing opportunities for novel form factors. The integration of laser-synthesized nanocolloids into photodetectors can take many forms, ranging from the simple deposition of nanocolloid films to more sophisticated hybrid devices which are explained in Section 2 of this review.

Photodetectors with various device structures have been developed using laser-processed nanocolloids, offering a wide range of optoelectronic applications. The operating modes include photoconductive detectors, which rely on the change in conductivity under light exposure; photodiodes, which convert light into electrical current in a junction; photovoltaic sensors, which directly convert light into electrical power; and heterojunction photosensors, which use the interface between different materials to enhance performance [181]. By combining EPD with NP preparation via laser ablation, Jeon et al. have successfully assembled a size-controlled QA nanoparticle colloid into a photoconductive thin film on an ITO electrode [137]. 355 nm laser pulses of 7 ns and 10 Hz repetition rate were used for ablation of QA microcrystals in water. The QA films exhibited a photoconductive response comparable to that of conventional organic films produced by vacuum deposition [137]. The preparation of CuO NPs through LAL for photodiodes has been reported [114]. Additionally, the results of C_3_N_4_ NPs, synthesized by PLAL of graphite in an ammonium solution and deposited on silicon substrates by spray, suggest that these materials are promising candidates for high-efficiency photodiodes [182–183]. Further discussion on photodiode-based detectors and other heterojunction photodetectors is also provided later in the article. The integration of laser-processed nanocolloids enhances the efficiency and sensitivity of these photodetectors, making them suitable for diverse applications in imaging, sensing, and energy harvesting.

Heterojunction photodetectors prepared on Si using NPs prepared by LAL are an innovative approach for enhancing the performance of photodetectors. The bandgap of CuO NPs on n-Si was modified by decorating it with Au NPs. The UV-based detector with AuNPs had a higher lifetime of photogenerated carriers than the CuO/Si photodetector, and the maximum values of quantum efficiency were about 41% and 78% at 700 nm for CuO/Si and AuNPs–CuO/Si photodetectors, respectively [183]. In some cases, nanocolloids were incorporated into composite materials, where their unique properties synergize with other components, such as semiconductors, to enhance the device’s overall performance. For instance, three configurations of photodetectors were reported, Ag NPs/Si, Ag NPs/ZnO/Si, and ZnO/Ag NPs/Si, and the *I*–*V* characteristics of the ZnO/Ag NPs/Si photodetector revealed significantly enhanced conductivity in comparison to the other two counterparts. Remarkably, the ZnO/AgNPs/Si photodetector exhibited the highest responsivity value of 132 A·W^−1^, accompanied by quantum efficiency of 429.88, sensitivity of 31,400%, gain of 315, detectivity of 10^10^ Jones, and a noise equivalent power (NEP) of 0.556 × 10^−13^ W, attributed to the localized surface plasmon resonance (LSPR) phenomenon exhibited by the Ag NPs [184]. The use of Ag or Au NPs in photodetectors can enhance the light–matter interaction through LSPR, improving the absorption of specific wavelengths of light [185].

**Figure 17:**
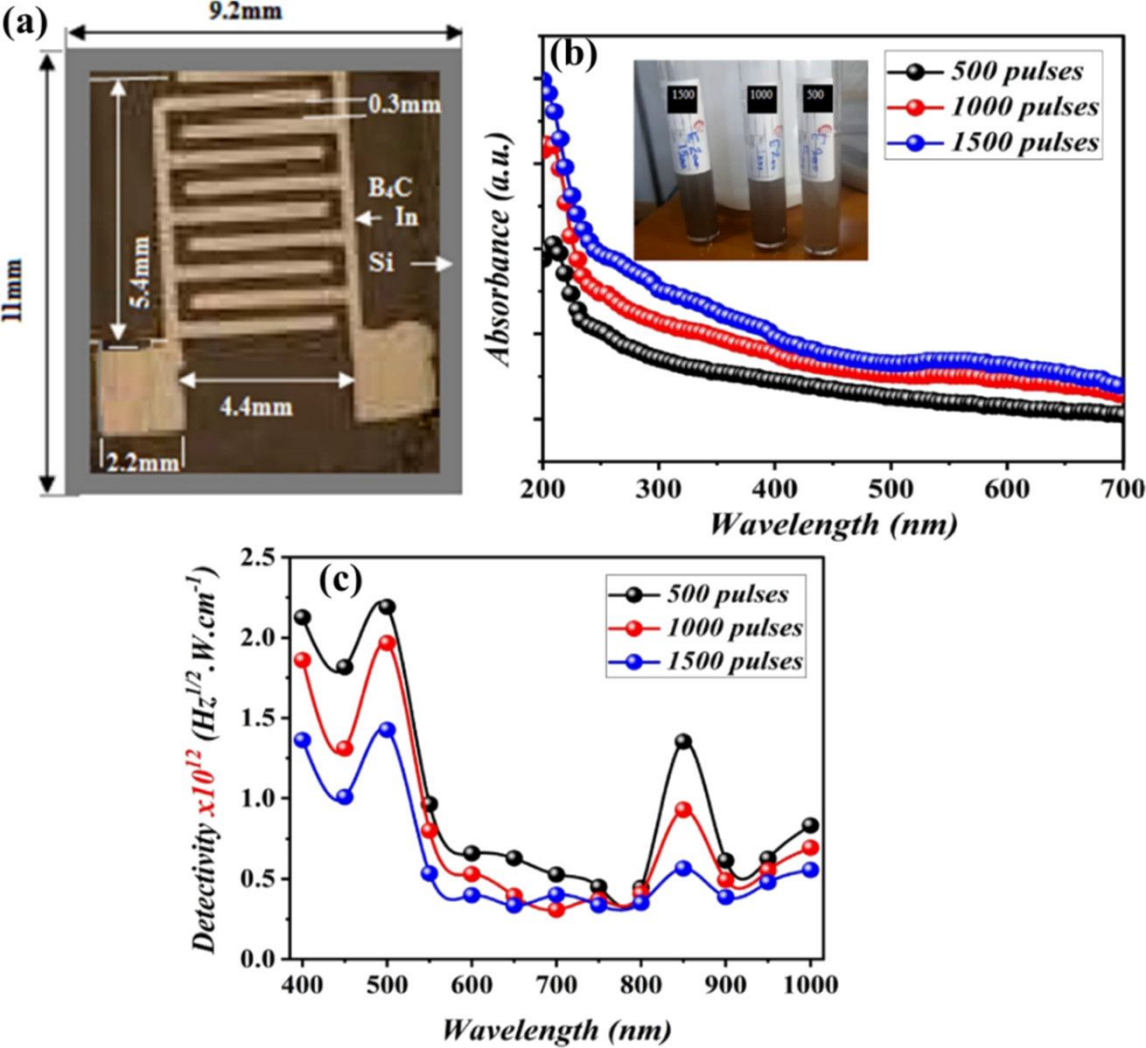
(a) Optical top-view image of the B_4_C/p-Si heterojunction photodetector. (b) Optical absorption of B_4_C colloid prepared at different number of laser pulses. (c) Effect of laser pulses on specific detectivity for B_4_C/p-Si photodetectors fabricated at different number of laser pulses. Figure 17a–c was reproduced with permission from [186] (© 2022 S. S. Hamd et al., published by Springer Nature, distributed under the terms of the Creative Commons Attribution 4.0 International License, https://creativecommons.org/licenses/by/4.0).

The laser ablation of boron carbide (B_4_C) NPs was reported for the first time by pulsed laser ablation of boron in ethanol using a Nd:YAG laser with a wavelength of 532 nm, a pulse duration of 7 ns, and repetition frequency of 6 Hz. As the number of laser pulses varied from 500 to 1500 pulses, it had significant effect on the morphological, optical, and electrical properties. The spectral responsivity of the p-B_4_C/p-Si photodetector showed a maximum responsivity of 0.66 A·W^−1^ at 500 nm for a photodetector prepared at 500 pulses. Figure 15a displays an optical top-view image of a representative B_4_C/p-Si device, and Figure 17b shows the optical absorption spectra of B_4_C colloids synthesized using varying numbers of laser pulses, highlighting the tunability of absorption characteristics. The highest specific detectivity (Figure 17c) and quantum efficiency were 10^12^ Jones and 164% at 550 nm, respectively. This was explained as a result of an increase in noise current with the number of pulses [186]. NiO NPs were synthesized by varying laser energies between 400 and 800 mJ and were coated onto porous silicon (PS) via light-assisted electrochemical etching. A Nd:YAG laser of wavelength 1064 nm and repetition rate of 10 Hz with pulse duration of 6 ns was used for ablation in water. The photodetector measurements indicate that the NiO NPs/PS structures, formed at 700 mJ, exhibited maximum responsivity. The signal-to-noise ratio increased with an increase in laser pulse power, and the detection ability improved. An increase in laser energy above 700 mJ resulted in a minor shift of the peak responsivity in the spectra towards greater wavelengths, suggesting modifications of the bandgap of the NiO NPs [187]. ZnO NPs generated by laser ablation or pulsed laser irradiation in liquid were widely studied for the use in UV photodetectors due to their excellent properties [188]. As an n-type semiconductor with an optical bandgap of around 3.4 eV, ZnO belongs to the category of transparent conductive oxides, offering high electrical conductivity and optical transmission. ZnO nanostructures find diverse applications in the field of photocatalysis, biomedicine, optoelectronics, and gas sensors as they exhibit unique properties like large surface area, tunable bandgap, high surface reactivity, and strong photoluminescence. ZnO can form various morphologies, including NPs, nanorods, and nanowires, depending on the preparation method [189–190]. ZnO NPs generated using a 1064 nm, Q-switched, nanosecond Nd:YAG laser of different pulse energies (500, 700, and 900 mJ) in laser ablation exhibited a shift in absorption wavelengths with an increase in laser pulse energy. A photodetector of configuration Ag/ZnO NPs/PS/n-Si/Ag showed greater current flow than a Ag/PS/n-Si/Ag structures when a forward bias was applied without light. Using PS as a base material for forming thin films of ZnO NPs improved the absorption and light-trapping properties. Using 700 mJ was the best condition [191]. Though both NiO and ZnO have high resistance and surface imperfections that cause electron–hole recombination to occur quickly, ZnO is reported to be better than NiO as a photodetector. The performance of both materials can be improved by doping, annealing treatment to reduce losses [118]. CuO NPs on PS are also another widely explored material. CuO NPs were generated using PLAL in ethanol and water using a laser of wavelength 1064 nm, pulse width of 10 ns, and repetition frequency of 1 Hz and deposited on the PS substrate via photo-assisted electrochemical etching. The CuO nanocolloids exhibited a linear relationship between laser power and absorption, and their SPR peaks were reported at 570–590 nm. For CuO NPs in water and ethanol, agglomerated spherical distributed clusters were reported. Bandgaps between 1.61 and 1.90 eV were found for CuO NPs produced at laser energies between 500 and 800 mJ, respectively. The greatest responsivity was at an energy of 700 mJ and was 0.135345 A·W^−1^ at 450 nm, as determined by the optoelectronic characteristics [114,192–193]. Nanostructured core–shell structures of CuO@ZnO NPs synthesized using different pulsed laser ablation energies (500–900 mJ) for 1064 nm wavelength in distilled water and its detection properties were also reported [194]. These results highlight the critical role of selecting the appropriate laser pulse energy to effectively tune the properties of materials for detector applications.

**Figure 18:**
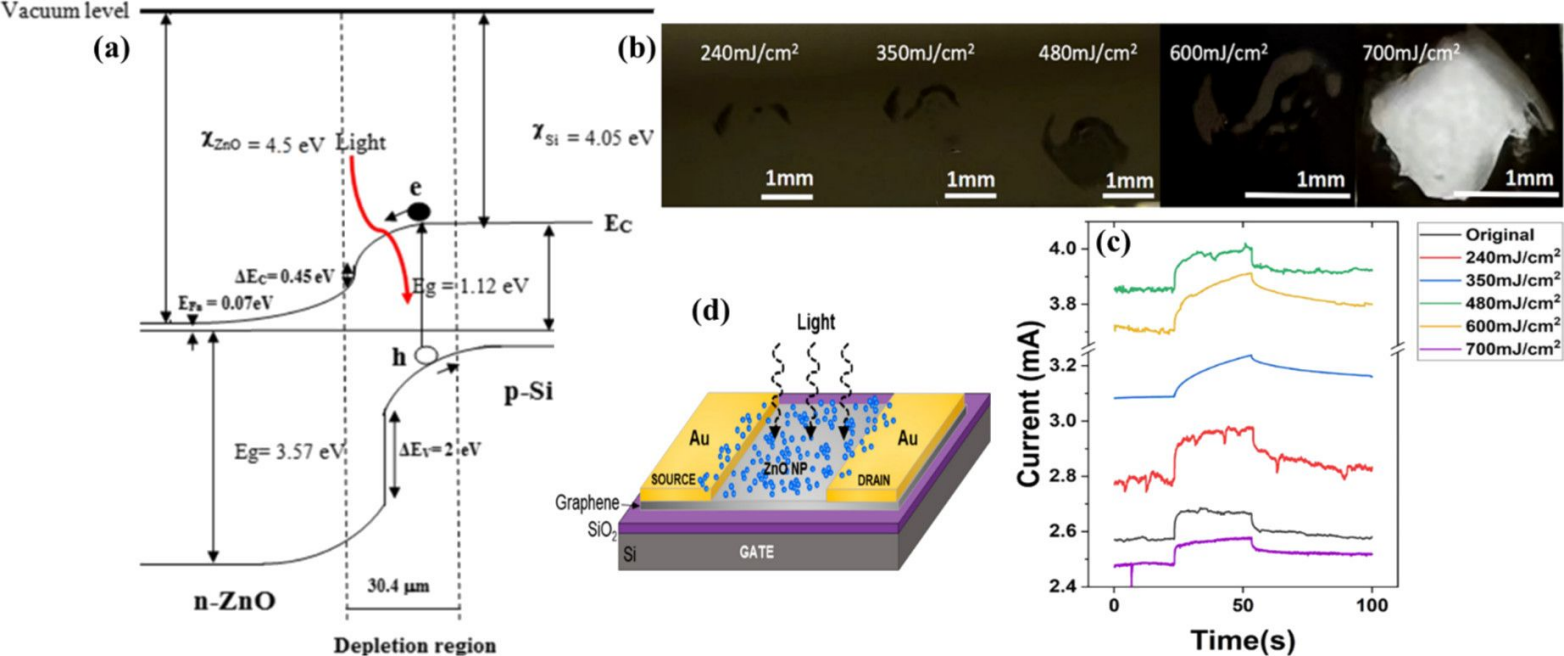
(a) Band lineup of n-ZnO/p-Si heterojunction prepared at 4.2 J/cm^2^ under illumination. (b) Images of the ITO thin film surfaces after laser irradiation at different laser fluences. (c) The ON/OFF response of the photocurrent under illumination with a blue light source (460 nm, 2.8 × 10^−3^ W/cm^2^) for the ITO/ZnO/ITO devices before and after ns laser irradiation at different fluences. (d) Schematic of the ZnO nanoparticle/graphene phototransistor architecture (not to scale). Figure 18a is from [190] (H. F. Abbas et al., “Fabrication of High-Performance ZnO Nanostructure/Si Photodetector by Laser Ablation”, *Silicon*, vol. 16, pages 1543–1557, published by Springer Nature, 2023, reproduced with permission from SNCSC). This content is not subject to CC BY 4.0. Figure 18b,c was reprinted from [196], with the permission of AIP Publishing. This content is not subject to CC BY 4.0. Figure 18d was reproduced from [33], (© 2020 K. Charipar et al., published by MDPI, distributed under the terms of the Creative Commons Attribution 4.0 International License, https://creativecommons.org/licenses/by/4.0).

In another study, the effect of laser fluence was explored for ZnO NPs in distilled water. The ablation was carried out for 30 min using a pulse duration of 7 ns and a pulse repetition rate of 2 Hz. The bandgap of ZnO NPs increased from 3.47 to 3.64 eV as the laser fluence increased from 3.6 to 4.7 J/cm^2^. Photodetector properties of spherical ZnO NPs with sizes ranging from 45 to 85 nm were reported. Agglomeration of NPs was observed at high laser fluence. The ZnO/Si photodetectors with ohmic contacts, formed by depositing a thick Al film on the silicon substrate and an In film on ZnO through an interdigitated mask fabricated at fluence of 4.2 J/cm^2^, demonstrated a responsivity of 9.9 A·W^−1^, quantum efficiency of 295.1%, and detectivity of 4.3 × 10^12^ Jones at 450 nm under a bias voltage of −5 V [190]. Figure 18a shows the band alignment of the fabricated n-ZnO/p-Si heterojunction. The maximum detectivity and external quantum efficiency were obtained for the photodetector fabricated with 4.2 J/cm^2^. Similar studies on the effect of laser fluences and laser energies have been reported on the detection properties of ZnO NPs synthesized in alcohols on n-Si. Unlike in water, a mixture of spherical and hexagonal particles was reported in ethanol with the average particle size varying from 65 to 75 nm when the laser fluence was increased due to increasing the amount of the ablated material [189,195]. One of the major drawbacks of ZnO NP-based detectors is their operating wavelength in the UV–vis region due to their bandgap values. Zheng et al. have employed laser irradiation using 4 ns pulses from a 1064 nm Nd:YAG laser operating at 30 Hz. The irradiation with different fluences onto sputtered ZnO/ITO films was used to engineer the concentration of laser-induced defects, such as oxygen vacancies, to modify the electrical and optoelectrical properties. Figure 18b presents optical images, and Figure 18c compares the ON/OFF photocurrent response of ITO/ZnO/ITO devices under blue light. Defects produced by laser irradiation introduce new energy levels within the bandgap of ZnO, which narrows the effective bandgap resulting in a detection in longer wavelengths [196]. There is a limited body of research on photodetectors using nanocolloids synthesized through laser fragmentation in liquids. ZnO nanoparticle/graphene phototransistor (Figure 18d) that has a responsivity up to 4 × 10^4^ A·W^−1^ and gain of up to 1.3 × 10^5^ with UV wavelength selectivity were synthesized by pulsed laser fragmentation of ZnO powder in water [33]. NPs of Al_2_O_3_ were created using laser ablation of an Al target in deionized water using three different laser fluences of a 1064 nm Nd:YAG laser. The UV–vis photodetector showed strong responsiveness with values of about 1.2 A·W^−1^ [197]. All these studies underscore the pivotal role of selecting laser fluence during synthesis for optimizing photodetector device performance.

Our group has reported semiconductor chalcogenide-based photodetectors of SnS and its hybrids SnS–Si and SnS–graphene for UV–vis–NIR photodetection. The SnS photodetectors were fabricated using nanocolloids prepared by PLAL and PLFL techniques [104,108]. The impact of laser wavelength on nanoparticle generation and its subsequent influence on detector device fabrication and performance is thoroughly discussed [104]. Nanocolloids produced using a 1064 nm wavelength demonstrated superior performance compared to those generated with a 532 nm wavelength. Additionally, the SnS–Si hybrid was a pioneering development, offering detection capabilities up to 1064 nm [107]. The SnS–graphene hybrid was also introduced as a wavelength-independent detector, with a specific detectivity of 10^7^ Jones and a responsivity of 10^−5^ A·W^−1^ [108]. SnO_2_ NPs generated by irradiation of tin target in methanol and aqueous NaCl solution were reported to have a bandgap of 3.8 eV and 3.95 eV, respectively. The n-SnO_2_NPs/p-Si heterojunction photodetectors had a responsivity of 0.53 A·W^−1^ in NaCl solution, while the responsivity was 0.43 A·W^−1^ in methanol at 410 nm [198]. Carbon nitride (CN) heterojunction photodetectors were fabricated by deposition of CN NPs on p-Si substrate with photoresponsivity of 2.33 A·W^−1^ at 600 nm [182]. Another heterojunction photodetector of hybrid In/p-MWCNTs on n-Si showed two peaks of response at 650 and 850 nm with a responsivity of 0.53 A·W^−1^ at 532 nm, and the detectivity was of the order of 10^12^ Jones. The preparation of multiwalled carbon nanotubes (MWCNTs) and carbon nanoparticles were using pulsed laser ablation of a graphite target in water [199]. Core–shell NPs of Au@LiNbO_3_ were synthesized by means of laser ablation in ethanol without using a catalyst. The optoelectronic properties of the Au@LiNbO_3_/Si show that the maximum responsivity was 0.43 A·W^−1^ at 400 nm for the photodetector fabricated at 2 J/cm^2^. The authors mention the importance of selecting solvents for laser ablation for different applications. They confirmed that the characteristics of core–shell particles synthesized in ethanol were better than those obtained in distilled water due to the lesser particle agglomeration [200].

**Figure 19:**
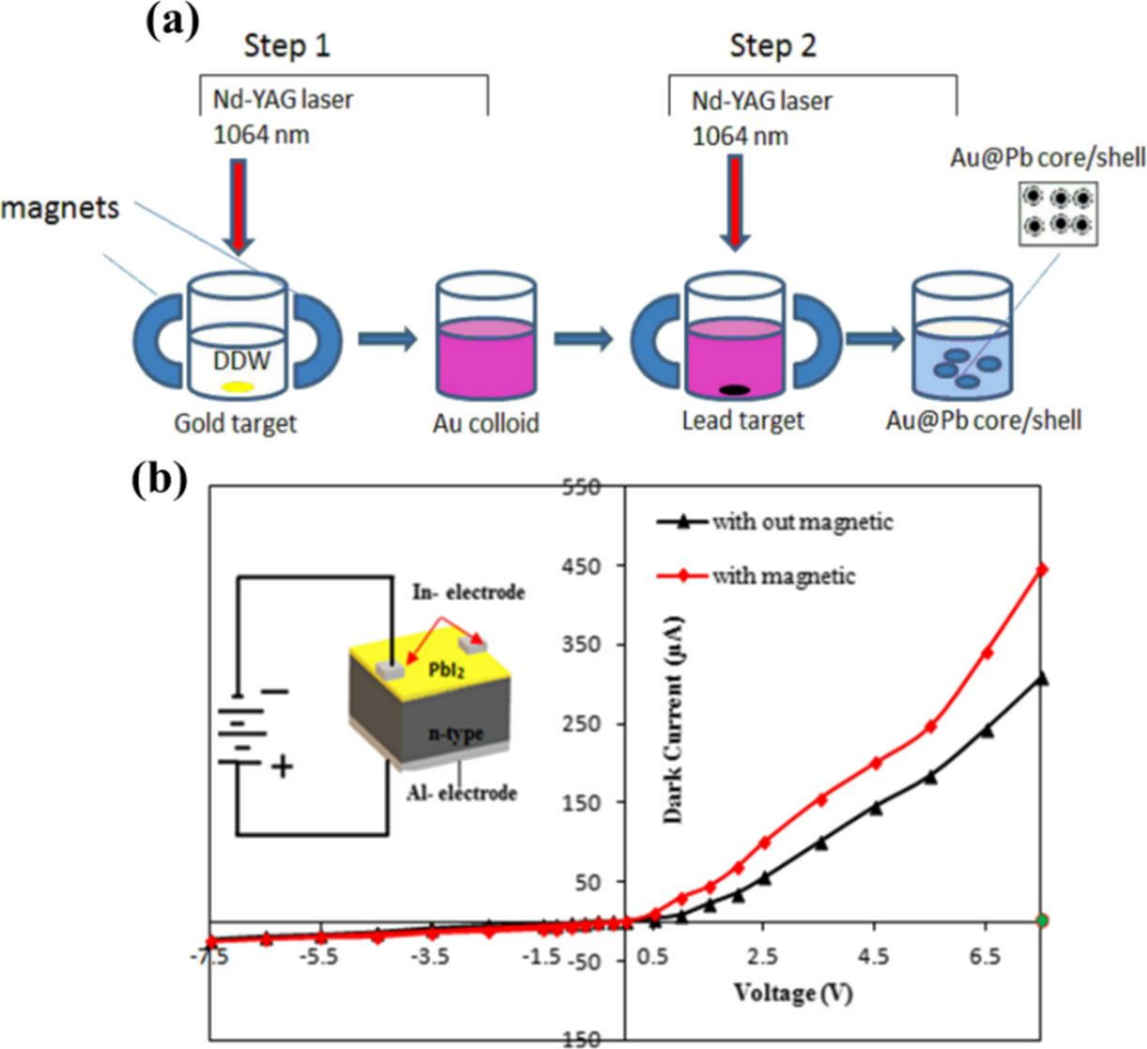
(a) Experimental frame-work for the PLAL assisted magnetic field. (b) Dark *I*–*V* characteristics of the p-PbI_2_/n-Si heterojunction. Figure 19a is from [201] (Z. A. A. Hameed et al., “Two-step Laser Ablation in Liquid-assisted Magnetic Fields for Synthesis Au:Pb Core/Shell NPs in Developing High-Performance Silicon-based Heterojunction Photodetector”, *Plasmonics*, vol. 19, pages 457–469, published by Springer Nature, 2024, reproduced with permission from SNCSC). This content is not subject to CC BY 4.0. Figure 19b is from [202], (R. A. Ismail et al., “Preparation of nanostructured PbI_2_/Si photodetector by magnetic field-assisted laser ablation in liquid”, *Silicon*, vol. 14, pages 10291-10300 by published by Springer Nature, 2022, reproduced with permission from SNCSC). This content is not subject to CC BY 4.0.

The influence of applying a magnetic field during laser ablation and its effect on the figures of merit of detectors has also been reported. Figure 19a shows the experimental setup for Au:Pb NPs/PS composites synthesized by PLAL in an applied magnetic field. The optical bandgap for Au:Pb and lead iodide (PbI_2_) NPs increased in the presence of a magnetic field. The magnetic field method is a reliable technique for enhancing the energy of laser pulses used to ablate bulk material for nanoparticle production. It is recommended as an effective approach to achieving smaller nanoparticle sizes and higher concentrations. The *I*–*V* properties and responsivity of the vis Au:Pb and vis–NIR p-PbI_2_/n-Si heterojunction photodetectors improved significantly when a magnetic field was applied [201–202]. Figure 19b shows the dark *I*–*V* characteristics of the p-PbI₂/n-Si heterojunction device.

Apart from photodetectors, laser-processed colloids have also been used for optoelectronic applications like non-linear optics. Nonlinear optics is a crucial area of research with numerous applications across different fields [203]. Nanoparticles hold significant promise in this domain due to their ability to be precisely engineered and their enhanced nonlinear optical properties, which often outperform those of bulk materials. The response of nanocomposites of a Ag@Au core–shell nanostructured material was better than that of just Ag metallic NPs synthesized by LAL [204]. Cu_2_O NPs of different particle sizes were prepared by varying the repetition rates of pulsed laser ablation of Cu immersed in ultrapure water at room temperature [205]. Chen et al. have explained in detail in their review article on functional nonlinear optical NPs synthesized by laser ablation [203].

NPs synthesized through laser-based methods, particularly PLAL, offer unique optical and electronic properties including strong light absorption, SPR effects, and high surface-to-volume ratios that enhance photodetector sensitivity, speed, and efficiency. The improvements in photodetector parameters such as responsivity, detectivity, and quantum efficiency are achieved with laser-synthesized nanocolloids, such as ZnO NPs for UV detection with tunable bandgaps, and CuO NPs enhanced with Au NPs. Ag NPs/ZnO/Si configurations showed high responsivity and detectivity due to LSPR. Other notable materials for photodetectors include NiO, SnS, SnO_2_, C_3_N_4_, and MWCNTs. All demonstrate performance tuning through laser synthesis conditions. Applying magnetic fields during laser ablation enhances nanoparticle quality for Au@LiNbO_3_/Si core–shell NPs. Additionally, laser-processed colloids are being explored for nonlinear optical applications with nanocomposites like Ag@Au.

#### 3.3 Photocatalysts

Photocatalysis, a rapidly advancing field, has experienced remarkable progress driven by the emergence of laser-synthesized particles as a compelling approach to enhance the efficiency and versatility of photocatalytic systems. These laser-generated particles, possessing unique physicochemical properties, offer unprecedented opportunities for harnessing the power of light to drive chemical transformations, making them a subject of growing interest within the scientific community. At the core of photocatalysis lies the intricate interplay between light, semiconductor materials, and chemical reactions [206]. When a semiconductor photocatalyst, such as TiO_2_ or CdS, is illuminated with light of sufficient energy, it can generate electron–hole pairs, which then initiate a cascade of redox reactions at the material’s surface. These photogenerated charge carriers can participate in the oxidation or reduction of adsorbed species, enabling the degradation of organic pollutants like dyes and phenols and a wide array of other chemical transformations, such as the production of hydrogen fuel and the synthesis of valuable chemicals [207–208]. The efficiency of these photocatalytic processes is largely determined by the semiconductor’s ability to effectively absorb and utilize the incident light, as well as its capacity to separate and transport the generated charge carriers to the surface for the desired reactions [209–210]. Laser-synthesized particles, produced through advanced laser-based techniques, exhibit a range of characteristics that set them apart from traditional semiconductor photocatalysts.

The key advantage of these laser-synthesized materials is the precise control enabled by the laser synthesis process, which allows for fine-tuning size, morphology, and surface properties [87,211–215]. Recent studies have demonstrated that laser-generated TiO_2_ NPs can have their size and morphology precisely engineered, leading to a 20% increase in photocatalytic degradation of organic pollutants compared to conventionally synthesized particles [216]. Table 4 presents a list of nanomaterials generated using pulsed laser ablation, along with the corresponding laser parameters and sample morphologies. Furthermore, experimental data has shown that laser-generated CdS particles exhibit a 30% improvement in charge separation efficiency and a 15% increase in charge carrier mobility, owing to the precise control of the physical and electronic structure of these materials. This remarkable level of control facilitates the tailoring of optical absorption, charge carrier dynamics, and surface reactivity, unlocking the full potential of these laser-generated particles and paving the way for significant advancements in energy and environmental applications [212,214,217]. The direct conversion of solar light into chemical energy via photocatalysis is a focal point for sustainable energy development and environmental remediation [218].

**Table 4:** Various laser parameters, including laser source, fluences, irradiation time, and solvents of PLA-processed metal and alloy NPs. Table 4 was reproduced from [214] (© 2022 Theerthagiri et al., published by Springer Nature, distributed under the terms of the Creative Commons Attribution 4.0 International License, https://creativecommons.org/licenses/by/4.0).

No.	Metal targets	Solvent	Products	Laser source	Particle size	Morphology
	Condition					

1	Ag and Au plates	water	Ag and Au sols	Cu vapor	20–60 nm	disk-shaped
	λ = 510.6 nm; 32 J·cm^−2^ for Au-NPs; 300 J·cm^−2^ for Ag-NPs					

2	Ag metal	aqueous solution of NaCl	Ag-NPs	Nd:YAG	5–50 nm	NPs
	λ = 1064 nm; 5 ns; 10 Hz, focal length 200 mm					

3	Au metal	water	Au NPs	KrF excimer	not specified	not specified
	λ = 248 nm; 25 ns; 10 Hz; 2 J·cm^−2^					

4	Ag and Au-target	water	Ag and Au NPs	Nd:YAG, Q-switched Nd:YAG	16–26 nm, 12–20 nm	spherical
	λ = 355, 532, and 1064 nm; 3–6 ns; 10 Hz; 8.92, 12.74, and 19.90 J·cm^−2^					

5	Pd foil	ethanol–water	Pd NPs	Nd:YAG	3–6 nm	NPs
	λ = 355, 532, and 1064 nm; 3–6 ns; 10 Hz; 8.92, 12.74, and 19.90 J·cm^−2^					

6	Pd plate	aqueous solution of SDS	Pd NPs	Nd:YAG	4.5–12.3 nm	spheroidal NPs
	λ = 1064 nm; 12 ns; 10 Hz, 1.6–2000 J·cm^−2^					

7	Pd metal	water; water–methanol mixture; SDS as surfactant	Pd NPs	Nd:YAG	17 ± 6 nm, 24 ± 7 nm, 27 ± 9 nm (varied by fluence)	spherical NPs
	λ = 1064 nm; 10 ns; 10 Hz, 40.5–8.4 J·cm^−2^					

8	Pt target	ethanol–TSC solution	Pt-NPs	Nd:YAG	7–9 nm	spherical NPs
	λ = 1064 nm; 10 ns; 10 Hz, 40.5–8 J·cm^−2^					

9	Pt metal plate	aqueous solution of SDS	Pt NPs	Q-switched Nd:YAG	10–12 nm	NPs
	λ = 532 and 1064 nm; 10 ns; 10 Hz; 1.6 J·cm^−2^					

10	silicon wafer	ethanol–water	Si NPs	Nd:YAG	1–7 nm	spherical at low fluence; agglomerated NPs at high fluence
	λ = 1064 nm; 10 ns; 10 Hz, 50–200 mJ·pulse^−1^					

11	Zn foil	THF	Zn NPs	picosecond laser system	4.5 nm	NPs
	λ = 515 nm; 10 ns; 10 Hz; 125 μJ; focal area 3.75 m/s					

12	silicon wafer	NA	Si NPs	Nd:Y: Al garnet laser	3–6 nm	NPs
	λ = 532 nm; 10 ns; 10 Hz; 200 mJ; under He flow in ablation chamber ≈900 °C					

13	AuAg solid foils	methyl methacrylate	AuAg alloy	picosecond pulse laser system	5–15 nm	clusters of NPs
	λ = 515 nm; 7 ns; 33.3 Hz; 125 μJ; focal length 56 mm					

14	AuAg targets	acetone solution of HAuCl_4_ and AgNO_3_	Ag0.65Au0.35, Ag0.5Au0.5, Ag0.35Au0.65	femtosecond laser pulse	≥20 nm	spherical NPs
	λ = 532 nm; 40 fs; 10 Hz; 150 μJ; 40 min; focal length 3 mm					

15	Ag foil, Au foil	water	Ag_100−_*_x_*Au_x_ bulk alloy	Nd:YAG ns-laser	10 nm	spherical colloidal particles
	λ = 532, 1064 nm; 60 ns; 100 Hz, 575 mJ·cm−2					

16	Au_7_Fe_27_ metal plate	ethanol	AuFe alloy	Nd:YAG quantel YG981E-laser	30–60 nm	crystalline NPs
	λ = 1064 nm; 9 ns; 10 Hz, 3.0 J·cm^−2^					

17	Au-plates; raw Fe_2_O_3_ NPs	ethanol	AuFe alloy	Nd:YAG laser	400–500 nm	submicrometer spherical NPs
	λ = 355 nm; 7 ns; 10 Hz, 150 mJ·cm^−2^					

18	PtIr target	acetone	PtIr alloy	femtosecond laser	10–66 nm	spherical NPs
	λ = 800 nm; 120 fs; 5 Hz; 300 μJ; focal length 150 mm					

19	Ni_58.4_Ti_41.6_ target	water	NiTi alloy	femtosecond laser	100–200 nm	NPs
	λ = 1030 nm; 300 fs; 200 kHz; 8.5 μJ; focal length 3 mm					

Laser-generated NPs exhibit exceptional light absorption and utilization capabilities, which is a primary advantage of these materials. For instance, laser-synthesized TiO_2_ NPs are known to have a narrower bandgap, allowing them to capture a broader range of the solar spectrum, including visible and even near-infrared wavelengths [219–223]. Similarly, CdS particles produced through laser techniques have demonstrated enhanced light-harvesting efficiency, improving their photocatalytic activity and enabling these materials to drive a wide variety of energy-driven chemical processes more effectively [224–225]. The ability to precisely control the physical and electronic structure of the laser-generated particles allows for the tailoring of their optical absorption, charge carrier dynamics, and surface reactivity [214]. Specifically, laser-synthesized TiO_2_ particles have shown to have a 50% enhancement in visible light absorption and a 25% increase in active surface sites compared to traditionally synthesized photocatalysts [226]. This remarkable level of control is the key to unlocking the full potential of these materials, as it enables the design of photocatalysts with enhanced light harvesting capabilities, efficient charge separation and migration, and increased exposure of active sites [206,227–228].

The optimization of charge carrier dynamics is a critical aspect of photocatalysis, and laser-synthesized particles play a vital role in this area. In conventional photocatalytic materials, the recombination of electron–hole pairs can significantly hinder efficiency, as these charge carriers recombine before they have the opportunity to participate in the desired chemical reactions [218,229]. Laser-generated particles have demonstrated improved charge separation and reduced recombination rates [214,217,230–233]. By carefully controlling the laser synthesis parameters, it is possible to create particles with fewer defects and grain boundaries, which act as recombination centers [7,92,211,214,232,234–235]. For example, laser-synthesized TiO_2_ NPs have shown a 40% reduction in electron–hole recombination rate and a 30% increase in charge carrier lifetime [236]. Laser-generated CdS particles have also exhibited improvements in charge separation efficiency and charge carrier mobility, owing to the ability to precisely control their physical and electronic structure [225]. Laser-generated particles can also be designed to promote efficient charge transport, with the use of co-catalysts and surface modifications to further enhance charge separation and reduce recombination [92,217,237–238].

The advantages of laser-synthesized particles extend beyond optical properties and charge carrier dynamics, as these materials also exhibit exceptional surface reactivity and active site engineering [239]. By manipulating the laser synthesis parameters, researchers can create materials with tailored surface areas, pore sizes, and chemical compositions, all of which play a key role in determining the rate and selectivity of photocatalytic reactions. Laser-generated particles can also be designed with controlled surface terminations and defect structures, which can act as active sites for specific reactions. For example, laser-synthesized TiO_2_ NPs have shown a 25% increase in active surface sites and a 35% enhancement in adsorption capacity of organic pollutants [240]. Furthermore, CdS particles have demonstrated improved surface reactivity due to the presence of controlled surface defects and the ability to engineer specific surface facets by laser processing [241].

In addition to their superior optical and electronic properties, laser-generated particles often exhibit improved stability and durability compared to their conventionally synthesized counterparts. This enhanced stability and robustness can be attributed to the precise control afforded by laser synthesis techniques, which allow for careful engineering of the particles’ physical and chemical structure [232]. Laser-generated TiO_2_ NPs have demonstrated enhanced resistance to photocorrosion and longer operational lifetimes, making them more suitable for practical applications in harsh environments [242]. They also possess a more defective crystalline structure and thus a higher degree of surface passivation, which contribute to their superior resistance to degradation processes like photocorrosion [243]. Additionally, the aforementioned ability to fine-tune the particle size and morphology through laser synthesis has led to the development of nanostructures that are less susceptible to mechanical and thermal stresses, further enhancing the durability and operational longevity of these photocatalysts [244].

In the field of water remediation, laser-synthesized particles have demonstrated remarkable efficacy in the degradation and complete mineralization of a diverse array of organic pollutants, including recalcitrant dyes, persistent pharmaceuticals, and complex industrial effluents [245]. Studies have shown that laser-generated TiO_2_ particles can achieve up to 90% degradation of organic dyes under visible light irradiation, outperforming conventional TiO_2_ by 30% [222]. SnS thin films synthesized using laser-fragmented/ablated colloid also exhibited 95% methylene blue degradation in 120 min [107]. Additionally, CdS particles have been shown to degrade over 80% of pharmaceutical contaminants in water within 2 h, a 25% improvement compared to their non-laser-generated counterparts [245]. Their ability to efficiently harness visible light energy and generate highly reactive oxygen species, such as superoxide radicals and hydroxyl radicals, has made them invaluable tools in the quest for sustainable and environmentally friendly water treatment solutions [246]. These photocatalytic materials can effectively break down and mineralize a wide spectrum of organic contaminants, transforming them into benign, inorganic end products, thereby restoring the quality of water resources and minimizing the impact of these pollutants on aquatic ecosystems [227].

Beyond water treatment, the laser-processed photocatalysts have also found promising applications in the production of renewable fuels and valuable chemicals [207,227,240,243,247–248]. The efficient light absorption and charge separation exhibited by these materials have enabled the selective conversion of carbon dioxide and water into hydrogen, methane, and other hydrocarbon fuels, contributing to the development of sustainable energy alternatives [249]. Studies have shown that laser-generated TiO_2_ photocatalysts can achieve up to 30% conversion efficiency in the photoelectrochemical reduction of CO_2_ to methane [231]. The exceptional properties of laser-synthesized particles have also demonstrated promising applications in the mitigation of air pollutants [245–246,250–254]. These highly customizable photocatalysts have exhibited outstanding performance in the photocatalytic degradation and complete mineralization of a wide range of airborne organic contaminants, encompassing volatile organic compounds and nitrogen oxides [246]. Studies have shown that laser-synthesized particles exhibit an increased exposure of active surface sites, leading to a 25% enhancement in the photocatalytic reduction of NO*_x_* under visible light irradiation [255]. This improved performance can be attributed to the efficient generation and transfer of charge carriers, as well as the greater availability of active sites for the adsorption and subsequent oxidation or reduction of air pollutants [255–261]. As research in this field progresses, the widespread adoption of these innovative materials could drive significant advancements in air quality and the development of sustainable solutions to address the growing global challenge of atmospheric pollution. The tailored properties of laser-synthesized particles have opened up new avenues in organic synthesis, allowing for the selective transformation of complex organic compounds through precisely controlled redox reactions [211,262]. Also, laser-processed photocatalysts have exhibited remarkable selectivity and efficiency in the production of valuable specialty chemicals and pharmaceuticals, surpassing the performance of traditional catalysts. For instance, researchers have reported the selective oxidation of benzyl alcohol to benzaldehyde with over 90% yield using laser-generated CdS photocatalysts, highlighting their potential for the synthesis of specialty organic compounds [263]. The ability to fine-tune the electronic and structural properties of these materials through laser-based techniques enables the design of photocatalysts that can selectively activate specific chemical bonds, leading to enhanced reaction rates, improved product selectivity, and reduced waste generation [214].

While the potential of laser-generated particles in photocatalysis is undeniable, there are still several obstacles that need to be addressed to facilitate their large-scale implementation. These include the need for further optimization of photocatalyst synthesis and scale-up, the development of efficient photoreactor designs, and the improvement of long-term photocatalyst stability and reusability [223]. One of the key challenges in the widespread adoption of laser-generated photocatalysts is the scalability of their synthesis processes. Current laboratory-scale methods may not be readily translatable to industrial-scale production, requiring the development of more cost-effective and high-throughput synthesis techniques [264]. Additionally, the precise control over particle size, morphology, and composition, which are critical for optimal photocatalytic performance, can be challenging to maintain at larger scales [265]. In addition to the synthesis challenges, the design of efficient photoreactor systems for practical applications is another area that requires further research and development. Factors such as effective light delivery, efficient mixing, and the management of heat and mass transfer processes need to be carefully considered to maximize the utilization of the photocatalysts [206]. Furthermore, the long-term stability and reusability of laser-generated photocatalysts is an important consideration for their real-world implementation [214]. Photocatalytic materials can sometimes suffer from deactivation or degradation during prolonged operation, reducing their efficiency and lifespan [266]. Strategies to improve the structural and chemical stability of the laser-generated particles, such as the use of protective coatings or the development of self-healing mechanisms, can enhance their durability and increase their practical applicability [267]. The potential for photocorrosion, deactivation, or fouling of the catalysts over extended periods of use must be addressed to ensure the sustainability and cost-effectiveness of the photocatalytic processes. Likewise, the integration of laser-generated photocatalysts into practical systems and the optimization of reactor designs and operation conditions remain active areas of research [212]. Addressing issues related to mass transfer, light distribution, and catalyst recovery and reuse is crucial for the successful deployment of these materials in large-scale applications [268].

Despite these challenges, the properties of laser-generated particles, such as their high surface area, tunable electronic structure, and remarkable light-harvesting capabilities, offer significant opportunities for further optimization and innovation. Ongoing research efforts are focused on addressing these challenges through various strategies, such as the development of more robust and stable photocatalyst designs, the integration of co-catalysts or heterojunction structures to enhance charge separation and transfer, and the implementation of advanced reactor configurations to improve light utilization and reaction kinetics. As these advancements continue, the impact of laser-generated particles on the field of photocatalysis could be truly transformative. To further harness the full potential of laser-generated photocatalysts, researchers have explored various strategies to enhance their overall performance and applicability.

One key approach is the rational design and engineering of the photocatalyst’s structure and composition. The incorporation of metal or semiconductor heterostructures onto the surface of laser-generated particles has been shown to improve charge separation and transfer, leading to enhanced photocatalytic efficiency [91]. It has been reported that the incorporation of noble metal co-catalysts, such as platinum or gold, can improve charge separation and enhance the photocatalytic performance of laser-generated particles by up to 40% under visible light irradiation [269]. Additionally, the controlled introduction of defects or dopants into the crystal lattice of laser-generated particles can modulate their band structure and improve their visible light absorption, further expanding the range of usable solar energy [227]. Kanakillam et al. have reported an improvement by 82% in visible light phototcatalytic degradation of methylene blue dye using laser-ablated bimetallic Ag/Au nanoparticles incorporated in ZnO [125]. Another strategy involves the judicious combination of laser-generated photocatalysts with complementary materials, such as co-catalysts or support structures [270]. The integration of laser-generated particles with other functional components, such as plasmonic nanostructures or carbon-based materials, can synergistically enhance light harvesting, charge transport, and surface catalytic activity. For example, the coupling of laser-generated TiO_2_ particles with MOFs has been shown to improve the adsorption and activation of CO_2_, leading to enhanced photocatalytic conversion rates [271].

Furthermore, the development of efficient reactor designs and reaction engineering approaches has played a crucial role in maximizing the practical applicability of laser-generated photocatalysts [214]. Careful consideration of factors such as reactor geometry, light distribution, fluid dynamics, and mass transfer processes can help to optimize the utilization of the photocatalyst and the overall productivity of the system. Research shows that the use of improved reactor configurations, such as fluidized bed reactors or membrane reactors, can enhance the mass transfer and light accessibility of the laser-generated photocatalysts, leading to improved overall performance [214]. Experiments have demonstrated that the use of advanced photoreactor designs can increase the overall photocatalytic activity by 25–30% compared to conventional batch reactors [272]. Thus, we see that the use of laser-generated particles for photocatalytic applications offers a promising path toward addressing global challenges in the fields of energy and environmental remediation. By combining the unique properties of laser-generated materials with innovative strategies for enhancing their performance and scalability, researchers are paving the way for the widespread adoption of this technology.

Laser-synthesized particles stand out in photocatalysis with 20–50% increase in light absorption, enhanced charge separation efficiency, and greater surface reactivity compared to conventional photocatalysts. For instance, laser-generated TiO_2_ has shown up to a 40% reduction in electron–hole recombination and a 30% increase in charge carrier lifetime, resulting in up to 90% degradation of dyes in water compared to chemical TiO_2_. While laser-generated CdS and SnS particles demonstrate over 90% degradation of dyes and significant reductions of nitrogen oxides under visible light. Noble metal/bimetallic NPs incorporated in ZnO nanostructures resulted in 82% methylene blue degradation under visible light. Additionally, laser-synthesized photocatalysts play an important role in renewable energy production by efficiently converting CO_2_ and water into fuels like methane and hydrogen, and they have shown great promise in selective organic dyes achieving high yields and specificity.

#### 3.4 Electrochemical/photoelectrochemical devices

Laser-based synthesis methods have emerged as a versatile and powerful approach for the generation of nanostructured materials with tailored properties for electrochemical energy conversion and storage applications [88,92]. This precise control is a key advantage, making these laser-based synthesis methods attractive and indispensable for the development of high-performance electrocatalysts and photoelectrocatalysts. The ability to fine-tune the nanomaterial characteristics at the atomic and molecular scale can lead to significant improvements in catalytic activity, stability, and selectivity, which are critical for advancing electrochemical energy technologies [88,92]. A review by Zhao et al. provides a comprehensive overview of the laser synthesis and microfabrication techniques for the generation of nanostructured materials with applications in energy conversion and storage [92]. The authors discuss the fundamental mechanisms underlying laser-induced effects, such as melting, plasma formation, and vaporization, and how these can be leveraged to tailor the properties of the resulting nanomaterials. In a complementary review, Lee et al. delve deeper into the specific applications of laser-induced nanomaterials for electrochemical energy storage and conversion devices, highlighting the unique advantages of these techniques in introducing defects, heteroatoms, and heterostructures to enhance electrochemical performance [273]. The advantages of laser-assisted synthesis methods for electrocatalytic and photoelectrocatalytic applications are manifold. For example, laser-induced defects and heteroatom doping can optimize the electronic structure and active site density, leading to enhanced catalytic activity and stability. Additionally, the ability to epitaxially grow nanostructured catalysts on appropriate substrates can further improve their performance and integration into device architectures [92].

In the field of electrochemistry, laser-assisted synthesis methods have been extensively explored for the fabrication of advanced catalysts. For instance, laser-assisted synthesis has been employed to generate hybrid structures comprising metal and metal oxide NPs supported on carbon-based materials, such as graphene and carbon nanotubes. These hybrid catalysts demonstrate enhanced catalytic performance for crucial electrochemical reactions like the oxygen evolution reaction (OER), which is a key step in water splitting and fuel cell technologies. Laser ablation, in particular, has emerged as a versatile approach for creating unique surface morphologies on current collector materials, such as copper foams. The laser-induced micro/nanostructures on the copper surface can serve as efficient platforms for the deposition of electrocatalysts, further improving the overall performance and integration of the catalytic systems. The ability to fine-tune the surface characteristics of the current collectors through laser processing enables the optimization of catalyst–support interactions, leading to enhanced catalytic activity and stability [274]. Furthermore, the versatility of laser-assisted synthesis methods extends to the development of photoelectrocatalytic devices. These techniques allow for the fabrication of integrated photoelectrodes with tailored light-absorbing and charge-transport properties, which are crucial for efficient solar-to-fuel conversion.

The exceptional versatility of laser-based techniques has been a key factor in their widespread adoption for the development of advanced photoelectrochemical materials. These sophisticated methods allow for precise control over the structural and compositional features of the synthesized nanomaterials, including the strategic introduction of defects, heteroatom doping, and the formation of intricate heterojunction architectures. Such fine-tuning of the material properties has been crucial in enhancing their light absorption capabilities, charge separation efficiencies, and overall electrochemical performance [88,92,274]. For instance, the use of laser irradiation has enabled the fabrication of hierarchical nanostructures, such as TiO_2_ nanotube arrays decorated with metal or metal oxide NPs [235]. These hybrid architectures combine the high surface area and efficient charge transport of the nanotube arrays with the enhanced light-harvesting and catalytic properties of the decorative NPs, leading to remarkable improvements in photoelectrochemical hydrogen production and organic pollutant degradation [275].

**Figure 20:**
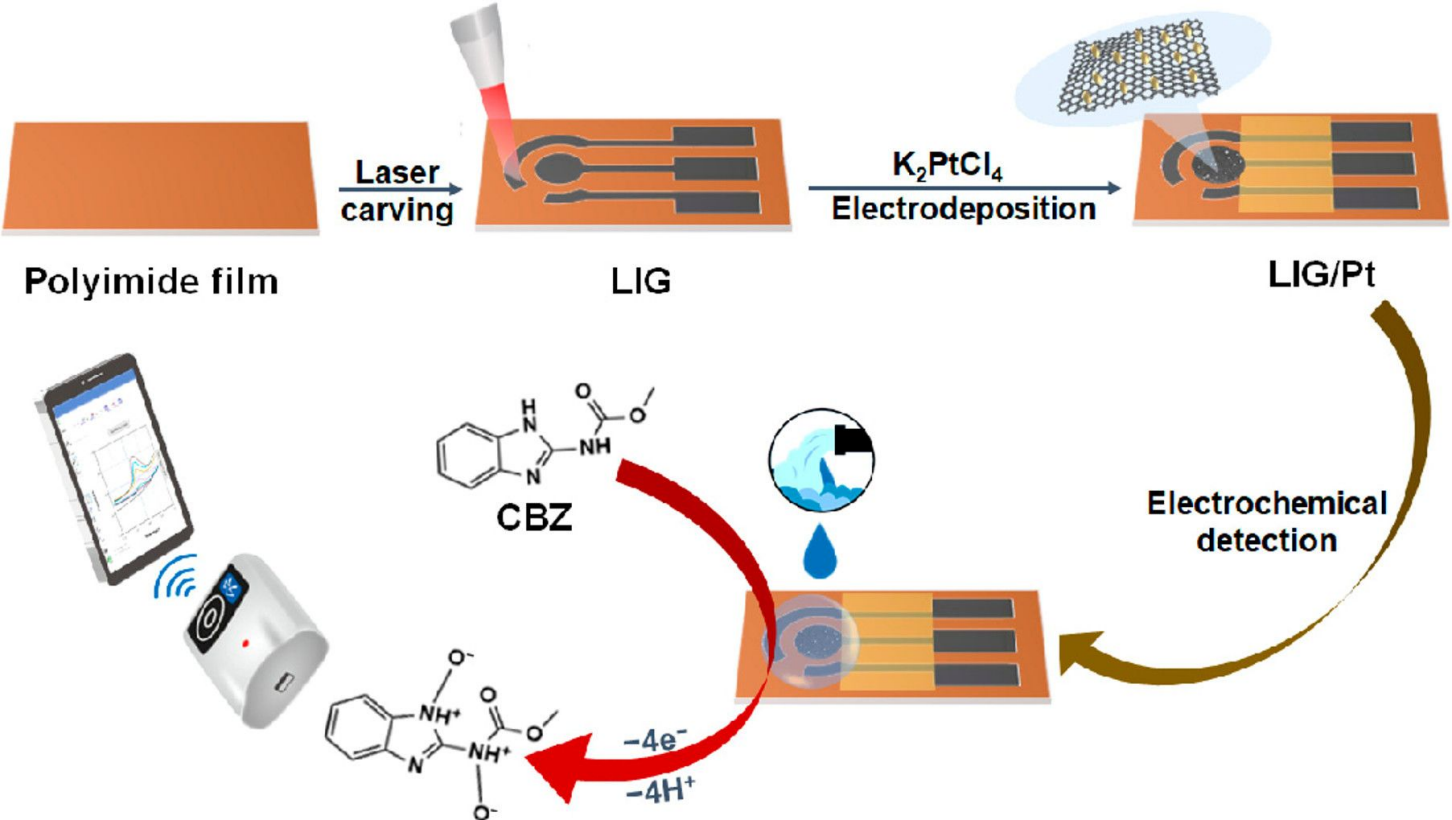
Preparation of LIG/Pt electrochemical sensor and its application for the real-time detection of CBZ in wastewater samples. Figure 20 was reproduced with permission from [277] (© 2023 L. Wang et al., published by MDPI, distributed under the terms of the Creative Commons Attribution 4.0 International License, https://creativecommons.org/licenses/by/4.0).

**Figure 21:**
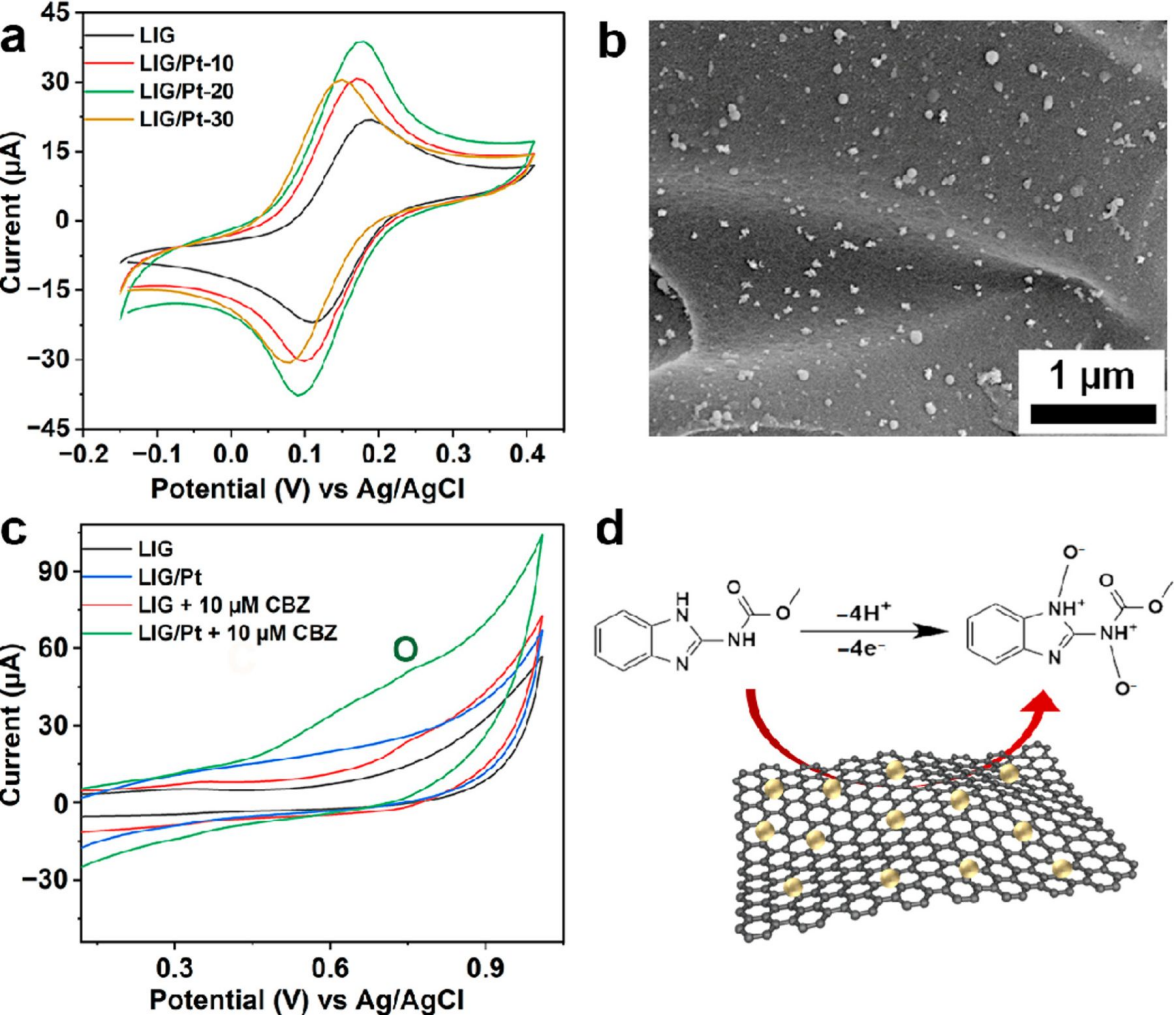
Characterization of the prepared LIG/Pt sensor. (a) Optimization of the electrodeposition cycles of the LIG/Pt sensor. (b) SEM image of the LIG/Pt sensor under optimal conditions. (c) CV responses of the LIG/Pt and bare LIG with or without 10 µΜ CBZ. (d) The electrochemical detection mechanism of CBZ. Figure 21 was reproduced from [277] (© 2023 L. Wang et al., published by MDPI, distributed under the terms of the Creative Commons Attribution 4.0 International License, https://creativecommons.org/licenses/by/4.0).

The successful development of laser-assisted synthesis methods for electrocatalytic and photoelectrocatalytic devices relies on a comprehensive understanding of the underlying mechanisms governing the laser-material interactions and the subsequent formation of the desired nanostructures [88,92,274]. Factors such as the laser wavelength, fluence, pulse duration, as well as the physicochemical properties of the precursor materials, play a crucial role in determining the final morphology, composition, and catalytic performance of the fabricated devices [274]. The absorption of laser energy by the precursor materials can induce various effects, including melting, plasma formation, and vaporization, which dictate the characteristics of the resulting nanomaterials. Furthermore, the ability to epitaxially grow nanostructured catalysts on appropriate substrates can further improve their performance and integration into device architectures [92]. Recent studies have further highlighted the remarkable versatility of laser-assisted synthesis methods in the development of highly efficient electrocatalysts and photoelectrocatalysts. For instance, the strategic introduction of laser-induced grain boundaries in ruthenium NPs has been demonstrated as an effective strategy to significantly boost the OER activity [276]. This enhancement is attributed to the formation of structural defects that modulate the electronic structure and increase the density of catalytically active sites. Extending this concept, Figure 20 illustrates the fabrication and application of a laser-induced graphene(LIG)/platinum electrochemical sensor for the real-time detection of the pharmaceutical compound carbamazepine in wastewater. The process involves laser carving of a polyimide film to produce conductive LIG patterns, followed by platinum electrodeposition using potassium tetrachloroplatinate as the precursor. The resulting LIG/Pt sensor facilitates electrochemical detection of carbendazim (CBZ) via redox reactions, with the output data wirelessly transmitted to a smartphone for on-site analysis. This example highlights the adaptability of laser-induced functionalization techniques for environmental sensing applications. Further validating the sensor’s performance, Figure 21 presents its structural and electrochemical characterization. Electrochemical analyses demonstrate enhanced sensing performance following platinum deposition, while SEM imaging reveals a uniform and well-defined surface morphology. The LIG/Pt sensor exhibits a markedly stronger response to CBZ compared to bare LIG, indicating improved sensitivity. A simplified reaction mechanism is also provided to illustrate the redox-based detection pathway. These findings collectively emphasize the potential of laser-assisted synthesis for precise control over composition, morphology, and atomic-scale structure, attributes that are crucial for advancing the catalytic activity, durability, and device-level integration of next-generation electrocatalytic and photoelectrocatalytic systems.

Furthermore, the remarkable versatility of laser-based synthesis techniques has enabled the fabrication of sophisticated heterostructured materials for advanced photoelectrocatalytic applications. For example, studies have reported the use of laser-assisted methods to create core–shell nanostructures composed of a semiconductor core and a co-catalyst shell, which have demonstrated enhanced photocatalytic hydrogen production compared to their individual components [278]. Similarly, laser-induced formation of hierarchical TiO_2_ nanotube arrays decorated with metal oxide NPs has been shown to improve light harvesting and charge separation, leading to superior photoelectrochemical performance for organic pollutant degradation [279]. These complex architectures, strategically engineered using laser-based techniques, have been crucial in enhancing the efficiency of solar-to-fuel conversion reactions and the effective removal of organic pollutants in photoelectrocatalytic devices. The ability to precisely control the structural and compositional features of these heterostructures using laser processing is a key advantage, as demonstrated in several reported articles. For instance, a study by Wang et al. utilized laser-induced defects and heteroatom doping in TiO_2_ nanotubes to enhance light harvesting and charge separation, leading to improved photoelectrochemical performance for organic pollutant degradation [280]. Similarly, Zhou et al. reported the use of femtosecond laser ablation to create intricate micro/nanostructured copper foam surfaces, which, when combined with the subsequent growth of cobalt hydroxide nanosheets, exhibited exceptional activity and stability for the OER [274]. The tailored design of the heterointerfaces allows for the effective separation of photogenerated charge carriers, facilitating their transport to the catalyst surface and improving the efficiency of solar-to-fuel conversion reactions. Additionally, the intricate hierarchical arrangements can enhance the accessibility and transport of reactants and products, further optimizing the overall catalytic performance [281].

The implementation of these laser-fabricated heterostructured materials has led to significant advancements in photoelectrocatalytic technologies, enabling enhanced solar energy harvesting, improved fuel production, and the effective removal of organic pollutants [282]. The remarkable versatility of laser-based synthesis methods has been crucial in unlocking the full potential of these sophisticated photoelectrocatalytic architectures. These advanced materials and techniques have further expanded the possibilities for efficient solar energy conversion, clean fuel generation, and environmental remediation through photoelectrocatalytic processes [276]. The ability of laser-assisted synthesis techniques to precisely control the structural and compositional features of these materials has been a key driver in amplifying the performance of electrochemical and photoelectrochemical devices. The tailored design of the heterointerfaces and hierarchical nanostructures using laser processing has facilitated better charge separation and transport, resulting in enhanced solar-to-fuel conversion efficiencies and more effective pollutant degradation [283]. Furthermore, the strategic introduction of defects, doping, and complex heterostructures through laser-based methods has unlocked new levels of catalytic activity and stability, pushing the boundaries of what is possible with these advanced electrochemical and photoelectrochemical systems [85,88,92,284–286]. By carefully manipulating the laser parameters, researchers can induce desirable effects such as melting, plasma formation, and vaporization, which dictate the final morphology, composition, and catalytic performance of the fabricated devices [92,287–289]. The ability to epitaxially grow nanostructured catalysts on appropriate substrates can further improve their performance and integration into device architectures. The tailored design of these complex architectures, such as core–shell or hierarchical nanostructures, has been a key factor in enhancing the efficiency of solar-to-fuel conversion reactions and the effective removal of organic pollutants [290–291]. Overall, laser-assisted synthesis methods offer successful development of advanced electrocatalytic and photoelectrocatalytic devices, paving the way for improved energy conversion, storage, and environmental remediation technologies.

Nanomaterials of TiO_2_ stand out in electrochemical due to their chemical stability, non-toxicity, and strong oxidative capabilities. Defect-rich TiO_2_ nanopowders synthesized using pulsed laser ablation have shown promising results in photoelectrochemical CO_2_ conversion, achieving ≈30% efficiency in converting CO_2_ to CH_4_ under light irradiation. Moreover, hierarchical TiO_2_ nanotube arrays decorated with metal oxide NPs, demonstrate significantly enhanced performance in degrading organic pollutants compared to bare TiO_2_ nanotubes. These hierarchical architectures benefit from the combination of high surface area, improved light absorption, and efficient charge transport pathways.

Sulfur-based materials, particularly CdS combined with catalysts like MoS_2_, as promising systems for enhancing photocatalytic hydrogen generation. Specifically, core–shell structures (e.g., CdS@MoS_2_), synthesized or deposited using laser-based techniques, demonstrate superior hydrogen production compared to their individual components. This improvement arises from the favorable band alignment between the sulfide semiconductor core and the co-catalyst shell, which facilitates efficient light absorption in the visible range, effective charge separation at the interface, and accelerated catalytic reactions for solar-to-fuel conversion.

Layered materials like MoS_2_, WS_2_, and graphene, as well as metal NPs decorated two-dimensional (2D) materials play an important role in advancing photoelectrochemical applications. Their unique 2D architecture provides large specific surface areas, tunable electronic properties, and abundant edge sites, all of which are highly beneficial for catalytic reactions such as the hydrogen evolution reaction (HER) and OER. Laser-assisted methods are effectively used to synthesize or modify these layered materials, particularly in the context of core–shell systems like CdS@MoS_2_, where the MoS_2_ shell serves as a highly active catalytic layer for hydrogen generation. The layered nature of MoS_2_ offers significant advantages. Its basal planes are generally inert, but its edge sites are catalytically active, and laser processing can further enhance these active edges or even induce phase transitions. Layered structures can form heterojunctions with semiconductor substrates (such as CdS or TiO_2_), promotes effective charge separation and suppresses recombination, which is critical for maximizing PEC efficiency.

#### 3.5 Hydrogen evolution reaction

The HER is a crucial electrochemical process that involves the reduction of protons to produce H_2_. This process is of great importance in various applications, including energy storage, fuel cells, and sustainable hydrogen production [292–294]. The mechanism of the HER can be broadly divided into three steps, that is, adsorption of protons onto the catalyst surface, electron transfer to the adsorbed protons, and desorption of the resulting hydrogen molecules [295].

The adsorption of protons onto the catalyst surface is the crucial first step in the HER. This step involves the complex interaction between the protons in the electrolyte solution and the active sites on the catalyst surface. The specific nature of the catalyst material, including its surface composition, structure, and electronic properties, can significantly influence the adsorption of protons [296]. Additionally, the pH of the surrounding solution plays a key role in determining the concentration and charge state of the protons, thereby affecting their ability to interact with and adsorb onto the catalyst surface. These factors collectively govern the efficiency and kinetics of the initial proton adsorption step, which in turn impacts the overall performance of the HER. Optimizing the catalyst surface and the reaction conditions to facilitate the proton adsorption process is an active area of research to enhance the overall catalytic activity and hydrogen production rates [297].

The next key step in the HER mechanism is the electron transfer to the adsorbed protons. This electron transfer process is central to the overall efficiency of the reaction, as it directly determines the rate of hydrogen production. The kinetics of this electron transfer step are influenced by a variety of factors, including the electronic properties of the catalyst material, the applied overpotential, and the specific reaction conditions [297]. The catalyst’s electronic structure and its ability to facilitate the transfer of electrons to the adsorbed protons play a pivotal role in governing the kinetics of this step. Factors such as the catalyst’s electronic band structure, the presence of localized d orbitals, and the ability to mediate charge transfer can significantly impact the rate and ease of the electron transfer process. Additionally, the applied overpotential, which is the voltage difference between the equilibrium potential and the operating potential, can also influence the driving force and kinetics of the electron transfer step [298]. The surrounding reaction conditions, such as the pH, temperature, and the composition of the electrolyte solution, can further modulate the electron transfer kinetics. These factors can affect the proton concentration, the solvation of the protons, and the overall thermodynamics and kinetics of the electron transfer process. Optimizing these parameters is crucial for enhancing the efficiency and rate of the HER [297].

The final step in the HER is the desorption of the hydrogen atoms from the catalyst surface, which is a crucial step that ultimately leads to the formation of molecular hydrogen. The ease and kinetics of this hydrogen desorption process are significantly influenced by the catalyst’s surface properties, such as the binding strength between the adsorbed hydrogen atoms and the catalyst surface, as well as the reaction conditions, including the pH, temperature, and applied potential [293]. The efficient desorption of hydrogen molecules from the catalyst surface is essential for maintaining the overall catalytic activity and preventing catalyst poisoning, as it allows for the continuous regeneration of the active sites for the proton adsorption and reduction steps [299]. Optimizing the catalyst’s surface structure and composition to facilitate the hydrogen desorption step is an active area of research in the development of highly efficient and stable electrocatalysts for the HER.

The development of efficient electrocatalysts for the HER has been a significant area of advancement. Platinum was recognized as a highly active catalyst, but its cost limited widespread adoption [300]. In subsequent decades, researchers explored alternative materials to enhance activity, stability, and cost-effectiveness. A breakthrough occurred in the 1970s with the discovery of transition metal-based catalysts, which demonstrated improved performance and efficiency compared to platinum [301]. Their lower cost also made them more viable for large-scale hydrogen production [302]. Researchers have continued to optimize and develop these transition metal-based catalysts, including through the incorporation of support materials and surface property tuning.

These advancements have led to significant enhancements in the catalytic activity, stability, and cost-effectiveness of the HER catalysts, paving the way for their greater adoption and implementation in sustainable hydrogen production technologies [297]. More recently, the emergence of nanostructured materials, such as carbon-based supports and MOFs, has opened new and promising avenues for enhancing the performance of hydrogen evolution catalysts. These advanced materials offer a range of beneficial properties, including high surface areas, tunable pore structures, and the ability to integrate multiple active components [296]. These characteristics have led to remarkable improvements in the catalytic activity and stability of hydrogen evolution electrocatalysts. For instance, graphene-supported transition metal catalysts have demonstrated exceptional hydrogen evolution efficiencies [297]. The large surface area and excellent electrical conductivity of graphene provide an ideal support for the transition metal active sites, facilitating efficient electron transfer and enhancing the overall catalytic performance [303]. Similarly, MOF-derived electrocatalysts have shown great promise, as the unique porous structure and the integration of multiple active components within the framework can synergistically enhance the HER [304]. These nanostructured materials have opened up new design possibilities for the development of highly active, stable, and cost-effective electrocatalysts for sustainable hydrogen production [305].

HER faces several significant challenges that must be addressed to enable the widespread adoption of hydrogen-based energy technologies [296–297]. One of the primary challenges is the development of highly efficient, stable, and cost-effective electrocatalysts that can facilitate the HER under practical operating conditions [297]. The efficiency and kinetics of the HER are heavily dependent on the properties of the catalyst material, including its composition, surface structure, and electronic characteristics [306]. Optimizing these parameters is crucial for enhancing the overall performance and rate of the HER. Researchers have made significant advancements in the field of HER catalysts, exploring strategies such as the development of non-precious metal-based materials, the incorporation of support materials to enhance catalytic properties, and the engineering of nanostructured architectures to maximize active sites and improve mass transport [296–297].

**Figure 22:**
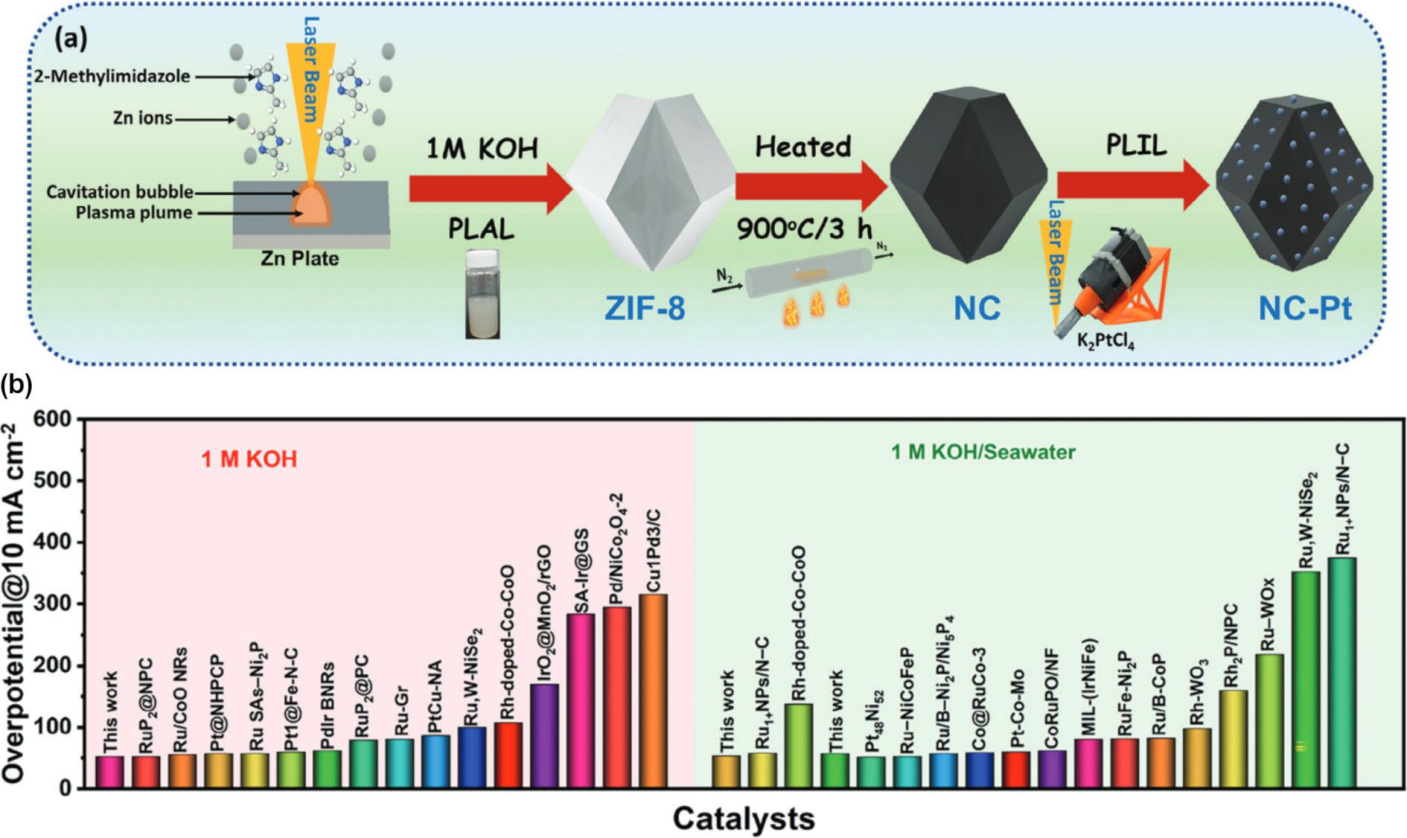
(a) Schematic diagram of the synthesis of NC decorated with Pt nanoclusters using the pulsed laser technique. (b) Comparison of HER performance of the NC-Pt-4 catalyst with recently reported catalysts in various electrolytes. Figure 22 was reproduced from [310], V. Maheskumar et al., “Accelerating the Hydrogen Evolution Kinetics with a Pulsed Laser–Synthesized Platinum Nanocluster–Decorated Nitrogen-Doped Carbon Electrocatalyst for Alkaline Seawater Electrolysis”, *Small*, with permission from John Wiley and Sons. Copyright © 2024 Wiley-VCH GmbH. This content is not subject to CC BY 4.0.

One promising approach to addressing the challenges in the HER is the use of laser-assisted synthesis techniques. These advanced fabrication methods have shown great potential for developing highly efficient and stable HER electrocatalysts. For instance, laser-induced partial reduction and doping of transition metal-based catalysts like FeCoCrNi alloy film with highly oxidized Ni^4+^ species can enhance their catalytic activity by modifying the electronic structure and creating more active sites on the catalyst surface [307]. The precise control over the catalyst’s surface morphology and composition enabled by these laser-based techniques can be leveraged to optimize the adsorption and desorption kinetics of hydrogen, which are critical to the overall efficiency of the HER process [308]. The scalable and high-throughput nature of laser-based synthesis approaches is a critical factor in the cost-effective production of advanced HER electrocatalysts, facilitating their broader adoption in sustainable hydrogen energy technologies. Figure 22 demonstrates this potential by illustrating the synthesis of nitrogen-doped carbon-supported platinum nanoclusters using pulsed laser techniques. The process begins with a ZIF-8 precursor and culminates in the decoration of the carbon matrix with Pt nanoclusters. The figure also highlights the exceptional catalytic performance of the NC-Pt-4 catalyst, which exhibits competitive HER activity in both 1 M KOH and seawater, underscoring its practical applicability across diverse environments. This example further reinforces how laser-assisted synthesis enables precise nanostructuring and compositional tuning, resulting in catalysts with superior electrocatalytic properties. In addition to Pt-based systems, laser-enabled fabrication of nanostructured materials, such as carbon-supported metal catalysts like Mo_2_C NPs encapsulated within nitrogen and phosphorus co-doped carbon shells, has shown promise in enhancing HER performance. These nanostructures exhibit improved mass transport and charge transfer characteristics, attributable to their optimized morphology and electronic configuration, which are directly influenced by laser processing parameters. Collectively, these advancements highlight the pivotal role of laser-based strategies in the rational design and scalable production of next-generation HER electrocatalysts [296]. The unique architecture and the synergistic integration of multiple active components within these nanostructured catalysts can significantly boost the catalytic activity and stability compared to more conventional catalyst designs [309].

**Figure 23:**
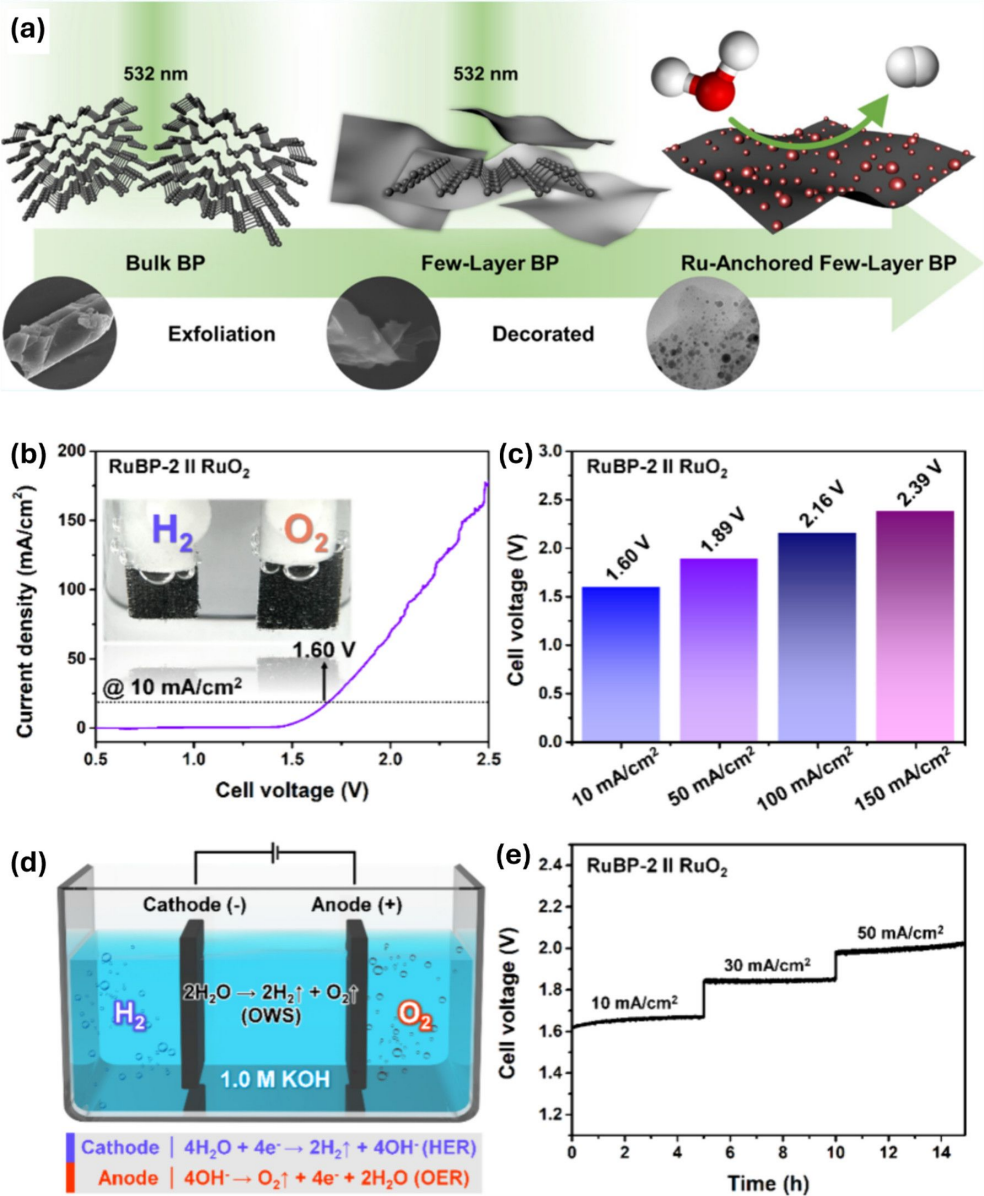
(a) Laser-synthesized Ru-anchored few-layer black phosphorus for superior hydrogen evolution: Role of acoustic levitation; (b) OWS polarization graph for the assembled membrane-less RuO_2_||RuBP-2 electrolyzer; (c) RuO_2_||RuBP-2 cell voltage requires to deliver 10, 50, 100, and 150 mA/cm^2^; (d) schematic of reaction mechanism involved in the OWS process; (e) long-term continuous electrolysis over RuO_2_||RuBP-2 electrodes. Figure 23 was adapted with permission from [316], Copyright (2024) American Chemical Society. This content is not subject to CC BY 4.0.

By making use of the precise control and customization enabled by laser-based techniques, researchers can continue to push the boundaries of HER catalyst design and unlock new possibilities for efficient and cost-effective hydrogen generation [311]. For instance, laser-induced partial reduction and doping of transition metal-based catalysts have been shown to enhance their catalytic activity for the HER. A recent study reported that laser-treated Pt–Ni alloy catalysts exhibited a 34% increase in HER activity compared to their untreated counterparts, with a Tafel slope of 29 mV/dec and an exchange current density of 1.02 mA/cm^2^ [312]. Similarly, laser-synthesized MoS_2_ nanosheets decorated with Pt NPs demonstrated a 2.5-fold improvement in HER activity over commercial Pt/C, achieving a current density of 10 mA/cm^2^ at an overpotential of just 120 mV [313]. Another study demonstrated that laser-fabricated Ni–Mo alloy NPs supported on reduced graphene oxide exhibited outstanding HER activity, achieving a Tafel slope of 35 mV/dec and a current density of 10 mA/cm^2^ at an overpotential of only 110 mV [314]. Similarly, the laser-induced synthesis of cobalt phosphate nanosheets resulted in high HER performance, with a Tafel slope of 45 mV/dec and a current density of 10 mA/cm^2^ at an overpotential of 140 mV. These findings underscore the efficacy of laser-based techniques in tailoring electrocatalyst composition and nanostructure for optimal hydrogen evolution [315]. Figure 23 illustrates the laser-assisted synthesis and water-splitting performance of ruthenium-anchored few-layer black phosphorus. The synthesis process involves the exfoliation of bulk black phosphorus into few-layer nanosheets, followed by laser-mediated decoration with Ru NPs. The resulting electrode architecture is showcased and its application in overall water splitting using RuBP∥RuO_2_ electrodes is demonstrated. Notably, the system requires only 1.6 V to achieve a current density of 10 mA/cm^2^ and maintains stable operation at even higher current densities, confirming its efficiency and robustness. Additionally, the figure includes a schematic of the electrochemical mechanisms underlying both the hydrogen and oxygen evolution reactions in 1.0 M KOH, along with evidence of long-term operational stability during continuous electrolysis. These studies collectively demonstrate the transformative potential of laser-assisted techniques for engineering highly active, durable, and scalable HER electrocatalysts. This is achieved through precise control over their structure, composition, and interface properties.

However, further optimizing the catalyst composition, surface structure, and electronic properties are still needed to push the limits of HER performance of laser-synthesized materials. Additionally, reducing the manufacturing costs of these advanced catalysts remains a challenge for their large-scale implementation in sustainable hydrogen production technologies [294,306,308,311,317].

**Figure 24:**
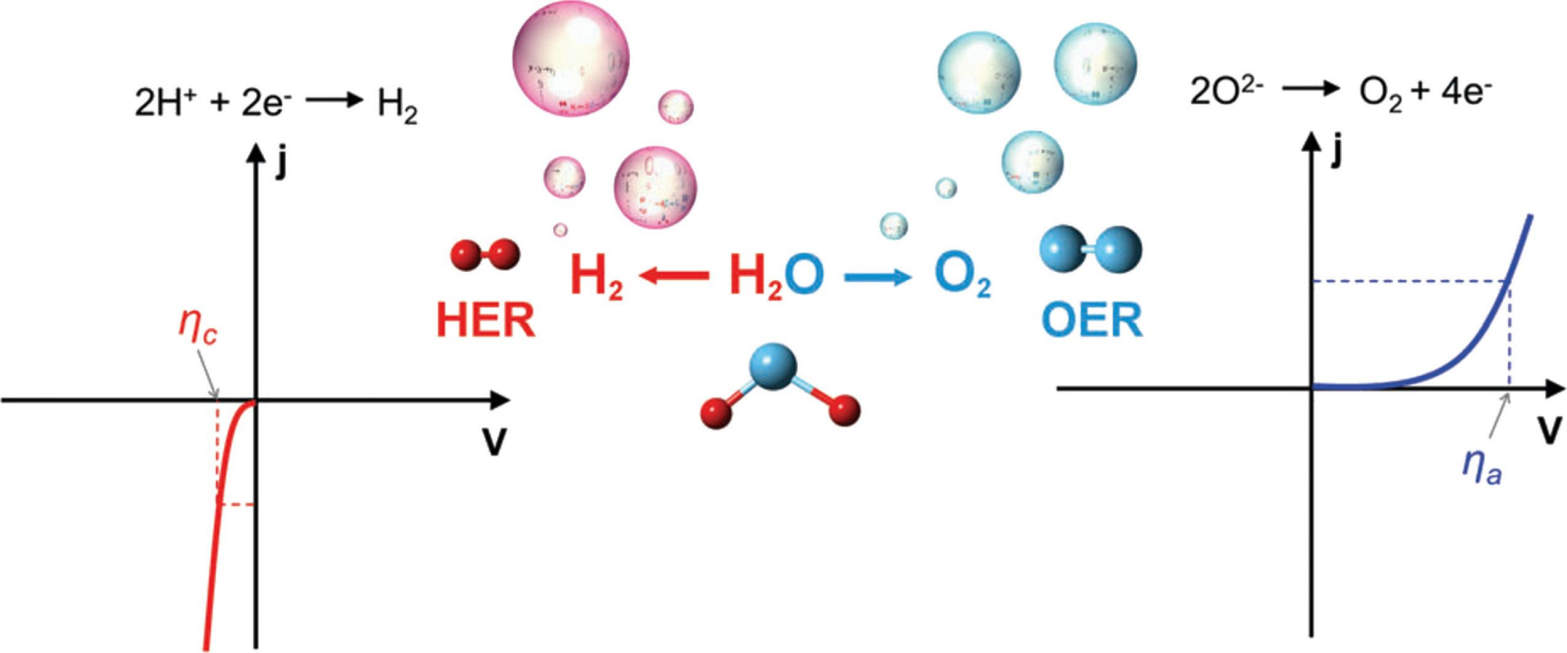
Polarization curves for HER (left) and OER (right). Figure 24 was used with permission of The Royal Society of Chemistry, from [319] (“Electrocatalysis for the oxygen evolution reaction: recent development and future perspectives” by N.-T. Suen et al., *Chem. Soc. Rev.*, vol. 46, issue 2, © 2017); permission conveyed through Copyright Clearance Center, Inc. This content is not subject to CC BY 4.0.

Incorporating support materials, like carbon-based supports or conductive oxides, can improve mass transport and charge transfer properties, leading to enhanced HER performance [308]. Engineering nanostructured architectures, such as core–shell structures or hierarchical designs, can also maximize the number of active sites and optimize the adsorption–desorption kinetics of hydrogen [297]. These approaches, enabled by the precise control offered by laser-based techniques, have the potential to push the limits of HER performance and unlock new possibilities for efficient and cost-effective hydrogen production technologies. These challenges still need to be addressed to fully realize the potential of the HER for large-scale hydrogen production. These include improving the catalytic activity and stability of the electrocatalysts, as well as reducing their manufacturing costs to make them more commercially viable [306,318]. Ongoing research efforts are focused on developing novel catalyst materials, optimizing their surface properties, and exploring innovative reactor designs to enhance the overall efficiency and scalability of the hydrogen evolution process. By addressing these challenges, the HER can become a more viable and compelling option for sustainable hydrogen production, ultimately contributing to the transition toward a clean energy future [297]. Figure 24 complements this discussion by presenting the polarization curves for both the HER and the OER. These curves delineate the overpotential required to achieve specific current densities, with HER occurring at the cathode and OER at the anode during water splitting. The comparative analysis of these polarization profiles not only underscores the performance metrics of existing catalyst systems but also highlights the targets for further improvement in catalyst design and reactor engineering.

#### 3.6 Oxygen evolution reaction

The OER represents a critical electrochemical process that is central to numerous sustainable energy technologies, including water splitting for hydrogen generation, metal–air batteries, and fuel cells [320–321]. This complex reaction, which involves the conversion of water molecules into oxygen gas through a multistep oxidation process, is widely regarded as a significant bottleneck in the advancement of these technologies. This is primarily due to the substantial energy barriers and inherently sluggish kinetics associated with the OER mechanism [322]. The OER mechanism involves the formation of various intermediates and the breaking of strong O–H bonds, which contribute to the overall efficiency and energy requirements of the process. This complexity, coupled with the potential for competing reactions, such as the HER, can further hinder the overall performance and reliability of the devices that rely on the OER [322]. As such, the development of efficient and stable electrocatalysts is a critical research area in the field of sustainable energy, as they can play a crucial role in overcoming the limitations of the OER and enabling the widespread adoption of these technologies.

The rapid population growth and civilization development have led to rising energy demands, environmental pollution, and an energy crisis, presenting a global challenge. Consequently, the exploration of renewable energy has been accelerated to achieve sustainability. Efficient energy conversion, storage, and environmental remediation technologies based on electro- and photochemistry are critical. However, the performance is primarily controlled by the active catalytic materials with diverse working mechanisms [285,321,323]. Laser-generated particles, with their exceptional properties, have the potential to play a crucial role in overcoming the limitations of the OER and enabling the widespread adoption of sustainable energy technologies. A comprehensive understanding of the fundamental principles governing the OER, as well as the specific role of laser-generated particles in enhancing this process, is essential for the continued development and optimization of these technologies.

The OER is a complex, multistep electrochemical process that involves the oxidation of water molecules to generate oxygen gas and protons. This essential reaction is typically represented by the following equation:


[2] $$2{\text{H}}_{2}\text{O}\to {\text{O}}_{2}+4{\text{H}}^{+}+4{\text{e}}^{-}.$$

The OER mechanism can be broadly described by the following steps: (1) adsorption of water molecules on the catalyst surface, (2) formation of *OH and *O intermediates, and (3) oxidation of *O to form O_2_. The specific steps involved in the OER mechanism can vary depending on the catalyst material and the reaction conditions. The efficiency of the OER is largely determined by the ability of the catalyst to facilitate the adsorption and activation of water molecules, as well as the stabilization and subsequent oxidation of the intermediate species. Improving the performance and efficiency of the OER is, therefore, a critical research objective in the field of sustainable energy. The reaction can be further complicated by competing reactions, such as the HER, which can reduce the overall efficiency of the process [324–325].

Laser-generated particles have emerged as a promising class of materials for enhancing the performance of the OER due to their unique physicochemical properties and tunable morphologies [324–325]. These nanostructured materials can be synthesized using a variety of pulsed laser techniques, such as laser ablation, laser pyrolysis, and laser-induced fragmentation, which allow for the precise control of particle size, composition, and surface structure [321,326].

One of the key advantages of laser-generated particles is their high surface area-to-volume ratio, which can significantly increase the number of active sites available for the OER [327]. Additionally, the ability to engineer the particle composition and morphology can lead to the creation of specialized catalysts with enhanced activity, selectivity, and stability. For example, laser-generated metal and metal oxide NPs have demonstrated improved catalytic activity compared to traditional electrocatalysts due to their unique electronic structures and the presence of defects or disorders on the surface [328]. These structural features can facilitate the adsorption and activation of water molecules, as well as the stabilization of the intermediate species involved in the OER mechanism.

The use of laser-generated particles in OER catalysts has also been shown to enhance the stability and durability of the overall system. The ability to finely control the particle size and distribution can help mitigate issues such as agglomeration, which can lead to a loss of active surface area and reduced catalyst performance over time [329]. Furthermore, the versatility of laser-based synthesis techniques allows for the incorporation of multiple elements and the creation of complex heterostructures, which can further optimize the catalytic activity and selectivity of the OER process. Recent studies have demonstrated that laser-generated particles composed of noble metals, transition metals, and carbon-based materials can exhibit exceptional performance in the OER. For example, a highly ordered hierarchically porous carbon (HPC)-based cathode derived from MOFs fabricated using laser-generated particles was found to enable a low cathodic potential of −0.5 V, at which hydrogen evolution could be greatly suppressed, while simultaneously enhancing the buildup of hydrogen peroxide, a key intermediate in the OER [325]. Similarly, the incorporation of laser-generated TiO_2_–Pt composites into photoelectrochemical systems has been shown to significantly improve the efficiency of hydrogen generation through the OER [320]. These materials exhibit a high degree of defects and metastable phases, which can facilitate the adsorption and activation of water molecules, thereby enhancing the kinetics of the OER.

The efficiency and performance of laser-generated particles in catalyzing the OER are determined by an intricate interplay of factors, including the composition, morphology, and surface chemistry of the particles. One of the critical factors determining the efficiency and performance of laser-generated particles in catalyzing the OER is the composition of the particles. Studies have shown that strategically incorporating certain dopants or engineering heterogeneous structures, such as core–shell or alloy configurations, can significantly enhance the catalytic activity of these materials [320–321]. For instance, the inclusion of transition metal ions, such as Fe, Co, or Ni, into the lattice structure of laser-synthesized TiO_2_/ZnO particles has been found to be particularly effective in improving the OER performance. The incorporation of these dopants can modify the electronic structure of the particles, creating additional active sites and altering the adsorption and activation of water molecules on the catalyst surface [321]. This, in turn, can facilitate the multistep OER mechanism, leading to enhanced catalytic activity and efficiency. Similarly, the formation of core–shell or alloy structures, where the laser-generated particles consist of a combination of different metals or metal oxide components, can also result in a significant boost in the OER performance. The synergistic interactions between the different constituents in these heterogeneous structures can further optimize the charge transfer, surface properties, and catalytic active sites, resulting in superior catalytic performance compared to their individual components [320–321].

**Figure 25:**
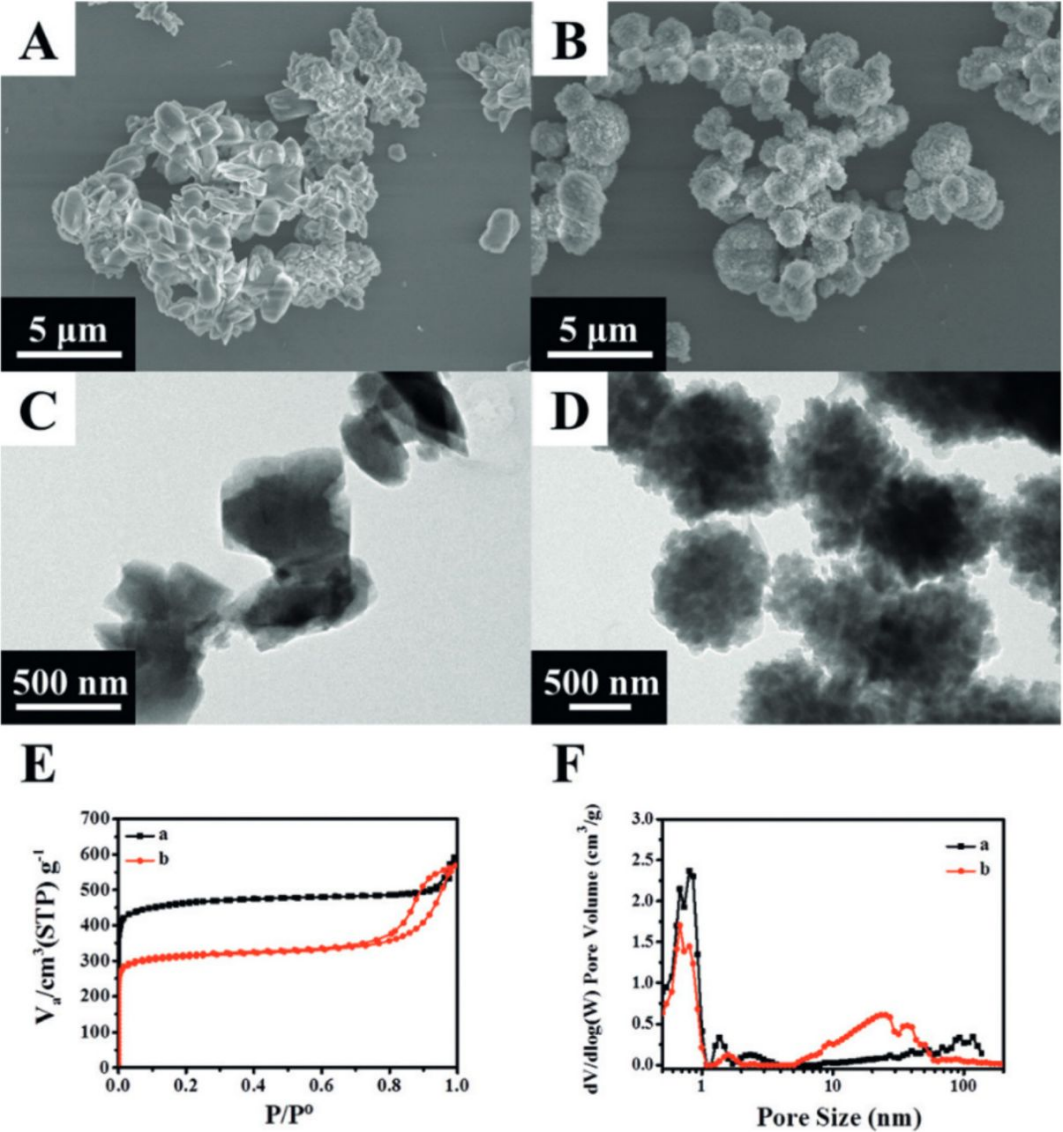
(A, B) FESEM images, (C, D) TEM images, (E) nitrogen absorption-desorption isotherms, (F) pore size distribution of samples. Figure 25 was reproduced from [333], H. Li et al., “Crystal‐Growth‐Dominated Fabrication of Metal–Organic Frameworks with Orderly Distributed Hierarchical Porosity”, *Angewandte Chemie International Edition*, with permission from John Wiley and Sons. Copyright © 2019 WILEY‐VCH Verlag GmbH & Co. KGaA, Weinheim. This content is not subject to CC BY 4.0.

**Figure 26:**
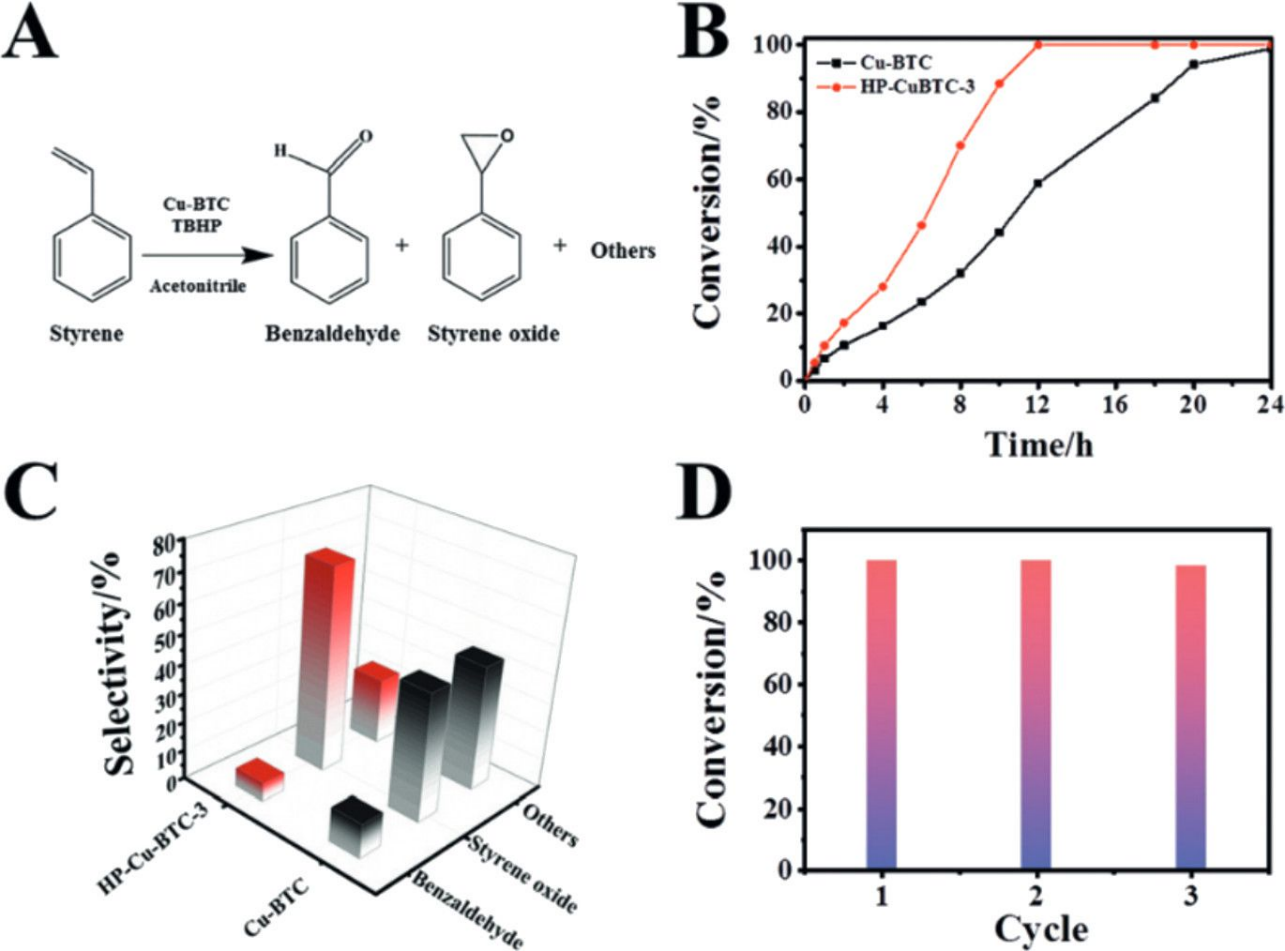
(A) catalytic equation, (B) conversion rate, (C) catalytic selectivity, (D) catalytic stability. Figure 26 was reproduced from [333], H. Li et al., “Crystal‐Growth‐Dominated Fabrication of Metal–Organic Frameworks with Orderly Distributed Hierarchical Porosity”, *Angewandte Chemie International Edition*, with permission from John Wiley and Sons. Copyright © 2019 WILEY‐VCH Verlag GmbH & Co. KGaA, Weinheim. This content is not subject to CC BY 4.0.

The morphology of laser-generated particles is a critical determinant of their catalytic performance for the OER. High surface area, porous structures, and well-controlled exposed crystal facets increase the number of accessible active sites and enhance mass transport within the porous network. For instance, laser-fabricated TiO_2_ particles exhibiting hierarchical nanostructures, such as nanotubes, nanosheets, and nanocubes, have demonstrated superior OER activity compared to their bulk counterparts [330–331]. Figure 25 presents a detailed analysis of the morphology and porosity of two such samples. The FESEM images in Figure 25A,B reveal distinctly different surface architectures, while the TEM views in panels Figure 25C,D offer insights into their internal frameworks. The corresponding nitrogen adsorption–desorption isotherms and pore size distributions in Figure 25E,F highlight the variations in porosity between the two samples. These complex morphologies not only provide an expanded reactive interface but also facilitate the adsorption and activation of water molecules on catalytically favorable crystal facets [332]. Advancing from structural to functional aspects, Figure 26 illustrates the catalytic oxidation performance of a conventional Cu–BTC framework juxtaposed against its hierarchical porous counterpart, HP-CuBTC-3. In Figure 26A, the conversion of styrene into styrene oxide and benzaldehyde is schematically depicted, while Figure 26B tracks the time-dependent conversion rates, clearly evincing heightened efficiency for HP-CuBTC-3. Figure 26C compares product selectivity, with the hierarchical sample favorably biasing the desired epoxide and aldehyde, and Figure 26D demonstrates the catalyst’s stability over three successive reaction cycles. The enhanced diffusion pathways and augmented accessibility of active sites afforded by the hierarchical porosity ultimately culminate in improved OER kinetics and overall catalytic efficiencies, underscoring the potency of laser-assisted structuring in electrocatalyst design [288].

The surface chemistry of laser-generated particles is another crucial factor that can significantly influence the OER. Laser synthesis techniques have the remarkable ability to create particles with unique surface defects, such as oxygen vacancies or metal-rich sites, which can serve as highly active centers for the adsorption and activation of water molecules during the reaction [334]. These surface defects introduce localized electronic states within the bandgap of the material, facilitating the transfer of electrons and protons, which is a crucial step in the multistep oxygen evolution mechanism. Moreover, the presence of surface adsorbates, such as hydroxy groups or organic ligands, can also have a profound impact on the wettability, charge transfer, and overall catalytic properties of the laser-generated particles [280]. These surface species can alter the polarity and hydrophilicity of the particle surface, influencing the adsorption and activation of water molecules [335]. Additionally, the presence of these adsorbates can modify the electronic structure of the particles, either enhancing or hindering the charge transport processes essential for the OER [307].

One of the key mechanisms underlying the enhanced catalytic performance of laser-generated particles in the OER is the improved charge transfer and separation at the particle–electrolyte interface [336]. Laser-synthesized particles often exhibit exceptional electrical conductivity and charge transport properties, which can significantly facilitate the transfer of electrons during the multistep OER [337]. This efficient charge movement is crucial, as the OER involves the transfer of multiple electrons and the formation of various intermediates, each requiring rapid charge transport to drive the overall reaction forward. Furthermore, the presence of heterostructured materials, such as metal oxide or metal–carbon composites, can further enhance the charge separation and catalytic activity of laser-generated particles [338]. These intricate structures create a synergistic effect between the different components, where the unique properties of each material, such as the high conductivity of the metal and the redox capabilities of the oxide, can work together to optimize the charge dynamics at the particle–electrolyte interface. This synergistic coupling can lead to improved charge separation, effective utilization of photogenerated charge carriers, and enhanced overall catalytic performance towards the OER [339].

Another critical mechanism underlying the enhanced catalytic performance of laser-generated particles in the OER is the improved adsorption and activation of water molecules on the particle surface. Laser-synthesized particles often possess a high concentration of structural defects, such as oxygen vacancies or metal-rich sites, which can serve as active centers for the adsorption and activation of water [222,280,340–342]. These defects create localized electronic states within the bandgap of the material, facilitating the transfer of electrons and protons during the initial water oxidation step, a crucial rate-limiting process in the multistep OER [285]. The presence of these structural defects alters the electronic structure of the material, allowing for more efficient charge separation and transport. This, in turn, enhances the ability of the catalyst to facilitate the water oxidation reaction, a key step in the overall oxygen evolution process [285,343–345]. The improved adsorption and activation of water molecules on the defect-rich surface of the laser-generated particles is a critical factor contributing to their enhanced catalytic performance in the OER [85,231,240,256,346–347]. By lowering the energy barrier for this initial step, the presence of these defects can significantly enhance the kinetics of the overall OER, leading to improved catalytic activity [288,348]. Furthermore, the unique morphological features of laser-generated particles, such as high surface area and controlled exposed facets, can provide a large number of accessible active sites for the adsorption, activation, and subsequent oxidation of water molecules [349]. This synergistic effect between the structural defects and the tailored particle morphology contributes to the overall enhancement in the catalytic performance of these laser-synthesized materials towards the OER.

Despite the significant progress in the development of laser-generated particles for the OER, there remain several challenges and future perspectives that need to be addressed to further improve the performance and practical applications of these materials. While laser-based synthesis offers precise control over particle properties, scaling up production for industrial applications remains a significant challenge. Literature predominantly focuses on lab-scale synthesis and characterization, with limited information on practical large-scale manufacturing techniques. For instance, a study reported the synthesis of ruthenium NPs with grain boundaries via LAL, which enhanced their OER activity [285]. However, the study did not address the scalability and feasibility of this method for mass production. Similarly, in the ultrafast fabrication of copper oxide micro/nanostructures, the focus remains on the synthesis process and electrochemical characterization, rather than investigating the scalability and cost-effectiveness of the approach [288]. This lack of emphasis on scalable production and cost analysis hinders the successful transition of these laser-generated catalysts from laboratory research to real-world industrial implementation [350]. Detailed techno-economic assessments comparing the laser-based synthesis approach with other established catalyst production methods, such as chemical precipitation or hydrothermal synthesis, are needed to evaluate the overall viability and competitiveness of these laser-engineered materials for large-scale OER applications [274]. Factors such as the capital investment required for laser equipment, operational expenses, material and energy consumption, and production yield must be thoroughly analyzed to determine the economic feasibility of scaling up the laser-based synthesis of highly active OER catalysts [237]. Only by addressing these critical scalability and cost-related challenges can the transformative potential of laser-generated particles be fully realized in the industrial deployment of advanced OER technologies.

Long-term stability and durability under real-world OER operating conditions are critical factors for the practical application of electrocatalysts. While some studies mention the stability of laser-generated particles, detailed investigations under realistic, extended-duration OER testing are often lacking. For instance, Li et al. [288], reported “excellent durability” for the fabricated copper oxide catalysts, but the study does not provide specific data on their long-term performance, such as chronoamperometry or chronopotentiometry measurements to quantify the stability over prolonged operation. Similarly, the high OER activity of laser-processed Ni–Fe alloys, achieving 100 mA·cm^−2^ at 464 mV overpotential was demonstrated [284]. However, the long-term stability and degradation behavior of these alloys under continuous OER operation were not thoroughly investigated. Quantifying the rate of performance degradation and understanding the underlying degradation mechanisms are crucial steps in developing strategies to enhance the durability and lifetime of these laser-generated OER catalysts, enabling their successful implementation in practical applications. While techniques like X-ray diffraction, X-ray photoelectron spectroscopy, and scanning electron microscopy provide valuable structural information, such as crystal structure, elemental composition, and morphology, as demonstrated in [284], correlating these static features with the dynamic OER activity requires more in-depth analysis. For example, density functional theory calculations, as utilized in [351], can provide valuable insights into the role of specific structural features, like single-atom Co sites, in catalytic processes like H_2_O_2_ photosynthesis. Similar computational studies are needed to elucidate the active sites and reaction mechanisms governing the enhanced OER performance of laser-generated catalysts. Furthermore, advanced experimental characterization techniques, such as in situ X-ray absorption spectroscopy or operando Raman spectroscopy, could offer valuable insights into the dynamic changes occurring at the catalyst–electrolyte interface during the OER process, shedding light on the structure–activity relationships of these intricate laser-synthesized materials.

Integrating laser-generated NPs into practical devices for the OER requires careful consideration of electrode fabrication and design. Achieving a uniform dispersion of the NPs on conductive supports, maintaining robust electrical contact between the catalyst and the support, and ensuring efficient mass transport of reactants and products to the active sites are all crucial factors for optimizing the overall catalytic performance. While a previous study [352] has discussed the integration of a catalytic module into a specific reactor design, broader research is needed on developing comprehensive integration strategies for various electrode architectures. This integration research should focus on several key aspects. First, the loading and distribution of the laser-generated NPs on the conductive support must be optimized to maximize the number of accessible active sites while minimizing mass transport limitations. The choice of conductive support material, such as carbon-based supports or conductive oxides, can also significantly impact the electrical conductivity, adhesion, and stability of the electrode [353]. Additionally, the overall electrode design, including factors like porosity, thickness, and three-dimensional (3D) structure, should be carefully engineered to balance efficient mass transport, electrical connectivity, and total catalytic activity. By addressing these integration challenges, the full potential of laser-generated NPs can be realized in practical OER devices, leading to enhanced performance and minimized losses.

#### 3.7 Surface-enhanced Raman spectroscopy

SERS is a highly sensitive and powerful analytical technique for molecular analysis [354–356]. It appears to be a potential future sensing technique because of its high sensitivity (down to the single molecule level), speed, and ability to offer bond-specific and structural information about analytes [357–358]. SERS is superior to other sensing technologies [359–361] not only in the sensitive and specific identification of analytes. It also extends its applicability by revealing numerous characteristics of molecules when combined with other methods such as chromatography, calorimetric techniques, and microfluidic devices [362–366]. For the same reason, experts in chemistry, physics, biology, medicine, archaeology, forensic sciences, and the military significantly depend on it for tasks like monitoring food quality, detecting micro-organisms, diagnosing cancer, detecting pollutants, and explosives [367–374].

In 1974, a roughened silver electrode exhibited anomalous Raman intensity for adsorbed pyridine molecules during an electrochemical experiment, which led to the accidental discovery of the surface-enhanced Raman scattering effect [375]. Later this effect was studied in detail by Jeanmarie and Van Duyne, analyzing more molecules and observing disproportionate signal strength with respect to concentration, which was later attributed to the electromagnetic enhancement mechanism [376].

The SERS effect includes chemical enhancement and electromagnetic enhancement. Currently many researchers consider electromagnetic enhancement as the major mechanism that contributes to the observed signal enhancement. The excitation of localized surface plasmons in noble metals like Ag or Au generates a resonance effect known as LSPR. This enhancement is called electromagnetic enhancement [377]. The electromagnetic enhancement primarily depends on the morphology and structural features of plasmonic structures. Increased charge density at sharp vertices causes lightning rod effects. Thereby, it strengthens the local electric field and causes spatially discrete areas called “hot spots”. Molecules of interest located in these hot spots experience an increased Raman cross section. This effect is independent of the analyte molecule, and the enhancement factor can be as high as 10^14^. This can enable the detection of single molecules [356,377]. In contrast, chemical enhancement is analyte-specific and is based on charge transfer mechanisms between analyte molecules and the substrate surface [356]. Chemical enhancement can achieve enhancement factors of the order of 10^4^.

The synthesis, manufacturing and detection strategy of SERS sensors significantly impacts their final performance. The most widely used synthesis strategies for plasmonic nanomaterials are conventional techniques such as seed-mediated growth and citrate reduction of gold and silver salts (HAuCl_4_ and AgNO_3_) [366–367]. In order to get the appropriate plasmonic characteristics, surfactants, capping agents and other limiting reagents are also used. These techniques provide benefits in controlling the size and aspect ratio of NPs, but they limit the nanoparticle purity.

One of the most effective techniques for creating plasmonic NPs, particularly for SERS applications, is LAL [7,378–379]. Unfavorable interference in SERS spectra caused by precursors and other chemicals employed as surfactants during synthesis is a problem that SERS practitioners worldwide constantly deal with. Nanoparticles produced using these synthetic techniques are frequently purified using a number of purification processes [380]. This is where liquid laser ablation shines because it can produce ultrapure nanocolloids offering flexibility in the dispersion medium. Several efforts have been made to produce reliable and high-performance SERS substrates.

Interparticle hot spots are the major hot spots formed in Au and Ag nanosphere assemblies with spatially narrow regions. It is possible to tune the intensity of electric field between nanosphere dimers as a function of interparticle distance. Theoretical calculations show that with increasing distance between NPs (*a*), the electromagnetic field *E* decreases by |*E*|^4^ ~ 1/*a*^4^ [381]. However, as the gap size becomes less than 2 nm, an electric current begins to start flow between the NPs, adversely affecting field enhancement. Non-uniformity in the size of NPs is another disadvantage of LAL while creating plasmonic NPs. Capping agents and surfactants are used to overcome this limitation, but they may have a negative impact on SERS performance. To create nanogap-tailored SERS sensors, it is important to have control over size and arrangement of NPs.

**Figure 27:**
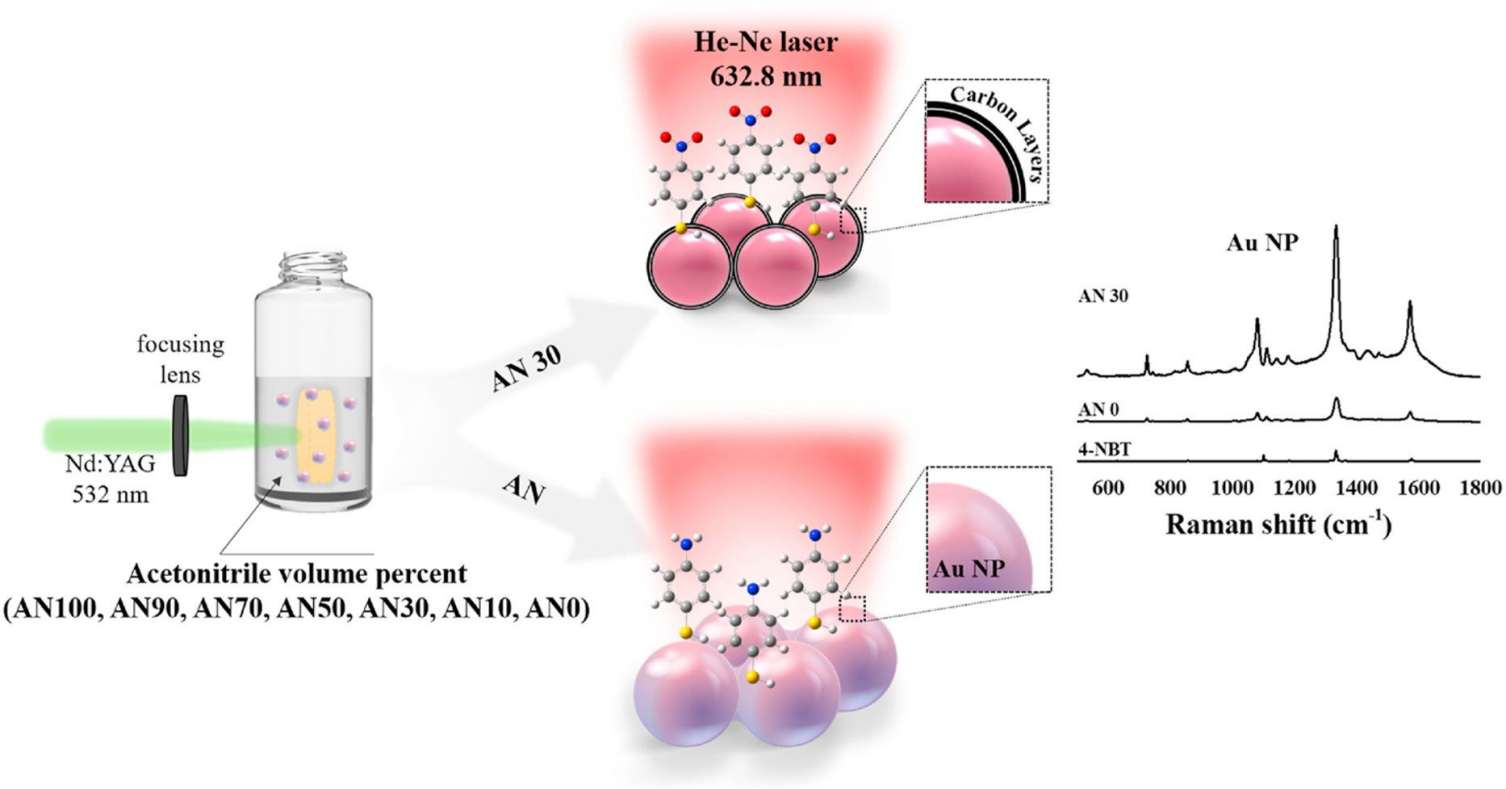
Illustration showing synthesis and SERS performance of Au nanoparticles with varying graphitic carbon coating thickness. Figure 27 was reprinted from [383], *Biosensors and Bioelectronics*, vol.197, by S. J. Lee; H. Lee; T. Begildayeva; Y. Yu; J. Theerthagiri; Y. Kim; Y. W. Lee; S. W. Han; M. Y. Choi, “Nanogap-tailored Au nanoparticles fabricated by pulsed laser ablation for surface-enhanced Raman scattering”, article no. 113766, Copyright (2022), with permission from Elsevier. This content is not subject to CC BY 4.0.

Laser ablation of Au in a mixed solvent of water and acetonitrile to form a graphite layer on the surface of NPs is an approach for creating ordered surface morphology [382]. In the work, the authors found that by varying the ratio of solvent mixtures, the number of carbon layers can be controlled. The nanogaps between the NPs are controlled by varying thickness of graphitic carbon layer. The carbon layer acted as an inhibitor in the growth of NPs allowing size control. The SERS sensors are made by drop casting and drying the nanocolloid on a Si wafer as shown in Figure 27.

Au NPs ablated in a mixture containing 30 vol % of acetonitrile in water yielded strong SERS spectra of both 4-nitrobenzenethiol and 4-aminobenzenethiol with an enhancement factor of the Raman signals attributed to 4-nitrobenzenethiol and 4-aminobenzenethiol reached about 10^13^. They also investigated the catalytic properties of these nanogap-tailored NPs with graphitic carbon layers and found they performed better than Au NPs [383].

**Figure 28:**
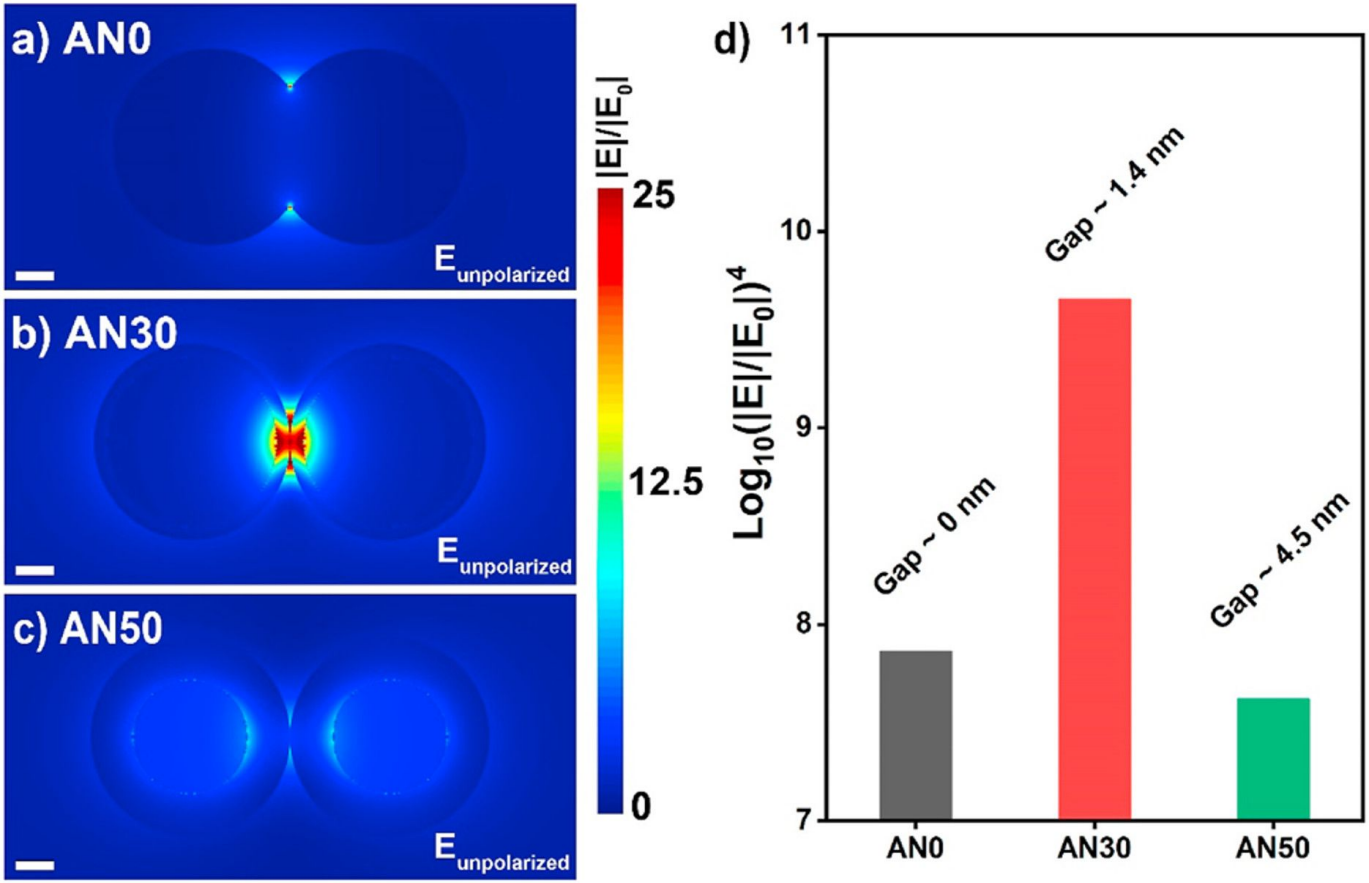
FDTD-simulated electric field amplitude distributions under unpolarized lights of (a) AN0, (b) AN30 and (c) AN50 with λ indicate 2 nm. (d) Calculated SERS signal intensity of the corresponding nanogap-tailored Au NPs. Figure 28 was reprinted from [383], *Biosensors and Bioelectronics*, vol.197, by S. J. Lee; H. Lee; T. Begildayeva; Y. Yu; J. Theerthagiri; Y. Kim; Y. W. Lee; S. W. Han; M. Y. Choi, “Nanogap-tailored Au nanoparticles fabricated by pulsed laser ablation for surface-enhanced Raman scattering”, article no. 113766, Copyright (2022), with permission from Elsevier. This content is not subject to CC BY 4.0

Finite-difference time-domain (FDTD) simulations were also utilized for visualizing interparticle hot spots formed between the nanospheres, supporting effective concentration of electric field by AN30 NPs as shown in Figure 28. The authors concluded that the best SERS enhancement occurred when the size of the NPs was optimum for maximum adsorption of analyte molecules on their surface and the nanogaps among them are narrow enough (1.4 nm) to form intense hot spots.

**Figure 29:**
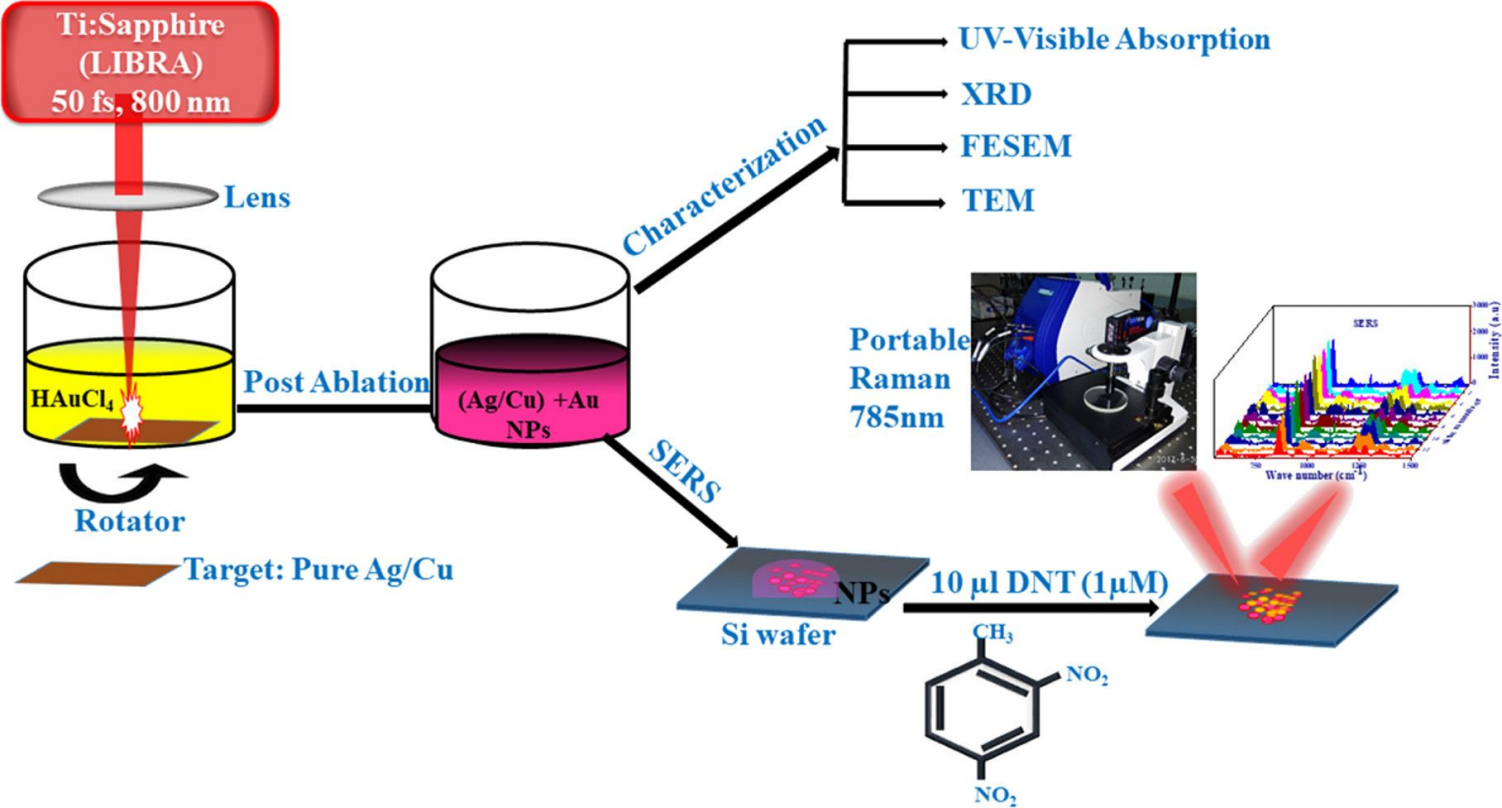
Schematic representation of core–shell nanoparticle production using laser ablation and their SERS performance. Figure 29 was reproduced from [386], (©2018 M. S. S. Bharati et al., published by Frontiers in Physics, distributed under the terms of the Creative Commons Attribution 4.0 International License, https://creativecommons.org/licenses/by/4.0).

Compared to monometallic NPs, bimetallic NPs show improved SERS properties due to the synergic effect between individual monometallic components [384–385]. There are different types of bimetallic NPs, such as core–shell, alloyed, multishell, and multiwall NPs, with tunable plasmonic properties according to their composition, structure, and aspect ratio [356]. Even though chemical reduction is the most widely used technique for bimetallic NPs for SERS applications, laser ablation can also be effectively employed to create alloyed and core–shell NPs (Figure 29). For example, Ag@Au and Cu@Au core–shell NPs were created by ablation of Ag and Cu targets in HAuCl_4_ to get bimetallic NPs with Ag and Cu cores surrounding Au shell [386].

**Figure 30:**
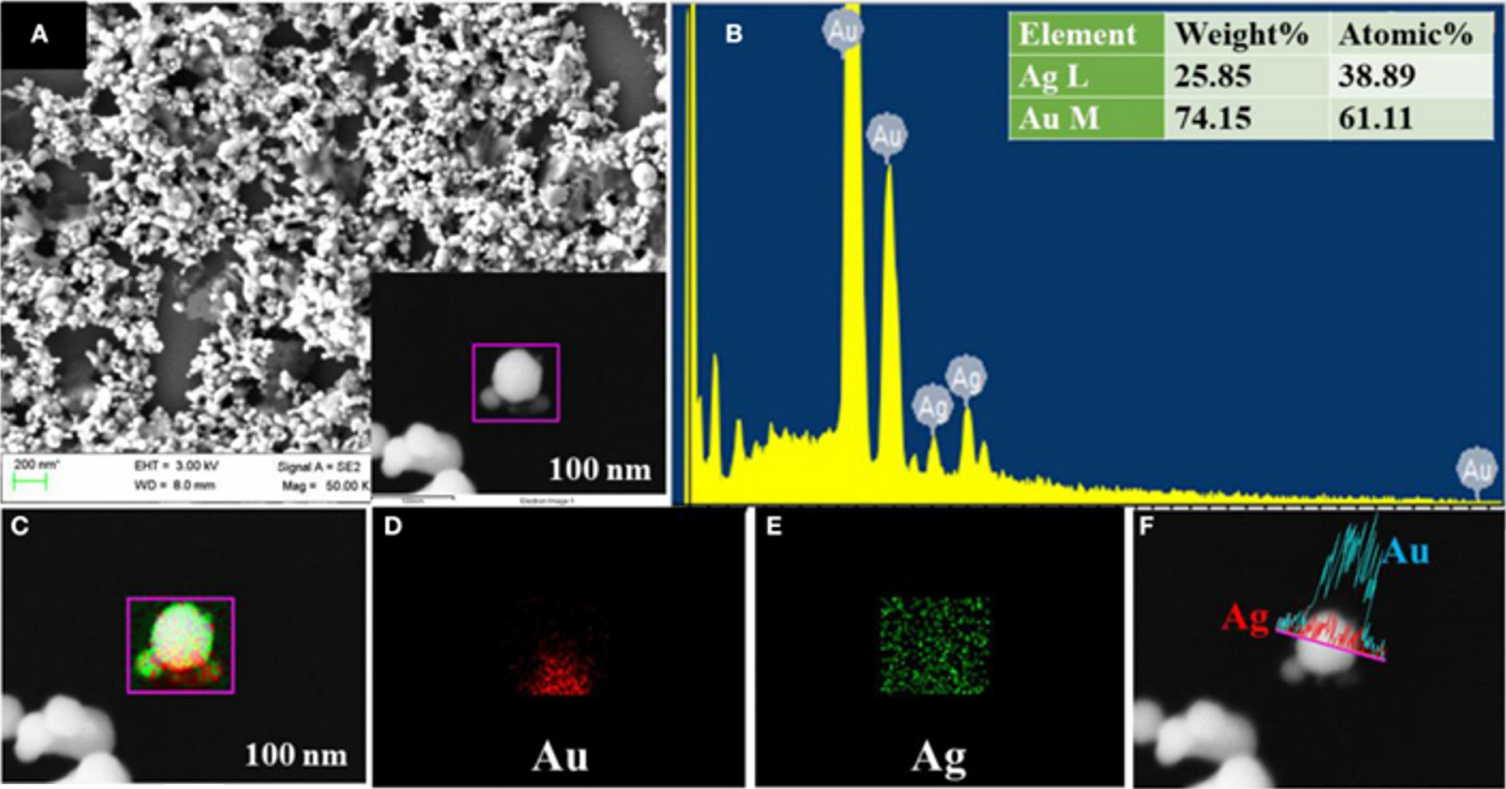
(A) FESEM image of Ag@Au NPs with the inset illustrating the single NP image in the 100 nm scale. (B) EDS spectra with inset depicting the wt % of individual elements. (C) EDS mapping image of the Ag@Au NPs. (D) Au EDS map. (E) Ag EDS map. (F) Line profile of the Ag@Au particle. Figure 30 was reproduced from [386] (©2018 M. S. S. Bharati et al., published by *Frontiers in Physics*, distributed under the terms of the Creative Commons Attribution 4.0 International License, https://creativecommons.org/licenses/by/4.0).

The SPR peak of Ag@Au NPs synthesized by laser ablation was observed at 566 nm with a redshift and broadening compared to pure Ag (≈400 nm) and pure Au (≈520 nm) NPs. The broad absorbance indicates the presence of NPs with a wide size distribution and non-spherical surface morphology, as well as a possible formation of nanoaggregates. FESEM images (Figure 30) of the laser-generated NPs showed bulged, elongated, and quasispherical NPs. The fragmentation of previously formed NPs in the colloid due to reirradiation may have resulted in variations in their shape. The authors concluded that the discrepancy may be due to the influence from the concentration of precursors, irradiation energy, and time of ablation.

Because AuCl_4_^−^ species have a greater reduction potential, femtosecond laser pulses interact with the precursor HAuCl_4_ to generate Au ions. The ablated Ag and Au ions may have undergone a replacement process. Rapid nucleation and growth development results in the creation of bimetallic or alloy NPs. Bimetallic NPs were directly drop-cast and dried on a Si wafer to fabricate SERS sensors. Also, the signal for the C=C stretching vibration of methylene blue at 1622 cm^−1^ had a relative standard deviation of 7.45, which is excellent for AuAg bimetallic NPs. The produced SERS substrates showed detection limits of 10^−6^, 10^−6^, and 10^−9^ M for 2,4,6-trinitrophenol, 2,4-dinitrotoluene, and methylene blue with high sensitivity, reproducibility, and chemical stability [386].

**Figure 31:**
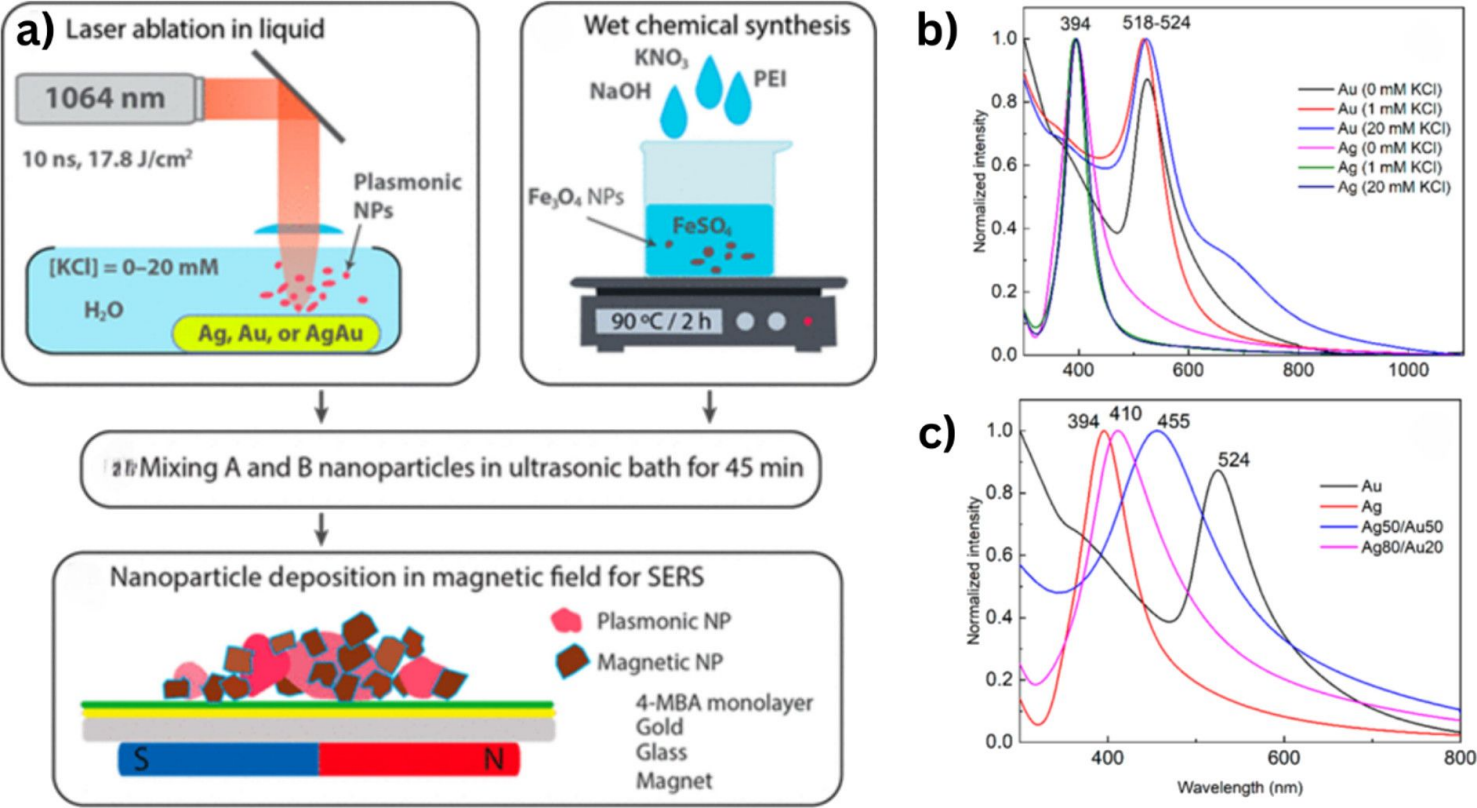
(a) Magnetic nanoparticles (Fe_3_O_4_) are synthesized by a wet chemical method and after an ultrasound treatment, the decorated nanoparticles of Fe_3_O_4_@Ag, Fe_3_O_4_@Au, Fe_3_O_4_@Ag_80_Au_20_, and Fe_3_O_4_@Ag_50_Au_50_) were collected by using a strong magnet. UV−vis spectra of nanoparticles obtained by PLAL of (b) Au and Ag targets in 0−20 mM KCl solutions and (c) Ag, Au, Ag_80_Au_20_ and Ag_50_ Au_50_ targets in pure water. Figure 31a–c was reproduced from [385], (©2023 M. Talaikis et al., published by ACS, distributed under the terms of the Creative Commons Attribution 4.0 International License, https://creativecommons.org/licenses/by/4.0).

Another approach for creating SERS sensors is by combining plasmonic and magnetic NPs by LAL [385]. Au, Ag, and bimetallic nanocolloids were prepared by laser ablation of metallic and bimetallic targets of different compositions (Figure 31a). The authors also took the advantage of using ionic salt solutions as ablation medium since this increases the stability of nanocolloids and prevents agglomeration by maintaining electrical balance. In this work, 50:50 Au–Ag bimetallic NPs created by laser ablation showed an absorption maximum at 455 nm, almost halfway between that of pure Au (524 nm) and pure Ag (394 nm) nanocolloids. A shift in SPR was evident in this study as shown in Figure 31b,c.

The collection efficiency and thereby SERS enhancement can be improved by using magnetic NPs as the narrow nanogaps formed due to aggregation can act as a source of intense hotspot. Fe_3_O_4_@Ag nanoparticle SERS sensitivity was assessed by probing the physiologically significant biomolecule adenine. The authors found that the detection limit was 10^−7^ M and that the sensor can be used for quantitative detection of adenine in the 10^−3^ to 10^−7^ M range [385]. Also tuning of plasmonic properties of bimetallic NPs can be done by varying concentration, but the authors discovered a drop in SERS intensity when it is coupled with magnetic Fe_3_O_4_ NPs.

Other types of SERS sensors are based on surface modification of templates such as metal sheets, Si, anodic aluminum oxide, PVC films, and glass [382,387–388]. These synthetic approaches excel in creating ordered and periodic nano/microscale plasmonic structures. They include lithography-based SERS sensor fabrication methods, such as electron lithography, ion beam etching, photolithography, and nanosphere lithography [389–391]. These approaches excel in the creation of controlled and ordered nanostructures on the substrate surface. However, these methods are not commonly used because they are expensive and time-consuming. Laser nanostructuring and direct laser writing gained importance since they can be utilized to create nanostructured surface morphologies in large areas with precise control [392–393]. These methods are less expensive with very high fabrication efficiency, more accessible, and faster than lithography-based techniques with comparable SERS performance [394–395].

In another work, an amplified femtosecond laser was employed in a laser micromachining system for laser direct writing on a glass chip with microchannels and on a SERS-active substrate [396]. The cleaned silicon wafer substrates were submerged in diluted aqueous solutions of silver nitrate. During the line-by-line machining operation, the samples were translated by the motion stage. The samples were washed in deionized water to remove any remaining silver nitrate solution following the machining operation. By comparing the SEM images of laser direct writing in deionized water and silver nitrate solution, the effects on surface morphology can be studied. Grating like periodic nanostructures on glass with submicrometer period were distributed uniformly all over the area after laser writing in water [396]. In contrast, after the sample was laser processed in aqueous solutions of silver nitrate, granular nodules attached to the machined surface were observed. The particle sizes ranged from tens to hundreds of nanometers [396]. Multiphoton reduction yielded the silver NPs from silver nitrate. These SERS substrates were tested for their performance with rhodamine 6G from 10^−7^ to 10^−10^ M, clearly showing multiple enhanced vibrational bands of R6G. Also, the normalized SERS intensity at 614 cm^−1^ showed a linear relation with the concentration of R6G [397]. Even though 10^−10^ M was the lowest concentration experimentally detected, with a 1 s accumulation period and 1 mW laser excitation power, the detection limit, as determined by linearly fitting the data, was around 10^−17^ M. For this, the authors extrapolated the data until the SERS intensity drops to zero. The estimated detection limit is 10^−15^ M for a signal with an intensity of 100 counts due to possible noise at this detection limit. Dynamic measurement and sample protection were made possible by the quick accumulation time and low laser excitation power.

**Figure 32:**
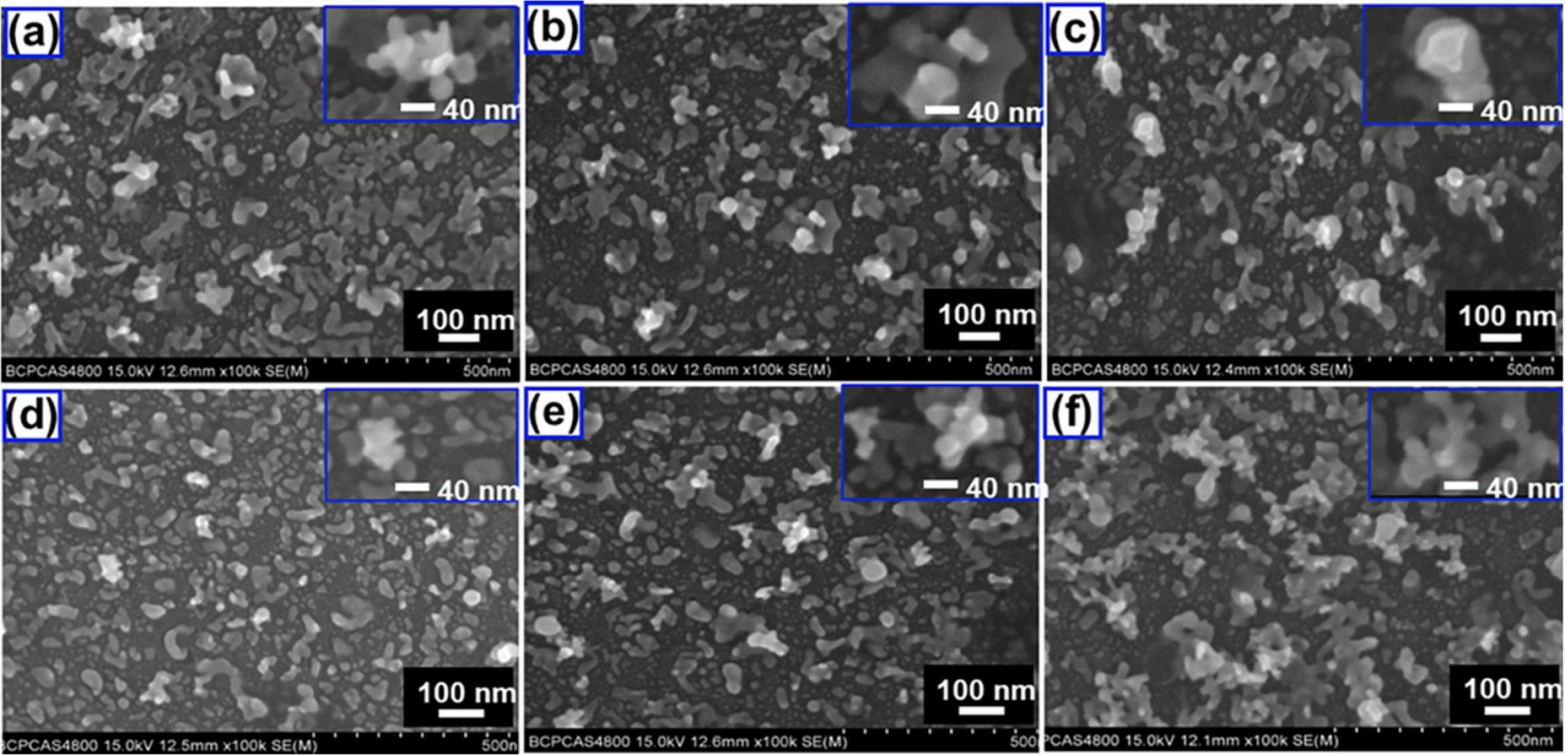
SEM images of AuNP matrix under different concentrations of colloidal solution and laser fluences. SEM images of the AuNP matrix using a concentration of 70 mg/mL of the colloidal solution and laser fluences of 4.4 mJ/cm^2^ images of the AuNP matrix at a laser fluence of 5.6 mJ/cm^2^ (a), 5.6 mJ/cm^2^ (b), and 6.8 mJ/cm^2^ (c). SEM using concentrations of the colloidal solution of 50 mg/mL (d), 70 mg/mL (e), and 100 mg/mL (f). Insets are the enlarged SEM images of the 3D AuNP stacks in panels (a)−(f). Figure 32 was reproduced from [398] (©2024 C. Huang et al., published by ACS, distributed under the terms of the Creative Commons Attribution-NonCommercial-NoDerivatives 4.0 International License, https://creativecommons.org/licenses/by-nc-nd/4.0/). This content is not subject to CC BY 4.0.

Femtosecond laser direct writing (FLDW) technology is another technique effectively used for the fabrication of uniform, high-density hot spots on templates such as glass, ITO, and silicon [398]. It offers important advantages, such as 3D processing capability and maskless fabrication as an alternative to expensive focused beam techniques. Compared to traditional 2D SERS structures, a 3D SERS active platform can extend the interaction area and ensure the ability to directly detect trace amounts of molecules. FLDW was applied directly onto ITO substrates after colloidal AuNPs had been spin-coated onto them [398]. A random AuNP matrix was produced by the transient thermal effect produced when femtosecond laser pulses interacted with colloidal AuNPs. This structure is made up of aggregates of Au NPs with sharp edges and irregular shapes that are very dense with hot spots. Thus, the substrate’s SERS performance is greatly improved by the large number of 3D AuNP stacks as shown in Figure 32.

**Figure 33:**
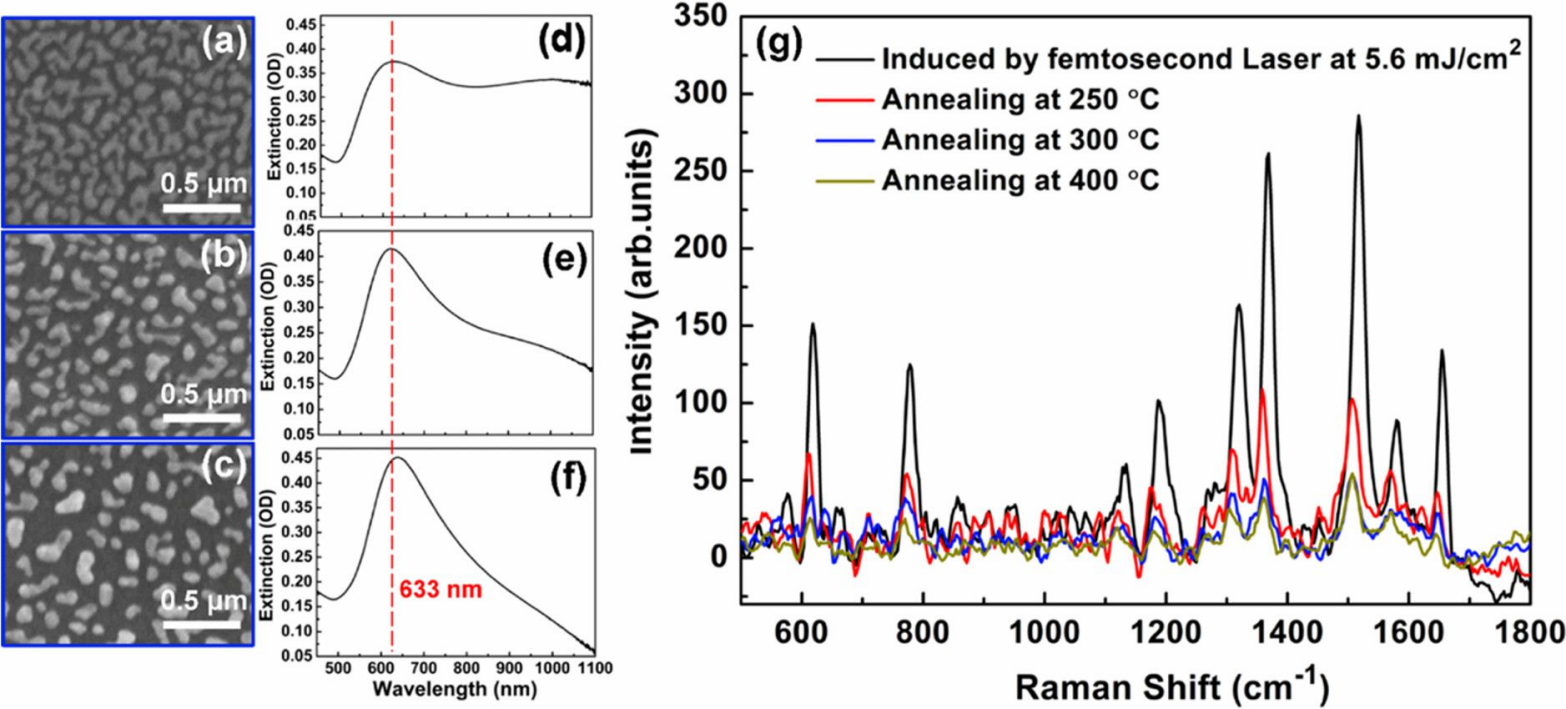
(a−f) SEM images (left panel) and optical extinction spectra (right panel) of samples annealed at temperatures of 250 °C (a, d), 300 °C (b, e), and 400 °C (c, f). (g) SERS spectra with 633 nm excitation using the FLDW technique at a laser fluence of 5.6 mJ/cm^2^ for a 10^−8^ mol/L R6G solution (black curve) and SERS spectra from samples annealed at temperatures of 250 °C (red curve), 300 °C (blue curve), and 400 °C (brown curve) for a 10^−6^ mol/L R6G solution. Figure 33 was reproduced from [398] (©2024 C. Huang et al., published by ACS, distributed under the terms of the Creative Commons Attribution-NonCommercial-NoDerivatives 4.0 International License, https://creativecommons.org/licenses/by-nc-nd/4.0/). This content is not subject to CC BY 4.0.

Annealing in a muffle furnace at three temperatures (250, 300, and 400 °C) produced Au nanoisland structures with varying width gaps and diameters. The SEM images in Figure 33a–c demonstrate that when the annealing temperature rises, so do the diameters and gap widths of the Au nanoislands. At an annealing temperature of 250 °C, a few linked Au nanoislands are produced (Figure 33a).

The interconnected Au nanoislands split into isolated ones as the temperature rises (Figure 33b,c), displaying smooth, regular forms like ellipses and circles. When R6G molecules are used as a probe, these sensors showed exceptional sensitivity, producing good Raman signals even at concentrations as low as 10^−8^ M. Compared to conventional annealing techniques, this sensitivity is three orders of magnitude greater. For Raman vibrations at 1368 cm^−1^, the RSD of the Raman intensity, computed using more than 50 spectra, was 5.1%, ensuring good signal homogeneity.

Nanoparticles created by LAL are often utilized along with other methods to create different 3D surface morphologies because plasmonic NPs obtained from other techniques are often contaminated, either lowering their SERS activity or influencing their applications. The greatest advantages of this production process are low cost, simple management, and the absence of chemical reagents. For example, EPD in a Au colloidal solution produced by laser ablation in water is a straightforward and environmentally friendly method of creating 3D structured Au porous films [131]. Initial laser ablation for 20 min in water results in the formation of spherical Au NPs varying from 20–80 nm. The FCC structure of gold was also identified from the diffraction pattern [131]. The pre-formed large NPs were broken into smaller ones during subsequent laser ablation. Through collision-induced aggregation and laser-induced sintering, the NPs in the solution formed chain-like structures when the colloidal concentration was high enough during laser ablation. Then the Au nanochains were deposited on the substrate surface via EPD in the Au colloidal solution. The resulting film was composed of Au nanochains and was rough and porous at the nanoscale but uniform at the macroscale. Because of its special rough structure which contributed to hot spots, it also performed very well in SERS. Additionally, this method was used to create more 3D netlike films. The best SERS sensors with a 3D porous film of nanochains made by EPD after 60 min of laser ablation showed a minimum detectable concentration of R6G of 10^−13^ M when using a short data integration time of 5 s. The sensors made by EPD of the nanocolloid after 20 min of laser ablation developed could also amplify the Raman bands of R6G at 1 nM concentration. The dimeric coupling and interparticle hot spots formed in the sub-10 nm gaps may have caused the observed signal amplification. The SERS performance was obviously better than that of pure Au film due to the presence of nanofeatures on the substrates. The C=C stretching modes of R6G were clearly identified even at 10^−13^ M concentration.

**Figure 34:**
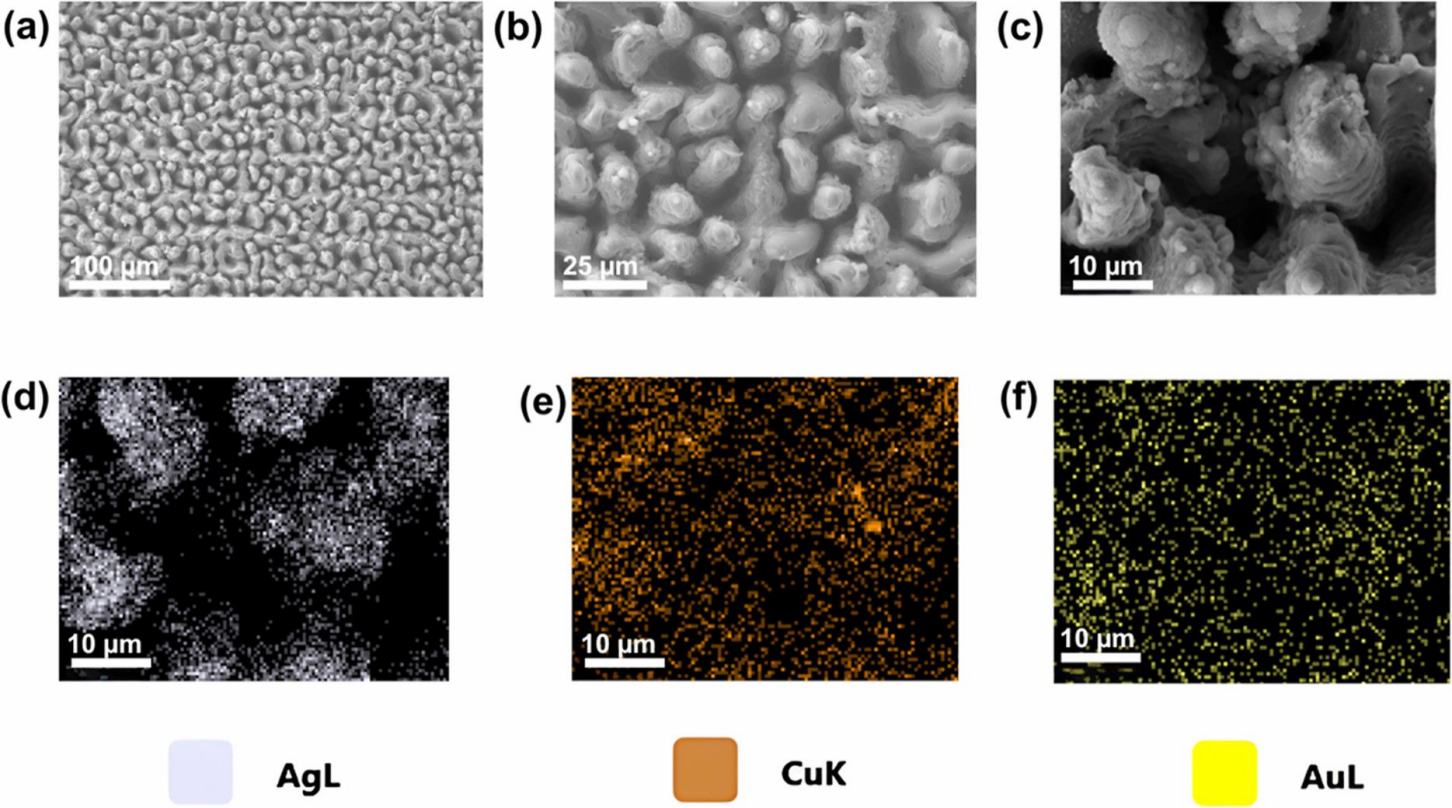
(a–c) SEM images showing CuAg alloy micropillars incorporated with AuAg bimetallic nanoparticles, EDS mapping of (d) Ag, (e) Cu and (f) Au. Figure 34 was adapted with permission from [399], Copyright 2025 American Chemical Society. This content is not subject to CC BY 4.0.

Laser-assisted surface modification (laser patterning) in liquids is another technique used for creating nano/microscale SERS-active structures. Kundalam Kadavath et al. demonstrated an innovative approach of laser scribing in a nanocolloid for simultaneous surface restructuring and in situ incorporation of bimetallic nanocolloids on micropillar morphology [399]. Cu and Ag thin films deposited on a Si wafer were used as substrates for the SERS sensors. Laser ablation of a AuAg bimetallic target resulted in AuAg bimetallic NPs (diameter 13.4 ± 2.7 nm) with an absorption maximum at 449 nm. Laser scribing in bimetallic nanocolloids resulted in the following (1) alloying of Cu and Ag thin films, (2) surface-restructuring to micropillar morphology, and (3) in situ incorporation of AuAg bimetallic NPs as shown in Figure 34.

**Figure 35:**
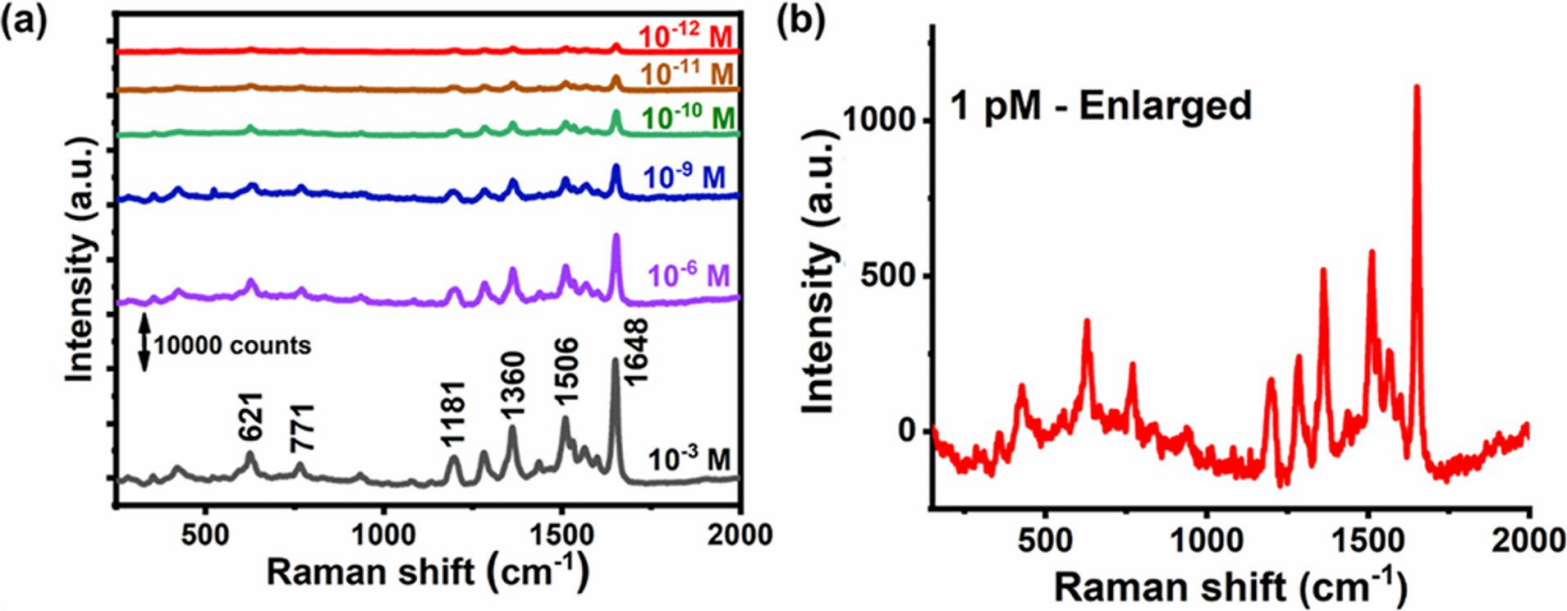
SERS performance of micropillar sensors created by laser patterning in nanocolloid using R6G. Figure 35 was adapted with permission from [399], Copyright 2025 American Chemical Society. This content is not subject to CC BY 4.0.

Alloyed 3D surface structures coated with bimetallic NPs can be created easily and efficiently using laser patterning in liquid. These structures are particularly important in the case of SERS sensors as the synergistic effect between Cu, Ag and Au can be utilized along with additional hot spots and nanogaps contributed by surface distribution of NPs. Furthermore, the edges/corners generated on the micropillars due to laser-induced alloying and solidification of Ag and Cu thin films contribute to electromagnetic enhancement. These factors contributed to the ultrasensitive detection (1 × 10^−12^ M) of R6G with well distinguished Raman bands as shown in Figure 35.

Laser-induced alloying and surface modification, together with in situ integration of bimetallic NPs, is a novel approach that promises considerable advancement in SERS sensor production. It distinguishes itself from other traditional approaches by combining great sensitivity with the potential for large scalability. It is also incredibly quick and repeatable, as well as capable of producing long-range homogenous plasmonic structures. These sensors might also overcome SERS signal non-uniformity caused by uneven configurations of SERS active structures. Other frequent issues, such as stability and reusability, are also addressed. These SERS substrates are stable and may be reused following a simple microwave treatment to remove analytes from the surface.

Additionally, by varying the percentage of component metals like Au, Ag, and Cu in bimetallic NPs, the sensor’s plasmonic resonance may be adjusted for optimal SERS performance. Multimetallic NPs of Au, Ag, Cu, Pd, and Pt with diverse structures such as multishell, multiwall, alloyed, and Janus structures can be created by laser processing in metal salts, reactive ablation, fragmentation, and controlled alloying of metals. Laser-assisted reduction and nucleation of NPs with the presence of limiting reagents can be utilized for synthesis of plasmonic NPs of diverse shapes. Effects of electric and magnetic fields on the morphology of NPs during laser ablation can be studied for the creation of diverse plasmonic NPs. Laser-assisted synthesis can be scaled easily, and portable and flexible SERS sensors can be developed for onsite detection of molecules. These sensing platforms can be even combined with machine learning algorithms and portable Raman spectrometers allowing for a practical and reliable applicability of SERS. This implies that even when exposed to several excitation wavelengths, stable SERS sensors created by laser scribing in nanocolloids offer a great deal of promise for the ultrasensitive detection of different analyte molecules. Table 5 summarizes key areas of research in laser-based nanomaterial synthesis, categorizing representative studies and highlighting their scientific contributions. It covers topics from ligand-free nanoparticle generation to prototype device fabrication, emphasizing their significance in optoelectronics, catalysis, energy, and sensing technologies.

**Table 5:** Scientific contribution and technological significance of laser-generated nanocolloids in thin film fabrication and device applications.

Research focus	Representative studies (as cited)	Scientific contribution and technological significance

ligand-free nanoparticle synthesis (LAL)	[13–14,31]	enables the creation of ultrapure, surfactant-free colloidal solutions, facilitating direct integration into optoelectronic devices without the need for surface passivation or post-synthesis cleaning procedures
monodisperse and ultrasmall nanoparticle production (LFL)	[31–34]	permits precise control over optical properties through the generation of narrowly distributed, sub-5 nm NPs, which are critical for applications in plasmonics, ultraviolet photodetection, and catalysis
submicrometer sphere formation (PLML)	[41,52–53]	facilitates the scalable production of mechanically robust, spherical particles in the submicrometer range, leading to improved particle packing density and film uniformity, with benefits for photovoltaic devices and sensor technologies
tailored defect engineering (LDL)	[85–91]	allows for the strategic incorporation of vacancies and dopants to modulate charge transport characteristics, surface reactivity, and electrochemical performance in applications such as hydrogen evolution/oxygen evolution reactions and sensing
scalable thin film assembly (EPD, spray deposition)	[127,129,134]	enables the fabrication of high-quality, large-area thin films using nanocolloids on a variety of substrates, which is crucial for translating nanomaterials into practical solar cells, photodetectors, and sensor devices
prototype device realization (PVD, PD, SERS)	[109,130,134]	validates the application potential of laser-synthesized colloids through the creation of functional devices, including heterojunction solar cells, surface-enhanced Raman scattering-active substrates, and nanostructured photodetectors

#### 3.8 Magnetic nanoparticles

Laser processing of nanomaterials in solution by ablation/irradiation/fragmentation is able to produce magnetic nanoparticles (MNPs)/nanomaterials with unique physicochemical properties compared to other syntheses. Fabrication of magnetic nanostructures by LPL is particularly interesting because the magnetic properties are dependent on finite size effects, morphology, and surface effects. Changing size and morphology of MNPs can lead to single/multidomain particles with highly enhanced net magnetic properties. Among these MNPs, mostly magnetic oxide NPs, magneto-plasmonic alloy NPs, nanoparticle chains/strands under an external magnetic field, nanostructures of different MNPs/nanomaterials/nanocomposites, as well as intrinsically non-magnetic NPs showing ferromagnetism have been synthesized by LPL. The nanomaterials obtained by PLAL include Fe, Co, Ni, Gd, and Nd, their alloys as well as alloys with other elements (AuFe, NiCo, Nd_2_Fe_14_B, and SmCo), their oxides Fe_2_O_3_, Fe_3_O_4_, NiO, CoO, Gd_2_O_3_, Gd_3_Fe_5_O_12_, MnO, Mn_3_O_4_, Zn_1−_*_x_*Mn*_x_*O, CoFe_2_O_4_, and Cr_2_O_3_, or carbides such as Fe_3_C, Co_3_C among others. In MNPs of different types of magnetic materials, the magnetic properties could be a superposition of the properties of each individual magnetic material forming the resultant composition [400–401].

MNPs are useful in different areas/applications such as biomedicine (e.g., drug delivery, hyperthermia, magnetofection, magnetic resonance imaging, and magnetic separation), photonics/optoelectronics (e.g., Faraday rotation and magneto-optics), magnetic fluids (tunable viscosity), microwave frequency transducers, multiple state memory elements, highly transparent magneto-optic materials, and magnetic field sensors [361]. MNPs consist of atoms, ions, or molecules that have a net non-zero magnetic dipole moment mainly originating from unpaired electrons that can respond and resonate in a time-varying magnetic field. This can lead to transfer of energy from the exciting field to the NPs, which is used in radiotherapy and hypothermic processes [400].

Alloying and structure formation by LAL can lead to a new set of hybrid nanostructures having exciting magneto-optical properties. Magneto-plasmonic nanoparticles (MP NPs) represent a novel category of hybrid nanomaterials that combine the intrinsic properties of magnetic (such as Fe_3_O_4_ and Co) and plasmonic (such as Au and Ag) components to enhance their functionalities. A detailed review of fabrication, characterization, and magnetic properties of MNPs/composites of different morphologies, phases, and structures in solution processed by laser-assisted synthesis (laser ablation/irradiation) is available for further details [400].

Most of the studies focus on syntheses and properties of MNPs by LPL, which in general are metal oxide NPs. Also, the effects of different lasers and the liquid media are widely explored. Magnetic properties are studied for some of these MNPs. Thin films or device structures fabricated using laser-processed colloids of magnetic NPs are very rare in the literature. Iron oxide NPs are the most widely studied MNPs by LAL, while others are of Ni, Co, and SmCo. Fe_3_O_4_ and Fe_3_C NPs synthesized by laser ablation of Fe foil in ethanol using a 1064 nm Nd:YAG laser (750 ps) were superparamagnetic, and their magnetic viscosity was calculated from thermoremanent magnetization plots [397]. Phase-controlled synthesis of iron oxide NPs (α-Fe_2_O_3_ and γ-Fe_2_O_3_) was achieved by PLAL in ethanol, deionized water, and acetone, and their magnetic properties were analyzed using vibrational sample magnetometry (VSM) [402].

Laser ablation of an iron target in water and acetone using a femtosecond laser enabled the tuning of chemical and structural characteristics of iron and iron oxide NPs. The effects of morphology of NPs from liquid media were studied using TEM, Raman analysis, and X-ray photoelectron spectroscopy, and a formation mechanism was described [403].

**Figure 36:**
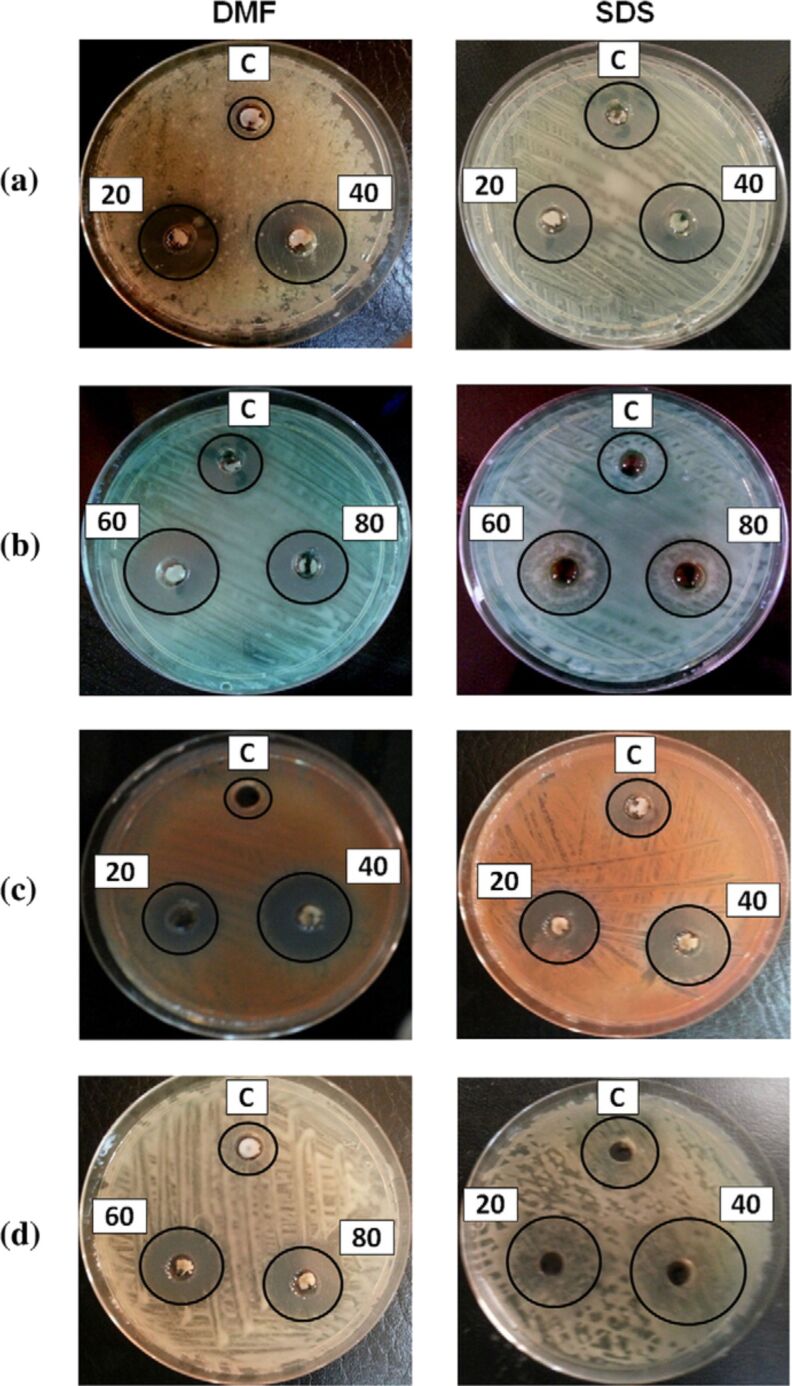
Zone of inhibition induced by iron oxide nanoparticles suspended in DMF and SDS solutions prepared at different laser fluencies against various microorganisms (a) *Escherichia coli*, (b) *Pseudomonas aeruginosa*, (c) *Serratia marcescens*, and (d) *Staphylococcus aureus*. Figure 36 was reprinted from [404], *Materials Science and Engineering: C*, vol. 53, by R. A. Ismail; G. M. Sulaiman; S. A. Abdulrahman; T. R. Marzoog, “Antibacterial activity of magnetic iron oxide nanoparticles synthesized by laser ablation in liquid”, pages 286-297, Copyright (2015), with permission from Elsevier. This content is not subject to CC BY 4.0.

Magnetic iron oxide (α-Fe_2_O_3_) NPs of 50–110 nm were synthesized at four different fluences by pulsed laser ablation (1064 nm, 9 ns) using an iron target in DMF and sodium dodecyl sulfate (SDS) solutions. The MNPs’ antibacterial properties were tested against gram-positive (*Staphylococcus aureus*) and gram-negative (*Escherichia coli*, *Pseudomonas aeruginosa* and *Serratia marcescens*) bacteria, observing a noticeable inhibition on bacterial strains (Figure 36). The antibacterial activity of these NPs was affected by the synthesis conditions and the synthesized magnetic NPs under the magnetic field effect were able to capture *S. aureus* bacteria [404].

SmCo NPs were obtained by PLAL of a SmCo target in phosphate-buffered saline, and their antimicrobial effects against *Staphylococcus aureus, Staphylococcus epidermidis, Enterococcus faecalis*, and *Streptococcus mutans* were determined using minimal inhibitory concentration assays [405].

**Figure 37:**
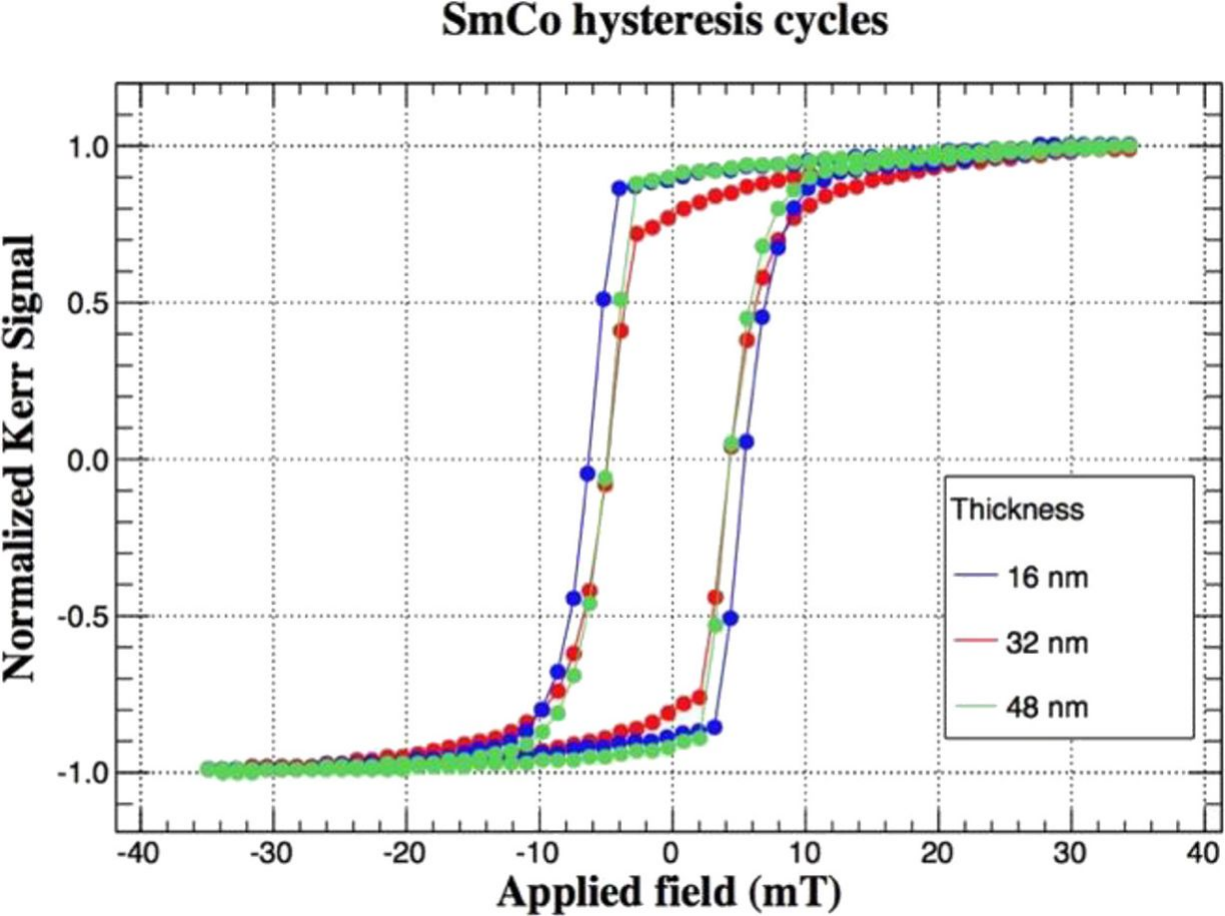
The graph shows hysteresis curve of NPs-SmCo, by magneto-optical Kerr effect measurements. Figure 37 was reprinted from [405], *Applied Surface Science Advances*, vol. 7, by M. V. Morone; F. Dell'Annunziata; R. Giugliano; A. Chianese; A. De Filippis; L. Rinaldi; U. Gambardella; G. Franci; M. Galdiero; A. Morone, “Pulsed laser ablation of magnetic nanoparticles as a novel antibacterial strategy against gram positive bacteria”, article no. 100213, copyright (2022) with permission from Elsevier. This content is not subject to CC BY 4.0.

The magnetic properties of SmCo NPs were determined using magneto-optical Kerr effect measurements after growing the NPs up on a thin amorphous film substrate. The spectra showed magnetic characteristics linked with particle dimensions. The magnetic coercive field is about 4.5 mT for NPs-SmCo material, as shown in Figure 37.

Pulsed laser ablation of an iron target in water (PLAL) and in air (PLAG) using a Nd:YAG laser (1064 nm, 20 Hz, 7 ns, and 150 mJ) resulted in spherical MNPs of 2–80 nm containing Fe_3_O_4_, α-Fe_2_O_3_, γ-Fe_2_O_3_, FeO, and Fe. The magnetic properties were studied using VSM [406]. NiO NPs were synthesized using a 355 nm nanosecond laser by ablating a Ni target immersed in a 3% H_2_O_2_ solution and characterized regarding their structure, morphology, and composition [407]. Tsuji et al. synthesized NPs of Co, CoO, and Co_3_O_4_ by ablating targets in water and hexane, and found that the composition of NPs can be controlled by selecting the solvents in PLAL [408]. Quantum-confined, crystalline Co_3_O_4_ NPs (<5 nm) with high electrocatalytic oxygen-evolution activity were synthesized by PLAL [334].

**Figure 38:**
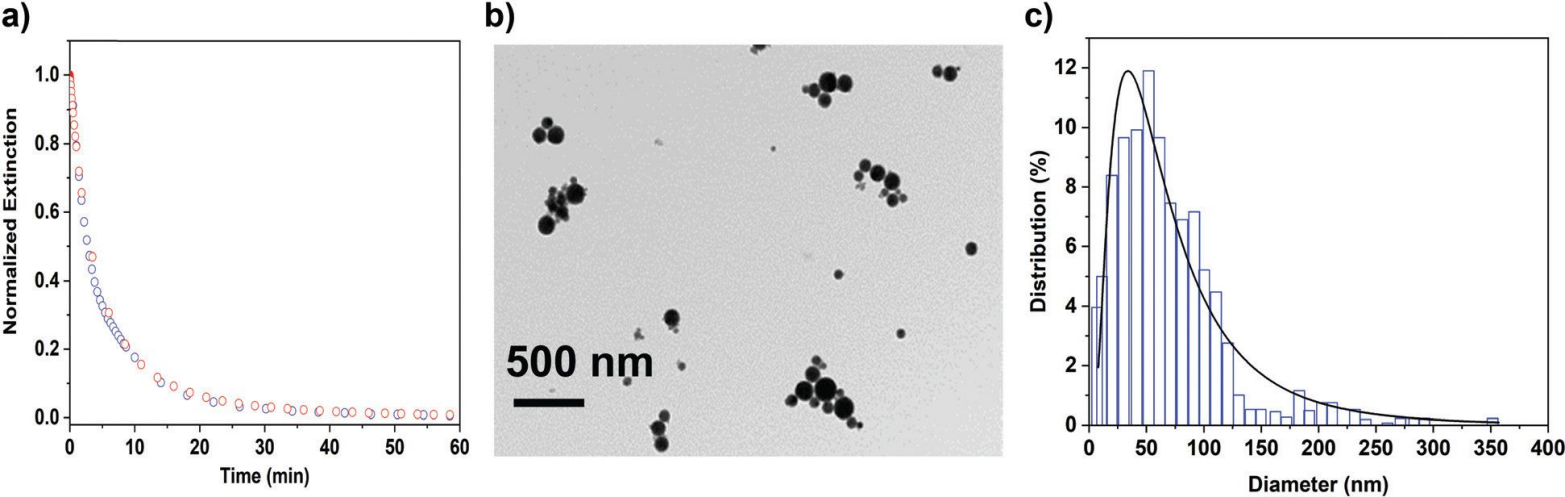
(a) The experimental magnetophoretic curve (red circles) with the fitting (blue circles) obtained with a magnetization of 11 A·m^2^·kg^−1^. (b) TEM images of the NPs and (c) dimensional distribution of 400 NPs synthesized by laser ablation of strontium ferrite. Figure 38 was used with permission of the Royal Society of Chemistry, from [409] (“Synthesis of magnetic nanoparticles by laser ablation of strontium ferrite under water and their characterization by optically detected magnetophoresis supported by BEM calculations” by V. Piotto et al., *J. Mater. Chem. C*, vol. 10, issue 10, ©2022); permission conveyed through Copyright Clearance Center, Inc. This content is not subject to CC BY 4.0.

Nanoparticles of strontium ferrite synthesized by laser ablation of a strontium ferrite target in water were characterized regarding their magnetic properties using optically detected magnetophoresis, which exploits the motion of NPs in a fluid under a magnetic field gradient monitoring the optical extinction of NPs with absorption (Figure 38) and scattering contributions [409].

Nanoparticles of BaFe_12_O_19_ were synthesized by laser ablation in different solvents using 532 and 1064 nm ablation, and they were characterized regarding structure, morphology, composition, and optical properties. The nature of the solvent and the ablation wavelength were crucial for the particle sizes in the nanofluid. Using VSM studies, magnetic properties were explored at room temperature and low temperatures [410]. These nanofluids also showed good microwave absorption properties in the S and X bands [411].

**Figure 39:**
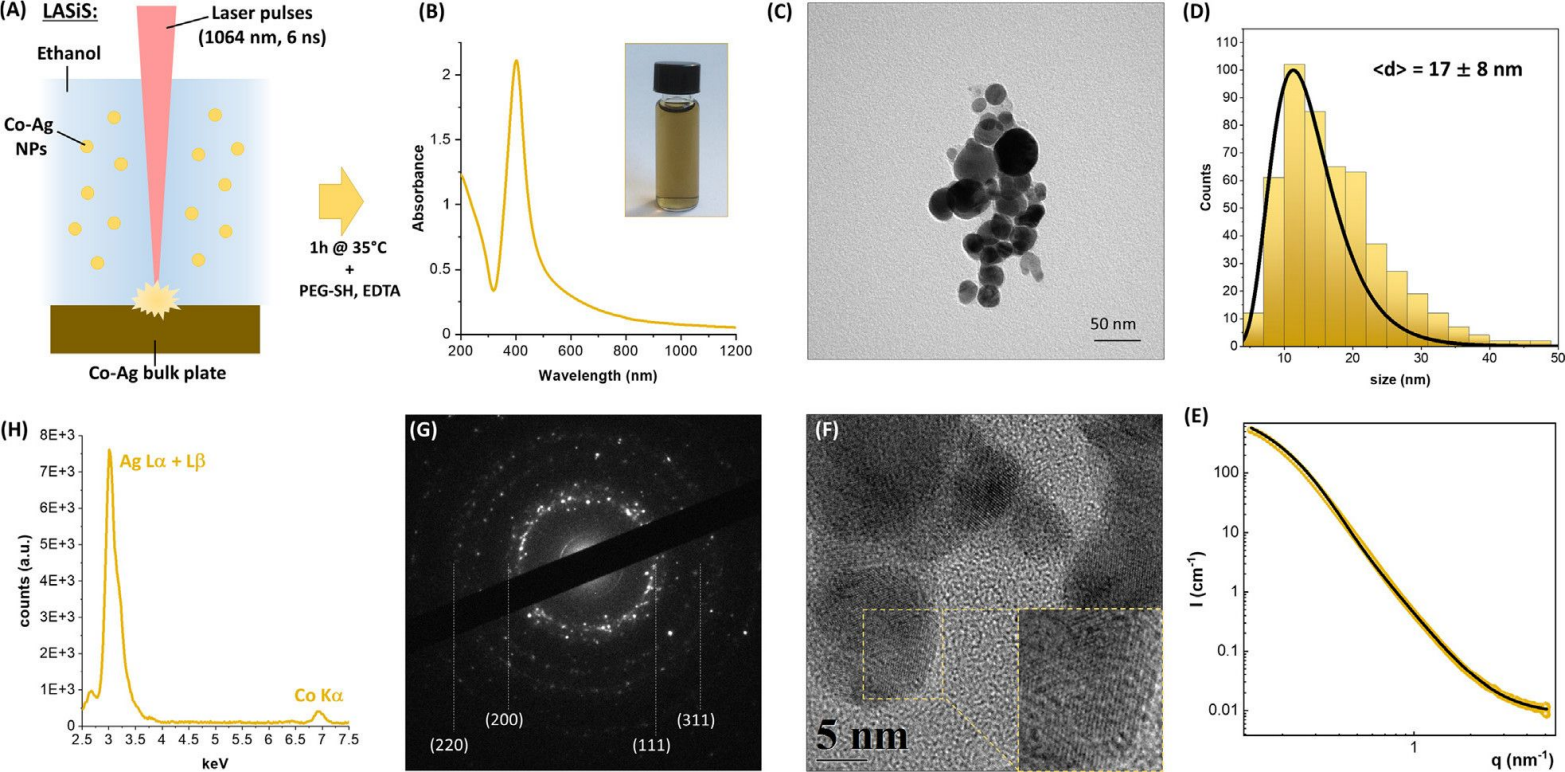
(A) Sketch of LASiS procedure. (B) UV–vis of PEG-coated Co-Ag NPs. Inset shows a picture of the same colloid. (C, D) Representative TEM image and size histogram of Co–Ag NPs. (E) SAXS curve for the PEG-coated Co–Ag NPs and size distribution obtained by curve fitting. (F) HRTEM image of Co–Ag NPs, with lattice fringes corresponding to (111) interplanar distance in Ag. (G) SAED pattern of Co–Ag NPs, showing the reflections proper of Ag F.C.C. (H) EDX spectrum collected on the same NPs, showing Ag and Co characteristic peaks. Figure 39 was reprinted from [412], *Journal of Colloids and Interface Science*, vol. 585, by A. Guadagnini; S. Agnoli; D. Badocco; P. Pastore; D. Coral; M. B. Fernàndez van Raap; D. Forrer; V. Amendola, “Facile synthesis by laser ablation in liquid of nonequilibrium cobalt-silver nanoparticles with magnetic and plasmonic properties”, pages 267-275, Copyright (2021), with permission from Elsevier. This content is not subject to CC BY 4.0.

Synthesis of NPs containing multimetallic structures can be useful in areas like catalysis, sensing, energy conversion and storage, or tools for biomedical analysis and treatments. Ag–Co NPs were synthesized by 1064 nm ablation of a bimetallic target composed of 50 atom % Ag and 50 atom % Co in ethanol, and the resulting Ag–Co NPs substantially maintained the plasmonic response of silver and the ferromagnetism of Co [412]. Morphology, size, and composition analyses of the laser-synthesized Ag–Co NPs are included in Figure 39.

Hybrid Fe_3_O_4_@Au and Fe_3_O_4_@Ag MP NPs fabricated combining LAL and solvothermal methods showed multiwavelength SERS properties. Their SERS properties were tested with 4-mercaptobenzoic acid and adenine using 633 and 785 nm laser excitations and achieving a limit of detection of 10^−7^ M [385].

These studies on synthesis of different magnetic NPs as well as MP NPs by laser ablation and their applications in many other areas of interest are to be explored further.

### 4 Challenges and scope of the devices from laser-generated nanocolloids

LPL has emerged as a transformative technique for the synthesis of NPs in colloidal form, offering control over particle size, shape, and surface properties. Methods such as LAL, LFL, LML, LIL, and LDL have enabled the fabrication of a wide array of nanostructures with applications across diverse fields such as catalysis, energy storage, and optoelectronics. These LPL techniques allow for the generation of complex nanostructures, including bimetallic alloys, submicrometer spheres, QDs, and a wide range of materials, from metals to semiconductors, oxides, and carbides, without the need for additional chemicals or surfactants. LAL is one of the most widely studied techniques, as it provides a simple and effective method for NP synthesis by focusing a laser beam on a target material submerged in a liquid, resulting in the generation of NPs through vaporization and subsequent condensation of the ablated material. This technique is advantageous because of its ability to create high-purity particles with minimal contamination. LFL is especially beneficial as a post-processing tool for NPs produced by LAL, providing an advanced technique for the fabrication of SMSPs through the selective melting of raw colloidal NPs. LFL has demonstrated the ability to produce ultrasmall NPs and sub-nanometer atomic clusters, which hold significant potential for the applications mentioned above. Further research is needed to address existing challenges, such as oxidation and limitations related to pulse intensity, while also enhancing the efficiency and scalability of the process [413–416]. A deeper understanding of the fragmentation mechanisms and optimization of experimental parameters will enable the tailored synthesis of NPs. LML involves the use of pulsed laser irradiation to heat and melt NPs in a liquid medium, resulting in the formation of droplets that solidify into highly crystalline, non-porous SMSPs. Additionally, the technique’s efficiency improves with high-power laser irradiation, where the use of picosecond lasers at low fluence is particularly effective in minimizing particle evaporation while producing high-quality SMSPs. The use of burst-mode laser pulses and the cooling rate of the particles after laser heating have been shown to effectively influence the heating process and to reduce byproduct formation, improving the overall efficiency of particle synthesis. Moreover, LML enables the creation of complex particle structures, such as core–shell configurations or alloy particles by exploiting the differences in the heating behavior of each component during laser irradiation. Despite these advancements in all these techniques, challenges remain in improving productivity, achieving narrower size distributions, and further understanding the interactions between laser, particle, and liquid medium. Looking ahead, substantial research opportunities lie in optimizing experimental setups and refining laser parameters, such as fluence and pulse duration, to enhance the scalability, efficiency, and uniformity of the processes. A key area of future work will be to explore the role of liquid properties also, such as refractive index and thermal conductivity, which are less discussed in current literature but can significantly impact nanoparticle formation. Furthermore, the development of hybrid methods that combine laser processing with other nanoparticle synthesis techniques could provide a more precise control over material properties, facilitating the creation of advanced nanostructures with tailored functionalities. The integration of in situ characterization methods will also provide deeper insights into the LPL process, paving the way for more sophisticated, high-performance nanomaterials and hybrid devices.

The potential use of laser-generated NPs in thin film fabrication continues to expand, with the promise of enhancing device performance and functionality when it comes to applications in photovoltaics, optoelectronic devices, catalysis, and sensors. This review has focused on various deposition methods such as spin coating, drop casting, spray deposition, spin-dip coating, and EPD, which influence the morphology and properties of the resulting films. Each method of film fabrication discussed presents a unique approach to achieving precise control over film thickness, morphology, and adhesion, which is essential for specific applications. Laser-assisted colloidal synthesis offers a sustainable and promising approach for producing surfactant-free NPs that can be directly deposited onto substrates for thin film deposition leading to high-quality, functional thin films. Challenges related to deposition precision and scalability, particularly with techniques like drop casting, remain a focus for future research. The development of novel deposition methods, such as substrate vibration-assisted drop casting and laser-assisted chemical vapor deposition, alongside inkjet printing and roll-to-roll processing could address and offer enhanced control over film characteristics, scalability, and cost-effectiveness. The integration of these methods with techniques like atomic layer deposition and molecular beam epitaxy may also pave the way for unlocking new possibilities for applications in electronics, sensors, and coatings. Further research into these hybrid techniques will likely lead to more efficient and functional thin films tailored for next-generation electronic and energy devices, driving forward the potential of LPL as a reliable, industrial-scale solution for nanomaterial production.

Laser-generated colloids have been explored as a promising technique in thin film fabrication for device applications. The NPs synthesized via laser techniques offer superior optical properties and tunability, significantly improving the sensitivity, responsivity, and efficiency of photodetectors. This advancement facilitates the development of a range of photodetector configurations, from photoconductive detectors to heterojunction-based devices, designed for specific applications in optoelectronics, imaging, and energy harvesting. The integration of laser-synthesized nanocolloids into hybrid materials and novel architecture promises to open new possibilities for multifunctional, wavelength-selective photodetectors. Furthermore, the exploration of other optoelectronic applications, including non-linear optics and SERS sensors, could further solidify the role of laser-generated colloids in advanced technological applications. Laser-assisted colloidal synthesis also offers a promising, sustainable approach to enhancing PV devices. Materials such as metal oxide, perovskite, and Si NPs demonstrate enhanced light absorption, carrier mobility, and improved overall device performance. The integration of laser-generated NPs into various solar cell architectures, including thin-film and dye-sensitized solar cells, has shown significant improvements in efficiency, optical response, and stability. Looking ahead, the continued exploration of laser-based techniques for synthesizing a broader range of NPs, including advanced composites and hybrid materials, is crucial for developing next-generation solar cells. Future research should focus on understanding the effects of NP integration on device longevity and exploring new applications such as photon upconversion and light-trapping strategies. In the environmental domain, NPs have been functionalized to address pressing issues, such as pollution remediation and the development of cost-effective energy sources. The ability to engineer the composition and properties of NPs has enabled the creation of novel tools and techniques that can effectively remove pollutants from water and air, as well as to improve the efficiency of energy generation and storage [413,417–418].

Challenges such as precise control over nanoparticle size and composition are still a hurdle especially in ternary and quaternary solar cells (i.e., CIGS, CAS, CZTS), which are commercially used and have high efficiency. Exploration of new materials, like layered and plasmonic materials, MXenes, and MOFs, for addressing issues like exciton quenching from ligand coatings and improving large-scale production is needed. Perovskites use abundant elements (lead-based perovskites are most common, though lead-free options are being explored), and their low material requirements reduce overall resource demands compared to thick silicon wafers. By adjusting laser parameters such as energy, pulse number, and solvent choice, researchers can finely tune NP size, shape, and composition, optimizing their integration into diverse photodetector architectures like photoconductive detectors, photodiodes, photovoltaic sensors, and heterojunction photosensors. Despite these advances, challenges remain in scaling up the nanoparticle production, optimizing reactor designs, and ensuring long-term catalyst stability. However, researchers are actively developing strategies such as incorporating co-catalysts, engineering heterostructures, and refining reactor configurations to overcome these barriers. Overall, laser-generated photocatalysts represent a transformative innovation, offering a powerful, customizable, and sustainable approach to addressing global energy and environmental challenges.

Looking ahead, laser-assisted synthesis techniques hold tremendous promise for the future development of advanced PEC and electrocatalytic materials, particularly by enabling precise control over composition, morphology, and interface engineering. Future research will likely focus on expanding the design of hybrid oxide–sulfide layered heterostructures, leveraging the complementary strengths of each material class, that is, combining the chemical stability of oxides, the visible-light activity of sulfides, and the high conductivity and abundant active edge sites of layered materials like MoS_2_ and WS_2_. Furthermore, advances in ultrafast laser processing could enable novel defect engineering strategies and phase transformations that unlock new catalytic pathways. A critical outlook involves addressing current challenges such as the long-term operational stability of sulfide systems, efficient charge transfer across complex interfaces, and the scalable integration of laser-fabricated nanomaterials into practical device architectures. Additionally, combining laser techniques with machine learning-guided optimization may accelerate the discovery of ideal laser parameters for targeted material modifications. Overall, the continued evolution of laser-assisted synthesis will not only push the boundaries of photoelectrocatalytic and electrocatalytic performance but also open new frontiers for sustainable energy conversion, storage, and environmental remediation technologies.

The development of innovative metallic and bimetallic alloys (such as Ag–Au, Au–Cu, or Pt-based composites) presents exciting opportunities for SERS substrates. These novel materials can combine noble metals’ strong enhancement factors with increased chemical stability, mechanical strength, and biocompatibility. Alloying can also provide synergistic benefits by tailoring plasmonic characteristics to certain wavelengths or applications (for example, biomedical vs environmental sensing). Future SERS sensors will benefit from precise control over material composition at the nanoscale. Adjusting the metal ratios allows researchers to fine-tune plasmon resonance peaks, surface reactivity, and chemical affinity for target molecules. This tunability provides selective detection, allowing sensors to be customized for specific analytes or environmental conditions.

Scaling up from lab-scale substrates to large-area, uniform SERS platforms is a primary objective. Future production methods for wafer-sized or flexible SERS films will include laser scribing, nanoimprinting, self-assembly, and chemical templating. Large-area sensors are critical for practical applications such as environmental monitoring, medical diagnostics, and food safety, which need extensive sample coverage and reliable signal response. A major problem is to produce SERS sensors that can be regenerated or cleaned for repeated use without losing sensitivity. Future designs may include self-cleaning, strong chemical treatments, or protective coatings that allow the sensor to work consistently across several detection cycles. Reusability reduces costs and makes SERS more sustainable in industrial and therapeutic settings. Future SERS substrates must withstand oxidation, corrosion, and degradation over time, especially under real-world conditions (humidity, temperature, and biological media).

While SERS can now identify single molecules in controlled environments, the future lies in developing dependable, reproducible ultrahigh sensitivity in actual scenarios. Advances in nanostructure design (such as nanopillars, nanogaps, or hot-spot engineering), combined with machine learning-based signal processing, might extend detection limits and increase quantification of low-abundance analytes in complex samples. Traditional lithographic technologies (such as electron beam lithography) are accurate, yet costly and time-consuming. Laser-based patterning techniques (such as femtosecond laser ablation, laser-direct writing, and laser scribing in liquid) provide a low-cost, scalable, and efficient method for creating SERS-active nanostructures. These technologies enable direct, maskless writing of plasmonic patterns on flexible or curved substrates, paving the way for customized and on-demand sensor manufacturing.

Exploring new alloy nanomaterials and their varying compositions by LPL could be an option to discover newer magnetic nanostructures. Scopes of magneto-plasmonic nanostructures are to be explored further combining the plasmonic properties of alloys of two or more metal nanostructures and their hybrids with magnetic oxide nanomaterials. Also, exploring the synthesis and properties of nanostructures that can be generated from plasmonic and magnetic components could be one of the areas for future explorations.

The potential of LPL looks promising, particularly as more sophisticated laser systems and an enhanced understanding of laser–particle–liquid interactions pave the way for more efficient, sustainable, and precise nanoparticle synthesis. Additionally, hybrid techniques that combine LAL, LFL, LML, LDL, and LIL with other synthesis methods could offer enhanced control over the resulting nanomaterials, especially in the fabrication of thin films and coatings. As these challenges are addressed, the different techniques will likely play an increasingly significant role in the fabrication of novel nanostructures and hybrid materials for diverse technological innovations.

## Data Availability

Data sharing is not applicable as no new data was generated or analyzed in this study.
